# Reliable anti-cancer drug sensitivity prediction and prioritization

**DOI:** 10.1038/s41598-024-62956-6

**Published:** 2024-05-29

**Authors:** Kerstin Lenhof, Lea Eckhart, Lisa-Marie Rolli, Andrea Volkamer, Hans-Peter Lenhof

**Affiliations:** 1 Center for Bioinformatics, Chair for Bioinformatics, Saarland Informatics Campus (E2.1) Saarland University, Campus, 66123 Saarbrücken, Saarland Germany; 2 Center for Bioinformatics, Chair for Data Driven Drug Design, Saarland Informatics Campus (E2.1) Saarland University, Campus, 66123 Saarbrücken, Saarland Germany

**Keywords:** Reliability, Conformal prediction, Simultaneous regression and classification, Drug sensitivity prediction, Drug prioritization, Cancer, Computational models, Machine learning, Computational biology and bioinformatics, Targeted therapies, Cancer therapy

## Abstract

The application of machine learning (ML) to solve real-world problems does not only bear great potential but also high risk. One fundamental challenge in risk mitigation is to ensure the reliability of the ML predictions, i.e., the model error should be minimized, and the prediction uncertainty should be estimated. Especially for medical applications, the importance of reliable predictions can not be understated. Here, we address this challenge for anti-cancer drug sensitivity prediction and prioritization. To this end, we present a novel drug sensitivity prediction and prioritization approach guaranteeing user-specified certainty levels. The developed conformal prediction approach is applicable to classification, regression, and simultaneous regression and classification. Additionally, we propose a novel drug sensitivity measure that is based on clinically relevant drug concentrations and enables a straightforward prioritization of drugs for a given cancer sample.

## Introduction

The major goal of personalized medicine is to individually tailor treatments to patients based on their pheno- and genotypic characteristics. In cancer therapy, treatment customization can, however, be challenging because of the heterogeneity of cancer that profoundly affects the efficacy of anti-cancer drugs.

Machine learning (ML) methods have been developed to elucidate the relationship between the molecular properties of cancer cells and observed drug responses and, based on these insights, to optimize anti-cancer drug therapies^1–4^. Usually, these ML methods are trained on data from model systems such as patient-derived xenografts or cancer cell lines^1,2^. When applying trained ML models to a previously unseen cancer sample, the ultimate goal is to identify the effective drugs and to sort these compounds by their predicted efficiency, i.e. to perform drug prioritization^5^. To achieve this goal, current research focuses on the more easily manageable sub-tasks of developing classification approaches able to identify sensitive samples for a given drug (e.g.,^6–8^) and regression approaches able to quantify the sensitivity of sample to a drug (e.g.,^9–13^), which are known as drug sensitivity prediction tasks. For each drug, the trained models usually predict a single class or single continuous drug response value for one sample, i.e. they return so-called point predictions. By comparing these point predictions to the known actual values, the overall model performance during training, validation, and testing can be assessed using conventional error measures. While this can already create a certain level of trust in predictions, applications in real-world healthcare scenarios where the true response is unknown (i.e., no such metric could be evaluated) require the *reliability* of predictions^14–16^. Here, *reliability* is defined as the level of trust that one can have in a prediction for a previously unseen instance during the deployment phase of the ML algorithm. It can, for example, be established via p-values or certainty guarantees. Information on the reliability of the prediction of ML-based drug sensitivity predictions is typically not provided (cf. Table 1 in Supplement 1 for a thorough literature review of reliability estimation in drug sensitivity prediction). Consequently, we have no information whether a predicted value is likely to be close to its real but unknown value.

From the related work (cf. Table 1 in Supplement 1), only Fang et al. recently developed an approach to estimate the reliability of a prediction: they suggested a random forest (RF)-based quantile regression approach to estimate prediction intervals instead of point predictions^17^. Intuitively, the width of the interval defined by two quantile predictions indicates the reliability of the prediction (the narrower, the better). While their approach highlights the significance of reliability estimation in the healthcare domain, it is limited to RF regression. Moreover, their approach does not give a reliability guarantee or confidence level. In drug discovery and toxicity prediction, conformal prediction (CP) recently gained popularity^18–21^. Conformal prediction provides a mathematical rigorous certainty estimation framework that can sit on top of various machine learning regression or classification approaches^22–24^. Given a user-specified maximal allowed error rate $\alpha$ and a trained regression or classification model, CP applied to a new sample returns the set of classes (classification) or the interval (regression) that contains the true value with a certainty of almost exactly $1- \alpha$ instead of a simple point prediction. Consequently, single-element sets and narrow intervals correspond to a high certainty, and the sizes of the sets and intervals typically increase with growing uncertainty about the prediction. A prerequisite to being fulfilled by a model serving as input to CP is that it delivers a notion of (un)certainty about its predictions, e.g., for RF classifiers, certainty may be represented by the proportion of trees that voted for the predicted class and for RF regressors, quantile regression forests can be employed.

**Figure 1:**
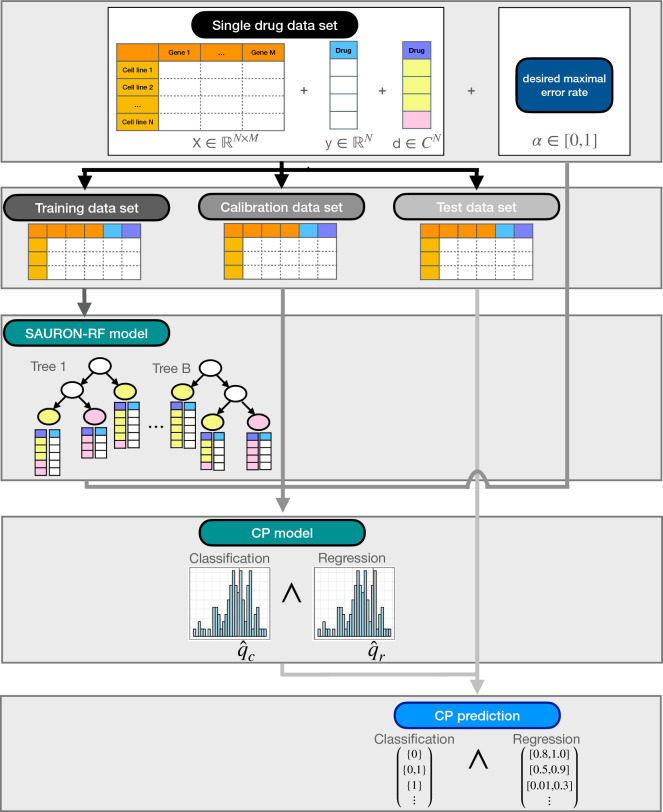
*CP pipeline.* This figure depicts how CP can help to perform reliable (simultaneous) regression and classification. At first, the given drug data set has to be split into three disjoint data sets: a training, a calibration, and a test set. The ML method, e.g., SAURON-RF, is then trained on the training data set. Afterwards, the resulting model is applied to the calibration data to derive a distribution of (un)certainty of the predictions. Together with the user-specified maximal allowed error rate $\alpha$, this distribution is used to define a threshold that when appropriately applied to the test data set guarantees a certainty of $1-\alpha$ of the test set predictions.

**Figure 2:**
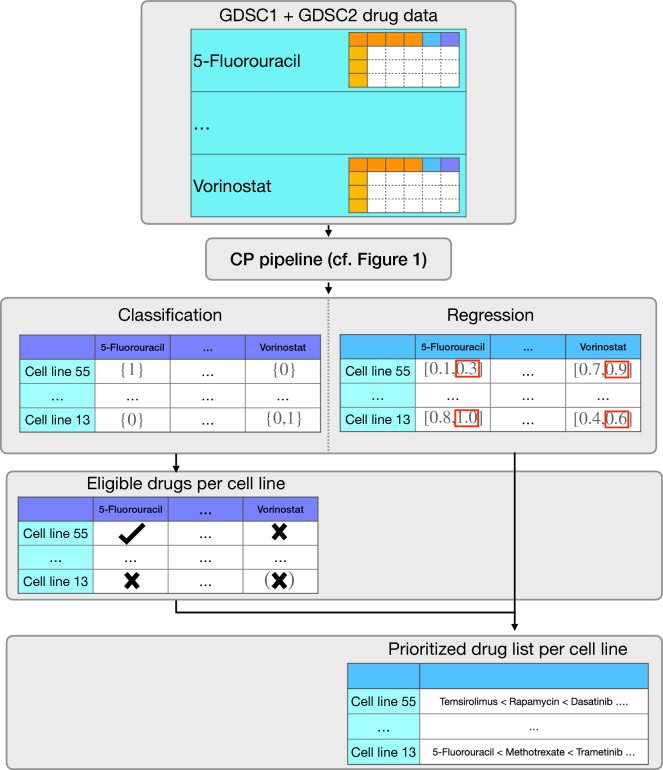
*Drug prioritization pipeline.* This figure shows a drug prioritization pipeline originating from combining the CMax viability with SAURON-RF and CP. The output of the CP pipeline (cf. Fig. 1) deployed with our SAURON-RF method are sets for the classification task and intervals for the regression task. Here, sets that contain only one element indicate that we can be confident about the initial point prediction (single class) of the trained model. Thus, we can identify effective drugs by filtering for sets solely comprising the class corresponding to drug sensitivity (1: sensitive). Due to the across-drug comparability of the CMax viability, we can rank these drugs by their predicted efficiency using, for example, the upper limit of the regression interval that represents a value not being surpassed with high probability.

In this paper, we introduce conformal prediction into the anti-cancer drug sensitivity domain. To this end, we propose a novel flexible conformal prediction approach for drug sensitivity prediction and prioritization (cf. Figs. 1 and 2). Our Python framework is applicable to classification, regression, and, most notably, even joint classification and regression methods, which we have demonstrated to outperform classification or regression alone^25^. We exemplify the capabilities of our CP pipeline by combining it with our state-of-the-art approach SAURON-RF (SimultAneoUs Regression and classificatiON Random Forests) and by applying the resulting novel method to the GDSC (Genomics of Drug Sensitivity in Cancer) data set, which is one of the largest publicly available pharmacogenomic databases^26–28^.

As mentioned earlier, a model has to provide a notion of (un)certainty to be eligible for CP. For SAURON-RF, this notion is immediately accessible for the classification part: we can simply employ the proportion of trees that voted for the predicted class. For the regression part, we had to define and implement a notion of (un)certainty. To this end, we extended SAURON-RF with a quantile regression functionality adapted from the quantile regression algorithm by Meinshausen^29^.

To carry out the drug prioritization with our CP pipeline, a drug sensitivity measure that is comparable across drugs is necessary. Established measures, e.g. the IC50 and AUC values, are only comparable across cell lines but not across drugs. Therefore, we propose the CMax viability, a novel drug sensitivity measure based on the highest, clinically relevant drug dose as described in^30^. We define drug prioritization as the task of identifying and subsequently sorting the list of effective drugs. As such it is similar in spirit to drug recommendation as introduced by He et al.^31^: they define drug recommendation as the task of correctly ranking the *v* most efficient drugs. However, performing drug recommendation as described above suffers from two major drawbacks compared to our approach. Firstly, methods such as KRL by He et al.^31^ and PPORank by Liu et al.^32^ cannot evaluate whether they only identified effective drugs, i.e., the *v* most efficient drugs can already contain ineffective ones. Secondly, they cannot quantify differences of efficiency since they do not predict sensitivities directly. Apart from that, neither KRL nor PPORank provide certainty estimations.

The major contributions of this manuscript are:

1. We address the demand for reliability of drug sensitivity predictions, i.e., we give guarantees for the correctness of a prediction for a previously unseen sample under specific conditions (cf. Section "Conformal prediction pipeline").
2. To this end, we developed a pipeline for conformal prediction that is applicable to any machine learning algorithm given that it provides some notion of uncertainty, e.g., class probabilities for classification or quantiles for regression.
3. We developed a novel drug sensitivity measure, the CMax viability, which allows for a comparison between drugs as opposed to commonly used measures such as IC50. Consequently, we can prioritize recommendable drugs, which is required for medical decision support.
4. We extend SAURON-RF with a quantile regression algorithm and a multi-class formulation.
5. Finally, we combine the CMax viability with extended SAURON-RF and the CP-pipeline to achieve not only reliable drug sensitivity prediction but also reliable drug prioritization, which has also not been described in the drug sensitivity prediction literature before (cf. Table 1 Supplement 1).

With an extensive evaluation across the complete GDSC database, we show that our novel combined approach is superior to state-of-the-art methods, inlcuding SAURON-RF without CP. Moreover, we ultimately perform drug priorization. We demonstrate that we can identify effective drugs and subsequently prioritize them with SAURON-RF. Beyond that, CP can substantially improve our predictions. In particular, CP not only provides guarantees for our predictions, but it successfully diminishes false predictions while retaining correct ones and can help prioritize effective drugs. In particular, we could eliminate 52% of the remaining 19% ineffective drugs falsely suggested (False Positives) by SAURON-RF. In total, we thus achieved a median overall 92% correctness (precision) of our prioritized drug lists. The reduction of false predictions for CP is usually accompanied by a reduction of true predictions. Still, the prioritized drug list contained the most efficient drug in 75% of cases, and the distance between the first drug in our list and the most efficient drug is within a 10% range of the CMax viability scale for 62% of cell lines.

## Results

A prerequisite for translating ML models into healthcare decision support systems is to create trust in their predictions. To address this demand for drug sensitivity prediction and prioritization, we developed and implemented a conformal prediction (CP) approach. Since we have shown that simultaneously performing regression and classification can outperform regression and classification alone^25^, the presented CP pipeline is able to handle classification, regression, and joint classification and regression tasks.

In the following, we first briefly describe the used data from the GDSC. Here, we also define our novel drug sensitivity measure called *CMax viability* that enables a comparison of the drug response values not only between cell lines but also between drugs. Then, we present the CP pipeline as well as its drug sensitivity prediction and drug prioritization capabilities.

### Data set processing and definition of novel drug sensitivity measure

For all our analyses, we used the Genomics of Drug Sensitivity in Cancer (GDSC) data set. In particular, we downloaded the processed gene expression data, the pre-computed logarithmized IC50 drug responses, and the raw drug responses (see Section "Online methods" for further details). In addition to a feature matrix corresponding to the gene expression matrix downloaded from the GDSC, SAURON-RF requires a continuous and discrete drug response vector per drug as input. For the downloaded continuous IC50 data, we derive a discretized response vector per drug by applying a procedure introduced by Knijnenburg et al.^7^ using a custom R-script described previously^8^. Established measures such as the IC50 and AUC values, as provided by the GDSC, are only comparable across cell lines but not across drugs. Since therapeutically feasible concentration ranges of drugs can differ substantially, a smaller IC50 for one drug compared to another does not necessarily indicate a stronger sensitivity. On the contrary, an IC50 of one drug may be lower than the IC50 of another drug and still be out of the therapeutically feasible range. Likewise, the choice of concentration range over which the dose-response-curve is integrated can strongly impact and bias resulting AUC values. In Table 2 of Supplement 1, we exemplary depict a prioritization of drugs for one particular cell line resulting from a sorting by IC50 values: 6 out of the 8 highest ranked drugs are out of the feasible concentration range.

To address such issues, we propose a novel drug sensitivity measure called CMax viability. We define the CMax viability of a drug as the viability at the CMax concentration, which corresponds to the peak plasma concentration of a drug after administering the highest clinically recommended dose^30^. The CMax viability can take values in the range [0, 1]. Here, 0 corresponds to no viability of cancer cells after treatment, and 1 indicates 100% viability. To derive the viability of a cell line at the CMax concentration, i.e., the CMax viability for a drug-cell line combination, we determine the intersection point between the dose-response curve of the cell line calculated from the raw GDSC response data and the line parallel to the viability (Y) axis passing through the CMax concentration. To calculate the CMax viability in practice, we downloaded a list of CMax concentrations from^30^. For each drug-cell line combination, we compute the intersection point. This yields one response vector per drug containing the CMax viabilities for all considered cell lines, which are comparable not only across cell lines but also across drugs. Consequently, the CMax viabilities enable drug prioritization. Moreover, since the measure is based on clinically relevant drug concentrations, it may facilitate the translation of findings into clinical application. To discretize these values, we exploit the across-drug comparability and apply the partitioning around medoids (PAM) algorithm to all CMax viabilities across all drugs simultaneously. We then either determine one discretization threshold (two classes: sensitive (1) and resistant (0)) or two thresholds (three classes: sensitive (1), ambiguous (2), resistant (3)) applicable to all drugs. A more detailed description of the data set compilation is provided in the Methods section (cf. Section "Online methods").

### Conformal prediction pipeline

CP represents a mathematical rigorous certainty estimation framework applicable to all regression and classification ML methods provided that the latter supply a notion of (un)certainty. Given a user-specified maximal error rate $\alpha \in [0,1]$, CP returns prediction sets (classification) or intervals (regression) that contain the true response with a certainty, also known as coverage, of almost exactly $1-\alpha$. This is called marginal coverage property (see Section "Conformal prediction" for more information). We designed and implemented a flexible CP framework in Python that can be readily used for regression, classification and joint regression and classification methods, e.g., SAURON-RF. In Fig. 1, we give an overview of the developed CP pipeline applied to SAURON-RF. In the following, we describe how CP in general and, in particular, our Python framework can be leveraged to create trust in ML-based models. To this end, we first outline the functionality of our framework and then discuss the results when combined with SAURON-RF. The required extensions to the methodology of SAURON-RF are explained in the Methods section (see Section "Extension of SAURON-RF").

#### Input of CP

In ML, we usually assume that our samples are drawn i.i.d. to guarantee the claimed properties of our methods. For the CP guarantee to hold, we only need to assume exchangeability of the data, which is a slightly weaker assumption^24^. The training and testing of supervised ML methods typically requires at least two disjoint data sets: a training data set for parameter selection and a test set for the final evaluation of the trained model. CP demands a third disjoint data set, the so-called calibration data set, used to calculate statistics on the (un)certainty of the model. Accordingly, the trained model needs to provide a notion of uncertainty (or certainty). In the case of SAURON-RF, we employ $1- \frac{\#\text {trees that voted for predicted class}}{\#\text {trees}}$ as a measure of uncertainty for classification. For regression, we quantify the dispersion of response values with quantile regression (cf. Section "Extension of SAURON-RF" for a detailed description of the newly developed quantile SAURON-RF algorithm). In addition to this notion of uncertainty, the user has to specify a maximal allowed error rate $\alpha$, which allows for a flexible control of the certainty. If a strict error rate cannot be met by a model, increasing $\alpha$ might still help to identify the most reliable trends.

#### Score functions

CP integrates the given notion of uncertainty in a score function, often also called a non-conformity score. By applying the score function to the calibration data set, a score distribution can be generated. For classification, we implemented three different score functions: True-class (TC), Summation (Sum), and Mondrian (Mon). For regression, we implemented a score function called Quantile (Qu). In Sections "Classification scores and Regression score", we give their exact definitions. It is possible to implement all of the mentioned score functions as uncertainty measures, i.e., high values correspond to high uncertainty, and low values correspond to high certainty. Given a score distribution and the maximal allowed error rate $\alpha$, CP derives a threshold ${\hat{q}}$ that is a modified $(1-\alpha )$-quantile of the score distribution if the score function quantifies uncertainty (see Section "Conformal prediction" for details).

#### Output of CP

After training the ML model on the training data set, and employing its notion of uncertainty in a score function to derive a score distribution on the calibration data set, the CP output for the test set can be generated. The trained ML model has to be applied to the test set, and the score function must also be evaluated. By combining ${\hat{q}}$ with the derived score per test set sample (see Section "Conformal prediction" for details), the point prediction of the ML model can be exchanged with a valid prediction set (classification) or interval (regression). More specifically, CP returns prediction sets (classification) or intervals (regression) that fulfil the marginal coverage property. Some scores guarantee special versions of this property. The Mondrian score, for example, provides this coverage for every ground truth class, which is especially desirable when there is a considerable class imbalance present, as is the case for drug sensitivity prediction in cancer^25^. Our CP pipeline can not only return prediction sets or intervals but also both simultaneously, making it amenable to joint classification and regression methods such as SAURON-RF. Moreover, when combining this capability with our novel drug sensitivity measure that is comparable across drugs, we can leverage the full potential of SAURON-RF and ultimately perform drug prioritization: we can first identify effective drugs (classification) and then rank them by their predicted efficiency (regression). We depict this application scheme in Fig. 2.

### Drug-centric analysis: drug sensitivity prediction

**Figure 3:**
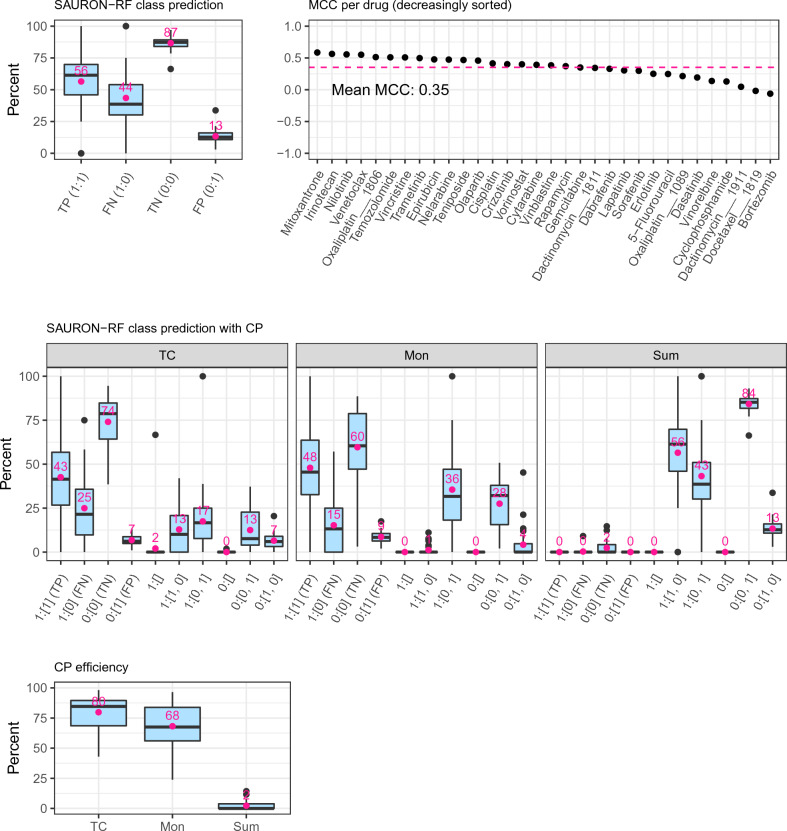
*Classification test set performance GDSC2.* The upper row of this figure depicts the classification performance of SAURON-RF across the different drugs from GDSC2. The notation on the x-axis of the first plot consists of a tuple containing the true class as first element and the predicted class as second element. For all predictions where the true class is sensitive (i.e., TP or FN), percents are calculated by dividing by the number of all sensitive cell lines (TP + FN). Likewise, for all predictions where the true class is resistant (i.e., TN or FP), percents are calculated by dividing by the number of all resistant cell lines (TN + FP). Thus, the x-axis labels correspond to the well-known confusion matrix metrics called sensitivity = $\frac{\text {TP}}{\text {TP + FN}}$, miss-rate = $\frac{\text {FN}}{\text {TP + FN}}$, specificity = $\frac{\text {TN}}{\text {TN + FP}}$, and fall-out = $\frac{\text {FP}}{\text {TN + FP}}$, respectively. The middle row shows the effects of CP on the performance in terms of true positive/negative predictions. Again, tuples of the true and the predicted class sets are shown on the x-axis and percents were obtained as described above. In Supplement 1 Section 7, we provide all formulas. In the lower row of this figure, the CP efficiency is presented.

**Figure 4:**
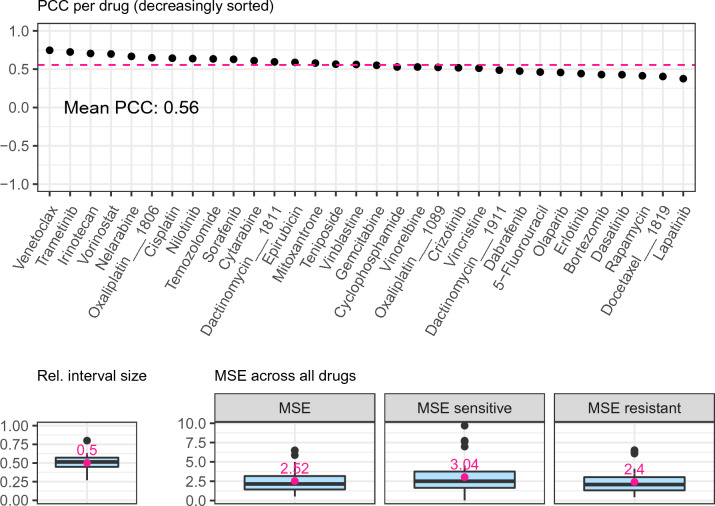
*Regression test set performance GDSC2.* The upper row of this figure depicts the Pearson correlation coefficient between the actual continuous response values and the predicted continuous response values for all drugs. The lower row shows the mean-squared error (MSE) and the interval width of the CP Quantile regression score relative to the spanned training ranges of the drugs.

**Figure 5:**
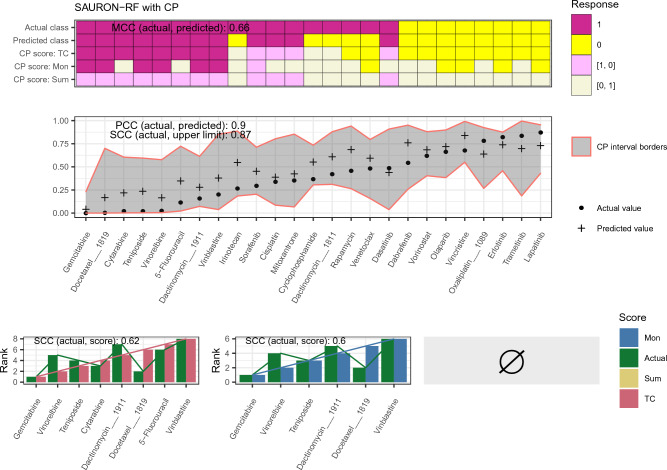
*Prioritization example GDSC2.* This figure exemplifies the performance of our prioritization pipeline (cf. Fig. 2) when applied to one particular cell line (COSMIC ID 1240154) from the test set of the GDSC2 data set. The upper plot visualizes the classification performance with and without CP for all analyzed drugs. The middle plot depicts the regression result for all drugs, including the $90\%$ CP interval, and the lower plot shows the resulting prioritized drug lists with the drugs ascendingly sorted by their upper CP limit prediction. Note that no drug was identified to be effective by the Summation score, i.e., no prioritization was possible and, thus, no plot is shown.

**Figure 6:**
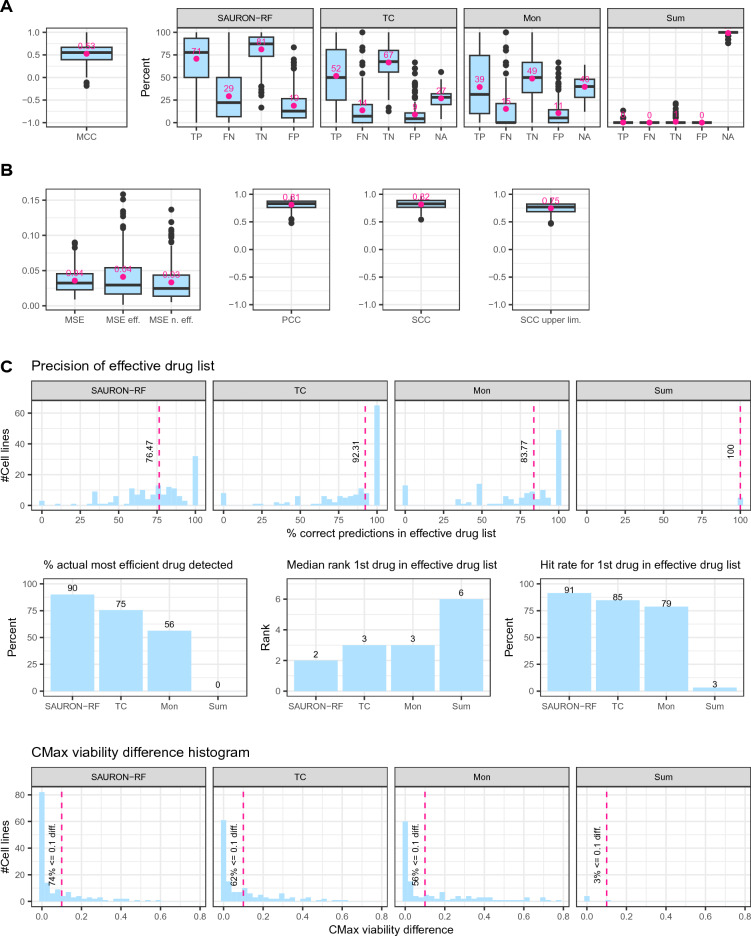
*Prioritization results across all test cell lines of GDSC2.* In A, we show the classification performance of SAURON-RF with and without our CP pipeline. B depicts the regression performance in terms of MSE, PCC and SCC. Here, the MSE is given for the effective drugs, the ineffective drugs, and all drugs. We provide the SCC for the predicted values using SAURON-RF only (SCC) and the upper limit of the CP interval (SCC upper lim.). In C, we plot various measures to evaluate our prioritized drug lists. The upper row of C depicts the precision of SAURON-RF without (SAURON-RF class + SAURON-RF continuous prediction) and with CP (TC + upper limit, Mon + upper limit, Sum + upper limit). In the middle row, we show the percentage of cell lines for which the most efficient drug was detected, the median rank of the first drug in our effective drug list and the percentage of cell lines for which this prediction was a TP. The CMax viability difference between our first drug and the actual first drug is depicted in the lower row.

To achieve a fair performance comparison between the IC50 values and the CMax viabilities, we only considered drugs where both values were available, which resulted in 107 (60 from GDSC1, 47 from GDSC2) potentially analyzable drugs (cf. Supplement 1, Tables 3+4 for more details). Here, one drug data set consists of the following triple: the gene expression matrix, the continuous response of a particular drug, and the discretized response of that drug. We randomly partitioned each data set into a training (70%), calibration (15%), and test (15%) set. The training set was further subdivided to serve as input for a 5-fold cross-validation (CV). Within each CV step, the fold usually employed as test set is partitioned into a disjoint calibration and test set. If the discretized CMax viabilities for one drug contained only one class or consisted of an insufficient number of samples per available class, we discarded this drug for the CMax viability and the corresponding IC50 analyses (see Supplement 1, Figures 3–6 for details). In total, we could thus analyze 41 drugs for the binarized drug responses of GDSC1, 32 drugs for the binarized drug responses of GDSC2, 37 drugs for the ternary drug responses of GDSC1, and 28 drugs for the ternary drug responses of GDSC2. For each data set, the final model is trained on the complete training data, and the CP pipeline is applied accordingly afterwards. Here, we only report the results for the newer GDSC2 data set, which is based on an improved drug sensitivity assay. The results for the GDSC1 data set can be found in Supplement 2.

#### Two classes

**IC50 values:** At first, we applied SAURON-RF without CP to the IC50 data. In Figs. 3 and 4, we show the respective classification and regression performance on the test set. With an average sensitivity of 56%, specificity of 87%, Matthew’s correlation coefficient (MCC) of 0.35, and mean-squared error (MSE) of 2.5 across all drugs, the performance is similar to what we and others observed previously^8,25^. To achieve certainty, we employed our CP pipeline with a fixed allowed error rate of $\alpha = 10\%$. We notice that the certainty guarantee for classification and regression is indeed fulfilled for each of the three investigated classification scores and the regression score, i.e., our sets (classification) and our intervals (regression) contain the actual response with a probability of almost exactly $1-\alpha = 90\%$ on average across all drugs (see Supplement 1, Fig. 9). Next, we analyzed whether this also holds for each class to investigate the effect of class imbalance on the validity (see Supplement 1, Fig. 9). Indeed, we fulfil the marginal coverage property for the majority class (resistant cell lines) for all scores. For the sensitive cell lines (minority class), the Summation score delivers valid sets in all cases, while the True-Class score coverage fluctuates with a mean of approximately 73%. The Mondrian score, which is supposed to fulfil the coverage property for each actual class by definition, exhibits significantly fewer fluctuations than the True-Class score and reaches a coverage of 85% across all drugs. For the Quantile regression score, the coverage for the sensitive cell lines is 86%. Since the adherence to the CP certainty guarantee depends on the number of available data points^24^, the sensitive cell line scarcity can cause these fluctuations.

In our current application scenario, a valid prediction set can either stem from a single class prediction or the set with all classes. To quantify the number of single-class predictions among all predictions, CP efficiency is typically employed. It is defined as the number of single-class predictions divided by the total number of samples. In Fig. 3, we depict the per-drug CP efficiency for the classification scores. We note that the True-Class score with an average CP efficiency of 80% clearly outperforms the Mondrian and Summation scores. The low CP efficiency of the Summation score then directly explains its high coverage: the Summation score almost exclusively predicts two-class sets as output (low efficiency), which by definition must contain the actual class in a binary classification (high coverage). For regression, the CP efficiency is given by the width of the interval. Consequently, it is highly desirable that these intervals are narrow. In Fig. 4, we can, however, see that on average across all drugs, the intervals are relatively large (approximately 50% relative to the spanned training range), which indicates that the trained models need to be refined in that respect. We discuss improvement strategies in the Discussion section.

With CP for classification, we pursue the goal of retaining the true positive and true negative predictions while minimizing the errors, i.e., false positive and false negative predictions. With the fixed $\alpha = 10\%$, the false positive (FP) errors were, on average, reduced from 13 to 9% and the false negative (FN) errors from 44 to 15% for the Mondrian score (cf. Fig. 3). However, the true positive (TP) and true negative (TN) predictions also decrease: from 56 to 48% for the TP and 87 to 60% for the TN. In general, the True-class score also effectively removes FN (from 44 to 25%) and FP (from 13 to 7%). Again, the true predictions are also reduced: from 56 to 43% for the TP and from 87 to 74% for the TN. In contrast, we note that the Summation score does not only almost completely remove the false predictions but also the true predictions, which is in accordance with our previous observations for efficiency. Thus, the True-class score and the Mondrian score clearly outperform the Summation score, while the Mondrian score seems to perform better for the TP and FN values and the True-class score for the TN and FP values.

To showcase the capability of our CP pipeline, we applied it to an adjusted classification version of the multi-task multi-omics deep neural network by Chiu et al.^11^. To render the approach by Chiu et al. amenable to CP, we replaced the activation function of the last layer of the neural network with the sigmoid function, whose outputs can be interpreted as class probabilities (cf. Supplement 1 for details of this analysis). Moreover, we use the binary cross entropy, a dedicated loss function for classification, instead of the mean-squared error. First, we observe the same phenomenon as already demonstrated for various approaches (including neural networks) in the SAURON-RF publication^25^: without specific countermeasures against class or regression imbalance, the minority class (sensitive samples) is predicted poorly (cf. Fig. 27 in Supplement 1). With CP, we achieve the desired 90% certainty (cf. Figures 28–0 in Supplement 1) and consequently remove false predictions (cf. Fig. 27 in Supplement 1). It is particularly noteworthy that the class-wise calibration of the Mondrian score helps to increase the correctly identified sensitive samples (TP) by 20% on average. In total, SAURON-RF with and without CP outperforms our adapted implementation of the approach by Chiu et al.

**CMax viabilities:** Next, we applied SAURON-RF and the CP pipeline to the newly derived CMax viability data set. We find that the CMax viabilities could be predicted with similar sensitivity (64%), specificity (76%), and MCC (0.35) compared to the IC50 data. We again ascertain that CP with a fixed error rate of $\alpha = 10\%$ delivers the desired $90\%$ certainty guarantee on average (cf. Supplement 1, Fig. 10). Indeed, it approximately holds for all three classification scores and the regression score on average across all drugs. For the CMax viabilities, class imbalance also represents an issue. Contrary to the IC50 data, for some drugs, the sensitive cell lines constitute the minority class, and for others, the resistant cell lines do. Still, we discover the same overall trends for the validity of the scores of the minority and majority classes (see Supplement 1, Figs. 11 and 12). Regarding CP efficiency and the reduction in FP and FN predictions, we could also identify similar tendencies compared to our IC50 analyses (see Supplement 1, Figs. 13 and 14). Notably, with an average relative interval size of 0.62, the predicted regression intervals are larger for the CMax analyses than for the IC50 analyses. Overall, the CMax viability could be predicted with similar performance as the established IC50 value. In general, it can be expected that for most ML methods their performance on CMax viabilities will be similar to their performance on IC50 values, since CMax viabilities and IC50 values are highly correlated (cf. Figs. 7 and 8 in Supplement 1).

In our previous publication, we have already demonstrated that SAURON-RF outperforms a variety of approaches^25^. To confirm that this holds when trained on CMax viabilities, we applied an adjusted version of the approach by Chiu et al.^11^ to the CMax viabilities (cf. Supplement 1 for details of this analysis). The overall achieved MSE is similar to that of SAURON-RF (Chiu: 0.09, SAURON:RF: 0.03, cf. Supplement 1, Fig. 31). However, SAURON-RF consistently achieves lower MSEs. Moreover, the correlation results (mean PCC Chiu: 0, cf. Fig. 32 in Supplement 1) imply that the approach by Chiu et al. is not able to sort the cell lines per drug. In contrast, SAURON-RF achieved a decent sorting (mean PCC SAURON-RF: 0.51, cf. Fig. 14 in Supplement 1).

#### Three classes

In the previous section, we described the results for a division of the CMax viability and IC50 values into two classes. However, a more fine-grained division into, e.g. three classes (sensitive, ambiguous, resistant) may more accurately reflect the biological variance and uncertainty of the experimental drug response values and may thus be even more accurately learned and predicted by models.

We first applied SAURON-RF without CP to the ternary CMax drug data sets. The results (see Supplement 1, Figs. 15–17) reflect all general tendencies we reported for the binary partition. Here, it is particularly noteworthy that confusions between the sensitive and resistant classes seem rather rare (9% on average for the sensitive samples and 6% on average for resistant samples), which aligns with the goal of improving certainty. Nevertheless, both classes displayed a high confusion with the ambiguous class (37% on average for the sensitive class and 39% on average for the resistant class), and the average PCC (0.49) and MCC (0.3) are slightly lower than those for the binary partition. We also evaluated the validity and efficiency of the CP pipeline (see Supplement 1, Figs. 15 and 16). Briefly, the efficiency was considerably lower than for the two-class partition. Thus, we decided to focus on the binary partition in the following.

### Cell line-centric analysis: drug prioritization

In the previous sections, we investigated the capabilities of CP in the context of drug sensitivity prediction, and, we conducted drug-centric analyses, i.e., we assessed the model performance on a per-drug basis. In a more realistic application case, the focus is shifted from the drug to the investigated cancer sample, i.e., we are interested in identifying and subsequently prioritizing all suitable drugs for one particular sample. To realistically mimic this application case for one particular cell line, this cell line must be previously unseen by each drug-specific model in the training process. Consequently, all drugs must share the cell lines in the test set, which we ensured for our analyses. In total, we analyzed 25 drugs for GDSC1 and 25 drugs for GDSC2 (cf. Supplement 1Section 4 for a detailed explanation and the respective sizes of the calibration and test set and Tables 3 and 4 for the investigated drugs). Again, we report only the results for the GDSC2 data set here. The respective results for the GDSC1 data set can be found in the Supplement 2.

Due to the shared test set and the across-drug-comparability of the CMax viability, we can now assess the performance from a cell line-centric perspective, i.e., for each cell line, we can identify effective drugs (classification) and then prioritize them (regression). We call a drug effective if its CP class set prediction for a particular cell line consists solely of the single class indicating sensitivity (1). We then subsequently rank all drugs that fulfil this property for a particular cell line using the upper limit of the CP interval. Figure 5 exemplary depicts the results for such a prioritization task for one particular cell line (see Supplement 1, Figs. 23–26 for further cell lines). Notably, the SAURON-RF point predictions (without CP) are not only efficiently distinguishing between effective and non-effective drugs (MCC 0.66) but also sorting them exceptionally well (PCC 0.9). Nevertheless, there still exist FN predictions, which we would like to remove. Both the True-class and the Mondrian score expectedly accomplish this task well at the cost of a few TP predictions. Also, in accordance with our previous drug-centric analyses, the Summation score removes all single-class predictions. In total, the True-class score seems to slightly outperform the Mondrian score, while both are clearly superior to the Summation score. The CP regression intervals are again spanning a wide range of values. Nevertheless, they are ascending alongside the actual values, which indicates that they can be employed for sorting the drugs. A Spearman correlation coefficient (SCC) of 0.87 between the upper limit of the CP interval and the true values confirms this impression. In the lower row of Fig. 5, we also depicted the potential prioritizations obtained by sorting the sets of effective drugs after CP deployment. For the Summation score, no prioritization is possible since no drug was predicted to be effective after CP. However, the rankings introduced by the CP upper limit of the interval are reasonably similar to the actual rankings for the restricted sets of drugs from the Mondrian (SCC 0.6) and True-class (SCC 0.62) scores.

Finally, we analyzed whether these observations hold for all test cell lines (cf. Fig. 6). With an average MCC of 0.53, sensitivity of 71%, specificity of 81%, and PCC of 0.81, SAURON-RF performs well in both the classification and the regression task. The Mondrian and the True-class score effectively remove the false predictions: 48% less FN for Mondrian compared to 53% less FN for True-class as well as 42% less FP for Mondrian and 52% less FP for True-class. However, both scores reduce not only the false predictions but also true predictions: 45% less TP for Mondrian compared to 26% less for True-class as well as 39% less TN for Mondrian compared to 17% for True-class. Indeed, the True-class score does not only reduce the false predictions to a greater extent, but it also preserves more correct predictions, i.e., it clearly outperforms the Mondrian (and the Summation) score in this analysis. For the regression part of the pipeline, we note that the average SCC between the SAURON-RF predictions and the actual values (0.82) is slightly higher than the average SCC between the upper limit of the CP interval and the actual values (0.75). The goal of the prioritization task is to obtain a complete list of potentially effective drugs sorted by their efficiency. We already noticed that the True-class score retains more TP predictions than the Mondrian score, i.e., it yields more complete lists of effective drugs. Furthermore, the effective drug list from the True-class score has a higher median precision (92%) than the Mondrian score (83%). Both are superior to SAURON-RF only (76%). Despite the fact that the TP predictions are also reduced by performing CP, the actual most efficient drug belongs to this list 75% of the time for the True-class score and 56% for the Mondrian score. Moreover, the first drug in our effective drug list has still a median rank of three in the original drug list for both the True-class and the Mondrian score and is a TP prediction in 85% (TC) and 79% (Mon) of cases. The CMax viability difference between this drug and the actual first drug is below 0.1 for 62% of cell lines for the True-class score and 56% of cell lines for the Mondrian score. In relation to the CMax viability range ([0, 1]), this value indicates reasonable proximity of the actual first drug and the drug that we predict to occupy rank one. Overall, we find that the True-class score is most convincing concerning correctness and completeness.

## Discussion

In this paper, we aimed to address two crucial challenges in the area of anti-cancer drug treatment optimization with ML systems: We were interested in (1) reliably predicting anti-cancer drug responses (2) and prioritizing drugs for a given cancer sample based on the reliable predictions.

To tackle the first challenge, we implemented a conformal prediction pipeline providing user-specified certainty levels. Our pipeline can handle not only regression or classification methods but also joint classification and regression methods, which we had recently shown to perform superior to regression or classification alone^25^. We have shown that CP can substantially improve predictions. In particular, CP does not only provide guarantees for predictions, but it successfully diminishes false predictions, i.e., FP and FN, while retaining TP and TN.

To address the second challenge, we developed a novel drug sensitivity measure called CMax viability that is comparable across drugs. Since the CMax viability is based on clinically relevant drug concentrations, it may also help to translate findings into clinical application. By deploying the CP pipeline with our joint regression and classification method SAURON-RF and the CMax viability, we could finally fulfil the prioritization task: We could first use the classification part of our model combined with CP to successfully identify drugs that are very likely effective. In particular, by applying CP, we could eliminate 52% of the remaining 19% ineffective drugs falsely predicted (FP) by SAURON-RF. In total, we thus achieved a median overall 92% precision of our prioritized drug lists, which 75% of the time also contained the most efficient drug. Finally, we could also predict the continuous drug sensitivity and, through the extension of SAURON-RF with quantile regression, build intervals that contain the correct response with a high probability. Our results indicate, that the first drug of our predicted list has a similar CMax viability value as the actual most efficient drug. Thus, the presented CP drug sensitivity prediction and prioritization pipeline can serve as a valuable asset in medical decision support systems.

Nevertheless, we recognize several starting points for improvement. We currently train our models on cell line-based monotherapy responses because of the relatively high abundance of the corresponding data, which is beneficial for training ML models. However, since monotherapy can promote drug resistance^33^, integrating data from drug combination screens would be highly desirable to increase the value of our tool for actual medical decision-making. Here we could leverage information from databases such as DrugComb^33^ or combine our approach with drug synergy prediction methods such as DeepSynergy^34^, MatchMaker^35^, REFLECT^36^, or TreeCombo^37^. Similarly, incorporating data from more complex model systems such as patient-derived xenografts or organoids may be advantageous because they are assumed to more accurately represent tumour characteristics^38^. Apart from that, we were focusing solely on the gene expression data as input features. While gene expression is assumed to be the most informative data type^5^, the interpretability of models can benefit from the integration of additional data types such as mutation and copy number variation data, and, in particular, a priori knowledge, e.g., in the form of known biomarkers^8^, biological pathways and gene interaction networks^12,27^, or drug-based features^39,40^. If those features complement the information from the gene expression data, the performance in terms of certainty of the models might also be increased. Besides, we opted for a particular type of conformal prediction in this work and implemented three different classification scores and one regression score. Since we noted that the regression intervals are rather wide and the prioritization of the effective drug list might be negatively affected by this, it might be beneficial to investigate different regression scores. In addition, there exists a plethora of CP-based techniques^41^, some of which may even further improve classification and regression results. Apart from reliability and interpretability, other important aspects of model trustworthiness are robustness and generalizability, i.e., performance under distribution shifts^3^. We plan to extend our developed frameworks and methods so that they perform well and deliver certainty guarantees under various distribution shifts.

To conclude, we designed and introduced a CP pipeline as a multi-purpose tool for drug sensitivity prediction and prioritization tailored to classification, regression, and simultaneous regression and classification methods. Nevertheless, the pipeline can be applied to various regression or classification models and data sets outside the anti-cancer drug sensitivity prediction and prioritization domain. By routinely investigating certainty guarantees for ML-based decision systems, model weaknesses can be uncovered, and trust in ML may be created.

## Online methods

### Data acquisition

For all our analyses, we employ release 8.3 (June 2020) of the GDSC cancer cell line panel^27^. In particular, we downloaded the pre-processed gene expression values (Affymetrix Human Genome U219 Array), the pre-computed logarithmized IC50 drug responses, and the raw viability data (GDSC1 compounds: Syto60 and resazurin assay, GDSC2 compounds: CellTiter-Glo assay). Additionally, we obtained a list of CMax concentrations from^30^, which represent the peak plasma concentrations of each drug after administration of the highest clinically recommended dose. We combined the CMax concentrations with the raw viability data to derive viabilities at the CMax concentration of each drug and call this measure CMax viability.

### Drug response processing

In our experiments, we use two different drug sensitivity measures, i.e., the logarithmized IC50 value and the CMax viability, separately to fit our models. To achieve a fair performance comparison between the two measures, we restrict our analyses to drugs with availability for both. Thus, we considered 107 drugs from GDSC1 (60) and GDSC2 (47) in total. As a method that simultaneously performs classification and regression, SAURON-RF requires a continuous and discrete drug response vector as input. Therefore, we also derive discretized drug response vectors for both sensitivity measures.

#### IC50 value processing

As a continuous measure of drug sensitivity, we employ the logarithmized IC50 values provided by the GDSC. The corresponding binarized drug response was obtained by applying a custom R-script as described previously^8,25^. The script is based on the binarization procedure introduced by Knijnenburg et al.^7^. For each drug, we thereby derive one binarization threshold that divides the cell lines into sensitive and resistant ones, finally resulting in one binary drug response vector.

#### CMax viability processing

Here, we propose a novel drug sensitivity measure called CMax viability. We define the CMax viability of a drug as the viability at the CMax concentration, which is the peak plasma concentration for the highest clinically recommended drug dose. The CMax viability can take values in the range [0, 1], 0 corresponds to no viability of cancer cells after treatment, and 1 indicates 100% viability. To calculate the viability at the CMax concentration, we first determined the dose-response curves for all cell line-drug combinations with the multilevel mixed effects model by Vis et al.^42^ using the raw drug sensitivity data from the GDSC. For each drug-cell line combination, we then identify the viability at which the corresponding dose-response curve passes through the line parallel to the viability (Y) axis through the CMax concentration of the drug. We call this CMax viability (see Supplement 1, Fig. 1 for examples). The CMax viabilities should be comparable between cell lines and between drugs since they are a measure of the maximal effect that a drug treatment has on a given cell line. In particular, they are independent of the concentration needed for each drug to achieve its maximal effect. Since SAURON-RF demands a discrete and a continuous drug response vector as input, we also discretize the CMax viabilities. In contrast to the IC50 data, we do not derive specific thresholds for each drug. Instead, we leverage the across-drug comparability of the viabilities to determine one threshold (binarization) or even several thresholds (discretizations such as threefold division) applicable to all drugs. To this end, we employ the partitioning around medoids (PAM) clustering algorithm, which has already been used in drug sensitivity prediction to discretize GI50 values^5^. Using PAM on the complete set of available CMax viabilities across all drugs, we identify either two clusters or three clusters of cell lines, which we then interpret to be sensitive and resistant cell lines (in case of two classes) or sensitive, ambiguous, and resistant cell lines (in case of three classes). The mid-points between the clusters are discretization thresholds (cf. Supplement 1, Fig. 2). When we apply the discretization threshold(s) to the continuous CMax viabilities of a particular drug, we obtain a binary (two classes) or ternary (three classes) response vector. In our SAURON-RF analyses, we combine the continuous response vector of one drug either with the binary or the ternary response vector of that drug.

### Extension of SAURON-RF

Our recently published method SAURON-RF represents a possibility to simultaneously perform classification and regression^25^. Similar to HARF by Rahman et al.^9^, it is a random forest-based approach which pursues the strategy to augment regression random forests with class information for the training samples. In particular, SAURON-RF still employs the canonical regression random forest algorithm for model fitting with a continuous response (e.g., IC50 values). However, a binary response vector (e.g., partitioning into sensitive and resistant cell lines) is also used as input. By calculating sample-specific weights or applying upsampling techniques based on the binary response, SAURON-RF can counteract class and regression imbalance. Moreover, SAURON-RF employs the classes to classify (new) samples and to weight the regression predictions of the trees. In this paper, we present two main extensions to SAURON-RF. Firstly, we enable processing more than two classes to allow for a more fine-grained analysis of sensitivity levels. Secondly, we adapt the quantile regression algorithm for random forests described by Meinshausen^29^ to our random forest algorithm. By doing so, we enable the estimation of reliabilities for our predictions and, in particular, the implementation of a combined regression and classification conformal prediction framework. In the following, we first briefly describe the basic SAURON-RF algorithm. Here, we focus on the best-performing versions as determined in^25^. We then discuss the novel extensions in detail.

#### Basic SAURON-RF algorithm

Let $S = \{s_1, \dots , s_N\}$ be the set of samples, $F = \{f_1, \dots , f_P\}$ be the set of features, and ${\textbf {X}} \in {\mathbb {R}}^{N \times P}$ the corresponding model matrix. Suppose ${\textbf {y}} \in {\mathbb {R}}^N$ is the continuous response vector for the training of the weighted regression random forest and ${\textbf {d}} \in \{0,1\}^{N}$ is the corresponding binary response vector derived by comparison of ${\textbf {y}}$ to a threshold *t*. W.l.o.g., let 0 be the majority and 1 the minority class according to ${\textbf {d}}$. Moreover, suppose that $N_{\text {Ma}}$ is the number of samples in the majority class and $N_{\text {Mi}}$ is the number of samples in the minority class. To counteract class imbalance, SAURON-RF relies on sample-specific weights, which can initially be set to

1 $$\begin{aligned} w_{i}^* = {\left\{ \begin{array}{ll} 1, &{}\text {if sample } i \text { belongs to the majority} \\ \frac{N_{\text {Ma}}}{N_{\text {Mi}}}, &{}\text {if sample } i \text { belongs to the minority}\\ \end{array}\right. } \end{aligned}$$

and are then propagated through the training procedure of the random forest. In our previous work, we also proposed the use of alternative weight functions to this *simple* weight function, i.e., weight functions that emphasize samples based on the distance from the threshold *t*, such as

2 $$\begin{aligned} w_i^{*} = \frac{\vert y_i - t\vert ^g}{2 \cdot \sum _{\forall n \in \{1, \dots , N\}: d_n = d_i} \vert y_n - t\vert ^g} \end{aligned}$$

with $g \in \{1,2\}$. Based on the exponent, we name them *linear* and *quadratic*. Given this data, the random forest-based SAURON-RF procedure builds *B* trees as described in the following. At first, we draw a bootstrap sample of size *N* for each $b \in \{1, \dots , B\}$. For each bootstrap sample, a decision tree is then built by repetition of the following steps until some stopping criterion is fulfilled:

- For each current leaf node not yet meeting the stopping criterion, draw $m < P$ features without replacement from the set of features *F*.
- For each drawn feature, find the best splitting point based on the improvement in the used error measure, e.g., the mean squared error (MSE), between the known and predicted response of the samples in that particular node.
- The splitting criterion of the feature with the overall highest improvement in error becomes a new internal node that divides the samples into two groups, which then represent the children of the internal node.

To calculate the prediction of a single tree *b* for a new sample $x \in {\mathbb {R}}^P$, a route from the root to a leaf is traced. The continuous prediction is then the weighted average of the response values in the reached leaf. Let $\mu$ be this particular leaf node and $\delta (\mu )$ be the bootstrap samples that fall into this node. The prediction of tree *b* is given by

3 $$\begin{aligned} {\hat{f}}_b (x) = \sum _{ n \in \delta (\mu )} w_{n}^{\mu } \cdot y_n \quad . \end{aligned}$$

Here, the node-specific sample weight $w_{n}^{\mu }$ is determined from the initial sample weights by applying the formula

4 $$\begin{aligned} w_{n}^{\mu } = \frac{w_n^{*}}{\sum _{i \in \delta (\mu )} w_{i}^{*}}. \end{aligned}$$

Usually, the prediction of a random forest is then obtained by a (weighted) average of the predictions over all trees. For SAURON-RF, we chose to employ tree-specific weights that reflect the data-inherent class distribution. To this end, we add the class assignments to the training samples in the leaf nodes and determine the per-leaf mode of the assignments. As a consequence, each tree can classify a sample; hence, the complete forest can also predict the class of a sample via majority vote over all trees. Based on this class prediction, we then weight a tree *b* as follows: We use the conventional RF weight if a sample is predicted to belong to the forest majority. Otherwise, we employ a tree only if its prediction agrees with RF class prediction. In total, we can express this by the formula

5 $$\begin{aligned} w_{b} (x)= {\left\{ \begin{array}{ll} \frac{1}{B}, &{}\text {if sample } x \text { is predicted to belong to the majority}\\ \frac{I_b(x)}{\sum _{\beta =1}^{B} I_{\beta }(x)}, &{}\text {if sample } x \text { is predicted to belong to the minority}\\ \end{array}\right. }. \end{aligned}$$

Here, the indicator variable $I_b(x)$ is 1 iff tree *b* agrees with the vote of the forest and 0 otherwise. Subsequently, we calculate the total random forest prediction as the weighted average of all trees

6 $$\begin{aligned} {\hat{f}}(x) = \sum _{b = 1}^{B} w_b(x) \cdot {\hat{f}}_b(x) \quad . \end{aligned}$$

#### Multi-class extension

In our previous work, we only considered a binary division into sensitive and resistant cell lines, i.e., we gave definitions for the sample-weight functions of the binary case. However, especially for drug sensitivity prediction, allowing for a more fine-grained class division can be advantageous to more accurately reflect the biological variance and uncertainty of drug response. Thus, we provide straightforward extensions for the Equations 1 and 2.

Let $C = \{c_1, \dots , c_k\}$ be a set of *k* classes. Furthermore, suppose that $N_{c_j}$ with $j \in \{1, \dots , k\}$ is the number of samples of class $c_j$. W.l.o.g., let $c_k$ be the class containing the relative majority (mode) of samples. The *simple* sample weights can be determined by the formula

7 $$\begin{aligned} w_{i}^* = {\left\{ \begin{array}{ll} 1, &{}\text {if sample } i \text { belongs to } c_k \\ \frac{N_{c_k}}{N_{c_j}}, &{}\text {if sample } i \in c_j, \forall j \in \{1, \dots , k-1\}\\ \end{array}\right. } \end{aligned}$$

To define the *linear* and *quadratic* weight function for the multi-class setting, we additionally assume that the classes are ordered in ascending order of the thresholds that divide the corresponding class pairs. To this end, let $t_{j,r} \in \{t_{1,2}, \dots , t_{k-1,k}\}$ be the threshold that divides the samples from class *j* and *r*. The weight function in Equation 2 remains unaltered for samples belonging to class $c_1$ and $c_k$ since these classes have only one neighbouring threshold. For all other samples, the distances from the two thresholds are averaged. In total, the following formula provides the sample weights

8 $$\begin{aligned} w_{i}^* = {\left\{ \begin{array}{ll} \frac{\vert y_i - t_{1,2}\vert ^g}{k \cdot \sum _{\forall n \in \{1, \dots , N\}: d_n = d_i} \vert y_n - t_{1,2}\vert ^g}, \text {if sample } i \text { belongs to } c_1 \\ \frac{\vert y_i - t_{k-1,k}\vert ^g}{k \cdot \sum _{\forall n \in \{1, \dots , N\}: d_n = d_i} \vert y_n - t_{k-1,k}\vert ^g}, \text {if sample } i \text { belongs to } c_k \\ \frac{\vert y_i - t_{j-1,j}\vert ^g + \vert y_i - t_{j,j+1}\vert ^g}{k \cdot \sum _{\forall n \in \{1, \dots , N\}: d_n = d_i} \vert y_n - t_{j-1,j}\vert ^g + \vert y_n - t_{j,j+1}\vert ^g}, \text { otherwise }\\ \end{array}\right. } \end{aligned}$$

#### Quantile regression for SAURON-RF

Statistical learning algorithms aim to to express the relationship between a predictor variable, e.g., in our case, the *p*-dimensional random variable *X*, and the real-valued response variable *Y*, such that the resulting model approximates *Y* with minimal error. To this end, standard regression algorithms often employ a squared-error loss function with which the conditional mean $E(Y \vert X=x)$ is estimated^29^. Random forests also approximate the conditional mean^29^. However, there exist cases in which not only the conditional mean but the complete conditional distribution $F(y \vert X=x)$ is of interest, e.g., outlier detection or reliability estimation^29,43^. In our application case, for example, it might be of interest to obtain a drug response value for a specific cell line that is not surpassed with high probability or to estimate the dispersion of response values to assess the reliability with which the drug response of that specific cell line can be predicted. Quantile regression has been developed to address such questions^44^. In particular, Meinshausen proposed quantile regression forests, a generalisation to random forests, as a possibility to infer conditional quantiles. This algorithm estimates the conditional distribution function $F(y \vert X = x)$. In the next sections, we provide an adjusted quantile regression algorithm for SAURON-RF. Thereby, we can, later on, define conformal prediction for our method, which ultimately even delivers guarantees for the reliability of the prediction.

Let the conditional distribution function $F(y\vert X=x)$ be defined by the probability that *Y* is at most *y* for *X* equal to *x*, i.e.,

9 $$\begin{aligned} F(y\vert X=x) = P(Y \le y \vert X=x). \end{aligned}$$

The $\alpha$-quantile for $X = x$ is then defined as the minimum *y* for which the conditional distribution function is at least $\alpha$:

10 $$\begin{aligned} Q_{\alpha }(x) = \inf \{y: F(y\vert X=x) \ge \alpha \} \end{aligned}$$

Hence, we need an estimate of the conditional distribution function to perform quantile regression. Meinshausen shows that this is indeed possible with random forests by interpreting them as proposed by Lin and Jeon, which view them as an adaptive neighbourhood classification or regression algorithm^45^. In particular, Meinshausen employs the fact that the final prediction of an ordinary random forest is an estimate of the conditional mean and that it can be viewed as a weighted sum of the response values of the training observations. To this end, let ${\textbf {y}} \in {\mathbb {R}}^{N}$ be the response vector as defined in Section "Basic SAURON-RF algorithm". Then, the final prediction of the ordinary RF can be expressed as

11 $$\begin{aligned} E(Y \vert X=x ) = {\hat{f}}(x) = \sum _{i=1}^{N} w_i(x) \cdot y_i \end{aligned}$$

with $w_i(x)$ representing a forest-wide weight for each training sample $i \in \{1, \dots , N\}$ (see^29^ for definition in usual random forests). In contrast, we calculated the final prediction of SAURON-RF as a weighted average of the trees (cf. Equation 6). The equivalence of Equation 6 and 11 for SAURON-RF, can however also be established:

$$\begin{aligned} \sum _{b = 1}^{B} w_b(x) \cdot {\hat{f}}_b(x)= & {} \sum _{b=1}^{B} w_b(x) \cdot \sum _{ n \in \delta (\mu _b)} w_{n}^{\mu _b} \cdot y_n\\= & {} \sum _{b=1}^{B} w_b(x) \cdot \sum _{ i=1}^{N} I_{\delta (\mu _b)} (i) \cdot w_{i^*}^{\mu _b} \cdot y_i\\= & {} \sum _{b=1}^{B} \sum _{ i=1}^{N} w_b(x) \cdot I_{\delta (\mu _b)} (i) \cdot w_{i^*}^{\mu _b} \cdot y_i\\= & {} \sum _{i=1}^{N} \sum _{ b=1}^{B} w_b(x) \cdot I_{\delta (\mu _b)} (i) \cdot w_{i^*}^{\mu _b} \cdot y_i\\= & {} \sum _{i=1}^{N} w_{i}(x) \cdot y_i \end{aligned}$$

where $w_i(x) = \sum _{b=1}^{B} w_b(x) \cdot I_{\delta (\mu _b)}(i) \cdot w_{i^*}^{\mu _b}$ and $I_{\delta (\mu _b)}(i)$ is equal to 1 iff sample i is in leaf node $\mu _b$ and 0 otherwise. Note that $w_{i^*}^{\mu _b}$ refers to an actual sample from the original data set instead of a bootstrap sample for a specific tree, i.e., its definition slightly differs from the one introduced in Equation 4 and is given as

12 $$\begin{aligned} w_{i^*}^{\mu _b} = \sum _{i^{'} \in \delta (\mu _b): i^{'} = i} w_{i^{'}}^{\mu _b}. \end{aligned}$$

Given this equivalence, we can - in analogy to Meinshausen - estimate the conditional distribution function by

13 $$\begin{aligned} {\hat{F}}(y \vert X=x) = \sum _{i=1}^{N} w_i(x) \cdot I_{y_i \le y} \end{aligned}$$

with $I_{y_i \le y}$ being 1 iff $y_i \le y$ and 0 otherwise.

Finally, the quantile regression forest algorithm for SAURON-RF reads as follows

1. Train the SAURON-RF regression random forest as explained in 4.3 and 4.3.
2. For a new sample *x*, trace a route from root to leaf for each tree $b \in \{1, \dots , B\}$, which results in the set of reached leaf nodes $L = \{\mu _1, \dots , \mu _B\}$.
3. For each $\mu _b \in L$ , calculate the node-specific sample weights (cf. Equations 4 and 12) of all training samples $x_i, i \in \{1, \dots , N\}$.
4. Then, average these weights across *L* to obtain a forest-wide weight of each training sample $i \in \{1, \cdots , N\}$, i.e.,
  14 $$\begin{aligned} w_i(x) = \sum _{b =1}^{B} w_b(x) \cdot I_{\delta (\mu _b)}(i) \cdot w_{i^*}^{\mu _b}, \forall i \in \{1, \dots , N\} \end{aligned}$$
5. Now, an estimate of the distribution function ${\hat{F}}(y \vert X=x)$ can be determined for all $y \in {\mathbb {R}}$ by using Equation 13.
6. By plugging ${\hat{F}}(y \vert X=x)$ into Equation 10, calculate the estimate of the conditional quantile ${\hat{Q}}_{\alpha }(x)$, i.e., return the minimal response value *y* for which the estimate of the conditional distribution function ${\hat{F}}(y \vert X=x)$ is at least $\alpha$.

### Conformal prediction

One critical challenge of ML in healthcare is creating trust in the generated models and their predictions. To this end, the predictions delivered by the models can readily be employed to assess the overall model performance in terms of conventional error measures as long as the true response is known, i.e., during training, validation, and testing. However, an estimation for the reliability of the prediction itself is usually not provided, which means that we cannot tell if the predictions for new samples with unknown responses will likely be close to their true but unknown values. Conformal prediction (CP) is a reliability estimation framework that can sit on top of a variety of ML methods given that they provide a notion of (un)certainty for their predictions^24^. For random forest classifiers, such a notion of certainty can be represented by the proportion of trees that voted for the predicted class. For random forest regressors, quantile regression may be used. For a user-specified maximal allowed error rate $\alpha$, CP converts this notion of (un)certainty into a mathematical rigorous certainty guarantee: it constructs a so-called valid prediction set (classification) or interval (regression), which then contains the true value with a certainty of almost exactly $1- \alpha$.

In the following sections, we first introduce the conformal prediction procedure that employs a notion of (un)certainty in a score function to convert it into a rigorous (un)certainty guarantee by delivering valid prediction sets and intervals. After describing the conformal prediction algorithm, we present the score functions, we evaluated throughout this paper.

#### Conformal prediction procedure

Training supervised ML models generally includes partitioning the complete data set into a disjoint training and test data set. While the training data set usually serves for the training of the parameters of a particular ML model, the test set is used to evaluate the performance of this model on data previously unseen by the model. Conformal prediction needs a third disjoint data set, the so-called calibration data set employed to calculate statistics on the (un)certainty of the model. For our application case, let $Z = ({\textbf {X}}, {\textbf {y}}, {\textbf {d}})$ be the complete data set with ${\textbf {X}}$, ${\textbf {y}}$, and ${\textbf {d}}$ being defined as introduced in Section "Basic SAURON-RF algorithm". Let $Z_{\text {train}}$, $Z_{\text {cal}}$, and $Z_{\text {test}}$ be the corresponding training, calibration, and test set, respectively. Moreover, let $N_{\text {train}}$, $N_{\text {test}}$, and $N_{\text {cal}}$ denote the number of samples in each of these data sets, and let $\alpha \in [0,1]$ be the desired maximal error rate of the user. Then, CP can be divided into the ensuing four steps^24^

1. Train the chosen ML models using $Z_{\text {train}}$.
2. Define a score function *s*(*x*, *d*) (classification) or *s*(*x*, *y*) (regression) that is based on the given notion of (un)certainty by the model.
3. Apply the trained model to $Z_{\text {cal}}$ and calculate one score for each calibration sample. Based on the resulting score distribution, derive a threshold ${\hat{q}}$ that corresponds to the allowed error rate $\alpha$.
4. Calculate the corresponding scores for $Z_{\text {test}}$ and use ${\hat{q}}$ to form intervals (regression) or sets (classification).

By performing CP as outlined above, we construct intervals or sets that contain the true response with a probability of almost exactly $1-\alpha$, which are also called valid prediction intervals or sets. In particular, let ${\mathcal {C}}(x_{i})$ represent this interval or set for $x_i \in {\textbf {X}}_{\text {test}}$, using CP it is guaranteed that

15 $$\begin{aligned} 1 - \alpha \le P(d_{i} \in {\mathcal {C}}(x_{i})) \le 1- \alpha + \frac{1}{N_{\text {cal}} +1} \end{aligned}$$

for classification. For regression, the same holds with $d_i$ replaced by the respective continuous response $y_i$. Hence, it holds that the more calibration samples are available, the lower the upper boundary becomes, i.e. the certainty (also called coverage) would become exactly $1- \alpha$ for $N_{\text {cal}} \rightarrow \infty$. Indeed, the relationship between $N_{\text {cal}}$ and the observed coverage can be described analytically. We refer to^24,46^ for in-depth information on this issue.

Equation 15 is also called the marginal coverage property of CP since the certainty is averaged (marginalized) over the randomness in the test and calibration data points^24^. However, we would usually like to guarantee conditional coverage, which means that we can guarantee the coverage for a particular sample, i.e., we would like to guarantee

16 $$\begin{aligned} P(d_i \in {\mathcal {C}}(x_i) \vert x_i) \ge 1- \alpha \end{aligned}$$

for classification ($d_i$ replaced by $y_i$ for regression). While it is impossible to achieve conditional coverage with CP in all possible scenarios according to Vovk^46^, it can be approximated with appropriate scores^24^. Therefore, we also assessed our models and score functions in that respect.

#### Classification scores

As mentioned above, CP consists of four steps. In particular, step two requires defining a score function based on the notion of (un)certainty given by the model. The choice of score function heavily influences the quality of results^24^. Angelopoulos and Bates^24^ thoroughly discuss a variety of criteria that can play a role in selecting the best score function for different application cases. In the following, we will briefly describe the score functions evaluated in this manuscript.

**True-class (TC) score:** Arguably, the most simple scoring function that Angelopoulos and Bates depict represents the probability of misclassifying a sample. Given a sample $x_j$ from the calibration data set, it is defined as

17 $$\begin{aligned} s_{\text {TC}}(x_j, d_j) = 1- P(d_j \vert x_j) \quad . \end{aligned}$$

For a random forest, $P(d_j \vert x_j)$ is the proportion of trees that voted for the true class $d_j$ of the calibration sample *j*. The True-class score results in high values if the true class of sample *j* had a low probability and vice versa. As described in Step 3 of the CP procedure, we calculate this score for each sample in $Z_{\text {cal}}$ resulting in a score distribution. Based on this distribution, we derive the threshold ${\hat{q}}$ that tells us which classes to add to our prediction set to fulfil the marginal coverage property in Equation 15. In particular, we calculate ${\hat{q}}$ as a modified (1-$\alpha$)-quantile of the distribution. We must modify the usual $1-\alpha$ quantile to account for the finite number of calibration samples $N_{\text {cal}}$. Thus, we determine ${\hat{q}}$ as the $\frac{\lceil (N_{\text {cal}}+1)(1-\alpha )\rceil }{N_{\text {cal}}}$ quantile. For a new sample $x_i$, we do not know the true class. Hence, we calculate the score for all classes and add those with a score smaller or equal to ${\hat{q}}$ to the prediction set, i.e.,

18 $$\begin{aligned} {\mathcal {C}}(x_i) = \{c_l \vert s_{\text {TC}}(x_i, c_l) \le {\hat{q}} , \forall l \in \{1, \cdots , k\}\} \end{aligned}$$

**Summation (Sum) score:** Angelopoulos and Bates propose another score function based on ideas from^47,48^. We call this score function Summation score since it builds on the concept of including all classes until their sum reaches a particular value. Given a sample $x_j$ from the calibration data set, the score is calculated as follows

1. Firstly, sort the model probabilities for all classes $c_l \in \{1, \cdots , k\}$ decreasingly from highest to lowest probability. For random forest classifiers, this probability is again given by the proportions of samples that voted for a class. W.l.o.g., let $u_j = [c_1, \cdots , c_k]$ be this sorted list for sample $x_j$.
2. Secondly, add up the probabilities of all classes in this sorted list until the true class $d_j$ of sample $x_j$ is reached:
  19 $$\begin{aligned} s_{\text {Sum}}(x_j, d_j) = \sum _{o= c_1}^{d_j} P(o \vert x_j) \end{aligned}$$

If the Summation score results in high values, it was either because the correct class was predicted with high probability or the sample was misclassified. If the score is comparably low, the correct class was predicted with a low probability. We calculate the score for each sample in $Z_{\text {cal}}$ to obtain the score distribution. To subsequently derive the threshold ${\hat{q}}$ that we need to decide whether to include a class into our prediction set for a new sample, we again calculate the adjusted $1-\alpha$ quantile ${\hat{q}}$ as described in the previous section. For a new sample $x_i$, we then determine the prediction set ${\mathcal {C}}(x_i)$ by performing the two steps above. At first, we sort the predicted class probabilities from highest to lowest. Again, w.l.o.g. let $u_i = [c_1, \cdots , c_k]$ be this sorted list for sample *i*. We obtain ${\mathcal {C}}(x_i)$ by adding all classes until, in sum, their predicted class probabilities exceed ${\hat{q}}$

20 $$\begin{aligned} {\mathcal {C}}(x_i) = \{c_l\ \vert l \in \{1, \ldots , sup \{l' : \sum _{u= 1}^{l'} P(c_u \vert x_i) < {\hat{q}}\} +1 \} \} \end{aligned}$$

**Mondrian (Mon) score:** The Mondrian score, as typically used in drug discovery^18^, is a type of class-conditional CP in which the marginal coverage from Equation 15 is extended to hold for each available class, i.e., the predicted sets for a new sample $x_i$ from the test set should fulfil^24,49^

21 $$\begin{aligned} P(d_i \in {\mathcal {C}}(x_i) \vert d_i = c_l) \ge 1- \alpha , \forall l \in \{1, \ldots , k\} . \end{aligned}$$

The general idea of Mondrian CP is to perform the calibration step in each class separately. Mondrian CP using the True-class score can be conducted as follows: we calculate $s_{\text {TC}}$ for each sample from the calibration data set and divide the resulting distribution into *k* sub-distributions, one distribution for each class. We then determine the modified $1-\alpha$ quantile for each distribution, resulting in *k* thresholds ${\hat{q}}^{l}, l \in \{1, \ldots , k\}$. For a new sample *i*, we add a class $c_l$ to the prediction set ${\mathcal {C}}(x_i)$ if it fulfills $s_{\text {TC}}(x_i, c_l) \le {\hat{q}}^{l}$.

#### Regression score

As outlined in Section "Conformal prediction procedure", regardless of whether classification or regression is performed, the CP procedure can be applied as long as an appropriate score function is provided. Romano et al. developed a CP method based on quantile regression^50^. In principle, we can already employ quantile regression itself to provide an estimate of the certainty of the regression: we can train one model ${\hat{f}}_{\frac{\alpha }{2}}$ that predicts the $\frac{\alpha }{2}$-quantile and another model ${\hat{f}}_{1- \frac{\alpha }{2}}$ that predicts the $1-\frac{\alpha }{2}$-quantile and expect the interval $[{\hat{f}}_{\frac{\alpha }{2}}, {\hat{f}}_{1- \frac{\alpha }{2}}]$ to contain the true response with $1-\alpha$ certainty. However, we do not know how accurate the predicted intervals are because they were calculated on the training data set. Thus, Romano et al. define a score function, which we also call Quantile (Qu) in the following, that quantifies whether the samples from the calibration data set were within the signified quantile interval as often as to be expected. For a sample $x_j$ from the calibration data set with its known response $y_j$, this score function represents the signed distance between $y_j$ and the nearest interval boundary

22 $$\begin{aligned} s_{\text {Qu}}(x_j, y_j) = max {\left\{ \begin{array}{ll} {\hat{f}}_{\frac{\alpha }{2}}(x_j) - y_j\\ y_j - {\hat{f}}_{1- \frac{\alpha }{2}}(x_j)\\ \end{array}\right. } \end{aligned}$$

The sign of this score function is positive if $y_j$ is outside of the interval and negative if $y_j$ is within the interval. Again, we calculate $s_{\text {Qu}}$ for each sample of the calibration data set and receive a score distribution on which we determine ${\hat{q}}$ as the modified $1-\alpha$ quantile of the distribution. If the quantile regression achieves the desired coverage, ${\hat{q}}$ will be approximately 0, and the predicted interval for a new sample $x_i$ will remain unaltered. Otherwise, the interval will be widened (${\hat{q}} > 0$) or narrowed (${\hat{q}} <0$), i.e., the predicted interval is

23 $$\begin{aligned} {\mathcal {C}}(x_i) = [{\hat{f}}_{\frac{\alpha }{2}}(x_i) - {\hat{q}}, {\hat{f}}_{1- \frac{\alpha }{2}}(x_i) + {\hat{q}}] \end{aligned}$$

### Supplementary Information

Supplementary Information 1.

Supplementary Information 2.

## Data Availability

The drug data sets can be downloaded from the publicly available repository of the Genomics of Drug Sensitivity in Cancer (GDSC) database. All other material is made publicly available via the Supplementary Information of this paper or the corresponding github page: https://github.com/unisb-bioinf/Conformal-Drug-Sensitivity-Prediction.git.

## Code availability

The code is deposited in the following publicly available github: https://github.com/unisb-bioinf/Conformal-Drug-Sensitivity-Prediction.git.

## References

[1] Rafique R, Islam SR, Kazi JU (2021). Machine learning in the prediction of cancer therapy. Comput. Struct. Biotechnol. J..

[2] Adam G, Rampášek L, Safikhani Z, Smirnov P, Haibe-Kains B, Goldenberg A (2020). Machine learning approaches to drug response prediction: Challenges and recent progress. NPJ Precis. Oncol..

[3] Sharifi-Noghabi H, Jahangiri-Tazehkand S, Smirnov P, Hon C, Mammoliti A, Nair SK, Mer AS, Ester M, Haibe-Kains B (2021). Drug sensitivity prediction from cell line-based pharmacogenomics data: Guidelines for developing machine learning models. Brief. Bioinform..

[4] Shen B, Feng F, Li K, Lin P, Ma L, Li H (2023). A systematic assessment of deep learning methods for drug response prediction: from in vitro to clinical applications. Brief. Bioinform..

[5] Costello JC, Heiser LM, Georgii E, Gönen M, Menden MP, Wang NJ, Bansal M, Ammad-Ud-Din M, Hintsanen P, Khan SA (2014). A community effort to assess and improve drug sensitivity prediction algorithms. Nat. Biotechnol..

[6] Masica DL, Karchin R (2013). Collections of simultaneously altered genes as biomarkers of cancer cell drug responsemultigene biomarkers of cancer cell drug response. Can. Res..

[7] Knijnenburg TA, Klau GW, Iorio F, Garnett MJ, McDermott U, Shmulevich I, Wessels LF (2016). Logic models to predict continuous outputs based on binary inputs with an application to personalized cancer therapy. Sci. Rep..

[8] Lenhof K, Gerstner N, Kehl T, Eckhart L, Schneider L, Lenhof H-P (2021). Merida: A novel boolean logic-based integer linear program for personalized cancer therapy. Bioinformatics.

[9] Rahman R, Matlock K, Ghosh S, Pal R (2017). Heterogeneity aware random forest for drug sensitivity prediction. Sci. Rep..

[10] Matlock K, De Niz C, Rahman R, Ghosh S, Pal R (2018). Investigation of model stacking for drug sensitivity prediction. BMC Bioinform..

[11] Chiu Y-C, Chen H-IH, Zhang T, Zhang S, Gorthi A, Wang L-J, Huang Y, Chen Y (2019). Predicting drug response of tumors from integrated genomic profiles by deep neural networks. BMC Med. Genom..

[12] Oskooei A, Manica M, Mathis R, Martínez MR (2019). Network-based biased tree ensembles (netbite) for drug sensitivity prediction and drug sensitivity biomarker identification in cancer. Sci. Rep..

[13] Guan N-N, Zhao Y, Wang C-C, Li J-Q, Chen X, Piao X (2019). Anticancer drug response prediction in cell lines using weighted graph regularized matrix factorization. Mol. Therapy-nucleic Acids.

[14] Nicora G, Rios M, Abu-Hanna A, Bellazzi R (2022). Evaluating pointwise reliability of machine learning prediction. J. Biomed. Inform..

[15] Štrumbelj E, Bosnić Z, Kononenko I, Zakotnik B, Grašič Kuhar C (2010). Explanation and reliability of prediction models: The case of breast cancer recurrence. Knowl. Inf. Syst..

[16] Burkart N, Huber MF (2021). A survey on the explainability of supervised machine learning. J. Artif. Intell. Res..

[17] Fang Y, Xu P, Yang J, Qin Y (2018). A quantile regression forest based method to predict drug response and assess prediction reliability. PLoS ONE.

[18] Norinder U, Carlsson L, Boyer S, Eklund M (2014). Introducing conformal prediction in predictive modeling: A transparent and flexible alternative to applicability domain determination. J. Chem. Inf. Model..

[19] Alvarsson J, McShane SA, Norinder U, Spjuth O (2021). Predicting with confidence: using conformal prediction in drug discovery. J. Pharm. Sci..

[20] Morger A, Mathea M, Achenbach JH, Wolf A, Buesen R, Schleifer K-J, Landsiedel R, Volkamer A (2020). Knowtox: Pipeline and case study for confident prediction of potential toxic effects of compounds in early phases of development. J. Cheminform..

[21] Morger A, Svensson F, Arvidsson McShane S, Gauraha N, Norinder U, Spjuth O, Volkamer A (2021). Assessing the calibration in toxicological in vitro models with conformal prediction. J. Cheminform..

[22] Gammerman, A., Vovk, V. & Vapnik, V. Learning by transduction. In: Proceedings of the Fourteenth Conference on Uncertainty in Artificial Intelligence. UAI’98, pp. 148–155. Morgan Kaufmann Publishers Inc., San Francisco, CA, USA (1998)

[23] Vovk V, Gammerman A, Shafer G (2005). Algorithmic Learning in a Random World.

[24] Angelopoulos, A.N. & Bates, S. A gentle introduction to conformal prediction and distribution-free uncertainty quantification. arXiv preprint arXiv:2107.07511 (2021)

[25] Lenhof K, Eckhart L, Gerstner N, Kehl T, Lenhof H-P (2022). Simultaneous regression and classification for drug sensitivity prediction using an advanced random forest method. Sci. Rep..

[26] Yang W, Soares J, Greninger P, Edelman EJ, Lightfoot H, Forbes S, Bindal N, Beare D, Smith JA, Thompson IR (2012). Genomics of drug sensitivity in cancer (gdsc): A resource for therapeutic biomarker discovery in cancer cells. Nucleic Acids Res..

[27] Iorio F, Knijnenburg TA, Vis DJ, Bignell GR, Menden MP, Schubert M, Aben N, Gonçalves E, Barthorpe S, Lightfoot H (2016). A landscape of pharmacogenomic interactions in cancer. Cell.

[28] Consortium, C.C.L.E. of Drug Sensitivity in Cancer Consortium, G., *et al.*: Pharmacogenomic agreement between two cancer cell line data sets. *Nature*. **528**(7580), 84–87 (2015) 10.1038/nature15736 PMC6343827 26570998

[29] Meinshausen N, Ridgeway G (2006). Quantile regression forests. J. Mach. Learn. Res..

[30] Liston DR, Davis M (2017). Clinically relevant concentrations of anticancer drugs: A guide for nonclinical studiesguide to clinical exposures of anticancer drugs. Clin. Cancer Res..

[31] He X, Folkman L, Borgwardt K (2018). Kernelized rank learning for personalized drug recommendation. Bioinformatics.

[32] Liu M, Shen X, Pan W (2022). Deep reinforcement learning for personalized treatment recommendation. Stat. Med..

[33] Zagidullin B, Aldahdooh J, Zheng S, Wang W, Wang Y, Saad J, Malyutina A, Jafari M, Tanoli Z, Pessia A (2019). Drugcomb: An integrative cancer drug combination data portal. Nucleic Acids Res..

[34] Preuer K, Lewis RP, Hochreiter S, Bender A, Bulusu KC, Klambauer G (2018). Deepsynergy: Predicting anti-cancer drug synergy with deep learning. Bioinformatics.

[35] Kuru HI, Tastan O, Cicek AE (2021). Matchmaker: A deep learning framework for drug synergy prediction. IEEE/ACM Trans. Comput. Biol. Bioinf..

[36] Li X, Dowling EK, Yan G, Dereli Z, Bozorgui B, Imarinad P, Elnaggar JH, Luna A, Menter DG, Pilie PG, Yap TA, Kopetz S, Sander C, Korkut A (2022). Precision combination therapies based on recurrent oncogenic co-alterations. Cancer Discov..

[37] Janizek, J.D., Celik, S. & Lee, S.-I. Explainable machine learning prediction of synergistic drug combinations for precision cancer medicine. BioRxiv, 331769 (2018)

[38] Gao H, Korn JM, Ferretti S, Monahan JE, Wang Y, Singh M, Zhang C, Schnell C, Yang G, Zhang Y (2015). High-throughput screening using patient-derived tumor xenografts to predict clinical trial drug response. Nat. Med..

[39] Menden MP, Iorio F, Garnett M, McDermott U, Benes CH, Ballester PJ, Saez-Rodriguez J (2013). Machine learning prediction of cancer cell sensitivity to drugs based on genomic and chemical properties. PLoS ONE.

[40] Zhang N, Wang H, Fang Y, Wang J, Zheng X, Liu XS (2015). Predicting anticancer drug responses using a dual-layer integrated cell line-drug network model. PLoS Comput. Biol..

[41] Vazquez J, Facelli JC (2022). Conformal prediction in clinical medical sciences. J. Healthcare Inf. Res..

[42] Vis DJ, Bombardelli L, Lightfoot H, Iorio F, Garnett MJ, Wessels LF (2016). Multilevel models improve precision and speed of ic50 estimates. Pharmacogenomics.

[43] Takeuchi, I., Le, Q., Sears, T., Smola, A., et al. Nonparametric quantile estimation (2006)

[44] Koenker R (2005). Quantile Regression.

[45] Lin, Y. & Jeon, Y. Random forests and adaptive nearest neighbors. Technical Report **1055** (2002)

[46] Vovk, V. Conditional validity of inductive conformal predictors. In: Asian Conference on Machine Learning, pp. 475–490 (2012). PMLR

[47] Romano Y, Sesia M, Candes E (2020). Classification with valid and adaptive coverage. Adv. Neural. Inf. Process. Syst..

[48] Angelopoulos, A., Bates, S., Malik, J. & Jordan, M.I. Uncertainty sets for image classifiers using conformal prediction. arXiv preprint arXiv:2009.14193 (2020)

[49] Fontana M, Zeni G, Vantini S (2023). Conformal prediction: A unified review of theory and new challenges. Bernoulli.

[50] Romano, Y., Patterson, E. & Candes, E. Conformalized quantile regression. *Advances in neural information processing systems*. **32** (2019)

