# Supplementary material for: Reliable anti-cancer drug sensitivity prediction and prioritization
Source: Sci Rep. 2024 May 29;14:12303. doi: 10.1038/s41598-024-62956-6 (PMC11137046; doi:10.1038/s41598-024-62956-6)
Supplement: Supplementary file 1 — Supplementary Information 1. [file 41598_2024_62956_MOESM1_ESM.pdf]

# - Supplement 1 - Reliable Anti-Cancer Drug Sensitivity Prediction and Prioritization

Kerstin Lenhof, Lea Eckhart, Lisa-Marie Rolli, Andrea Volkamer, and Hans-Peter Lenhof

1 Literature review

Table 1: **Comparison between different tools for drug sensitivity prediction with respect to certainty estimation and prioritization.**
*Rahman et al. use the jackknife after the bootstrap approach but did not explicitly employ it to deliver reliable predictions
**He et al. and Liu et al. perform drug recommendation (cf. main manuscript for definition), which is similar but not identical to prioritization

Name and author	Methodology	Drug sensitivity measure	Certainty estimation	Prioritization
Menden et al. [5]	neural network	IC50	✗	✗
Zhang et al. [6]	dual-layer integrated drug-cell line similarity network	activity area, IC50	✗	✗
LOBICO by Knijnenburg et al. [7]	integer linear program delivering Boolean rules	binarized IC50	✗	✗
Stanfield et al. [8]	cell line and drug proximity networks	binarized IC50	✗	✗
SRMF by Wang et al. [9]	similarity regularized matrix factorization	IC50, activity area	✗	✗
HARF by Rahman et al. [10]	random forest augmented with cancer types	AUC	(✗)*	✗
HNMDRP by Zhang et al. [11]	similarity networks	IC50	✗	✗
Matlock et al. [12]	model stacking	AUC	✗	✗
				Continued on next page

Table 1 – continued from previous page

Name and author	Methodology	Drug sens. meas.	Certainty est.	Prio.
KRL by He et al. [13]	kernelized rank learning	normalized IC50	✗	(✓)**
RWEN by Basu et al. [14]	response-weighted elastic net	AUC	✗	✗
CDRscan by Chang et al. [15]	convolutional neural networks	IC50	✗	✗
QRF by Fang et al. [16]	quantile regression random forest	activity area	(✓)	✗
NCFGER by Liu et al. [17]	neighbor-based collaborative filtering with global effect removal	IC50	✗	✗
DeepDR by Chiu et al. [18]	neural networks	IC50	✗	✗
Deep-Resp-Forest by Su et al. [19]	deep cascaded forest	binarized activity area, binarized IC50	✗	✗
Dr.VAE by Rampášek et al. [20]	semi-supervised generative modeling based on variational autoencoders	binarized area above the dose-response curve	✗	✗
netBITE by Oskooei et al. [21]	biased tree ensemble	IC50	✗	✗
Deng et al. [22]	neural network	normalized AUC	✗	✗
ADRML by Ahmadi Moughari and Eslahchi [29]	manifold learning	IC50	✗	✗
PathDSP by Tang and Gottlieb [23]	neural network	IC50	✗	✗
MERIDA by Lenhof et al. [24]	integer linear program delivering Boolean rules	binarized IC50	✗	✗
Meybodi and Eslahchi [30]	elastic net	IC50	✗	✗
GraphDRP by Nguyen et al. [25]	neural network	IC50	✗	✗
PPORank by Liu et al. [26]	deep reinforcement learning	normalized IC50	✗	(✓)**

Continued on next page

Table 1 – continued from previous page

Name and author	Methodology	Drug sens. meas.	Certainty est.	Prio.
SAURON-RF by Lenhof et al. [27]	simultaneous regression and classification random forest	IC50 and binarized IC50 simultaneously	✗	✗
reliable SAURON-RF by Lenhof and Eckhart et al. as suggested in this manuscript	simultaneous regression and classification random forest using CP	IC50 and binarized IC50 simultaneously, CMax viability and binarized CMax viability simultaneously	✓	✓

2 Fitting of dose-response curves and calculation of CMax viability

In order to calculate our proposed measure of drug response, the CMax viability, we fitted dose-response curves for each cell line-drug combination in the GDSC database using the R package gdscIC50 by [1]. The package employs a multilevel mixed effects model by [2] that fits dose-response curves as two-parameter sigmoidal functions. Here, one parameter controls the slope of the curve and the other controls the position of the inflection point. While the slope parameter is constant for one cell line across all drugs, the position of the inflection point is tuned for each cell line-drug pair separately.

We applied the curve-fitting procedure to the raw measurements of the GDSC1 and GDSC2 data sets separately. We then discarded all cell line-drug pairs for which less than five dose-response measurements were provided. Furthermore, we discarded all curves with a root mean squared error (RMSE) between the actual dose-response measures and the corresponding points on the curve greater than 0.3.

Finally, to derive the CMax viability from the dose-response curve of cell line i and drug j with CMax concentration $CMax_j$, the function of the fitted curve is evaluated at $CMax_j$. Intuitively, the CMax viability is the viability at the intersection point between the dose-response curve and a line parallel to the viability (Y) axis passing through the concentration $CMax_j$ (cf. Figure 1).

3 Selection of drugs for drug-centric analyses

To achieve a fair performance comparison between the IC50 values and the CMax viabilities, we only considered drugs where both values were available, which resulted in 107 (60 from GDSC1, 47 from GDSC2) potentially analyzable drugs. Furthermore, we discarded drugs with only one available class or an insufficient number of samples per available class. In particular, we need at least 12 samples of each class to allow for a partition into training, calibration, and test set (also during CV) such that each set can contain at least one sample from each class. We then ensured by repeated rounds of

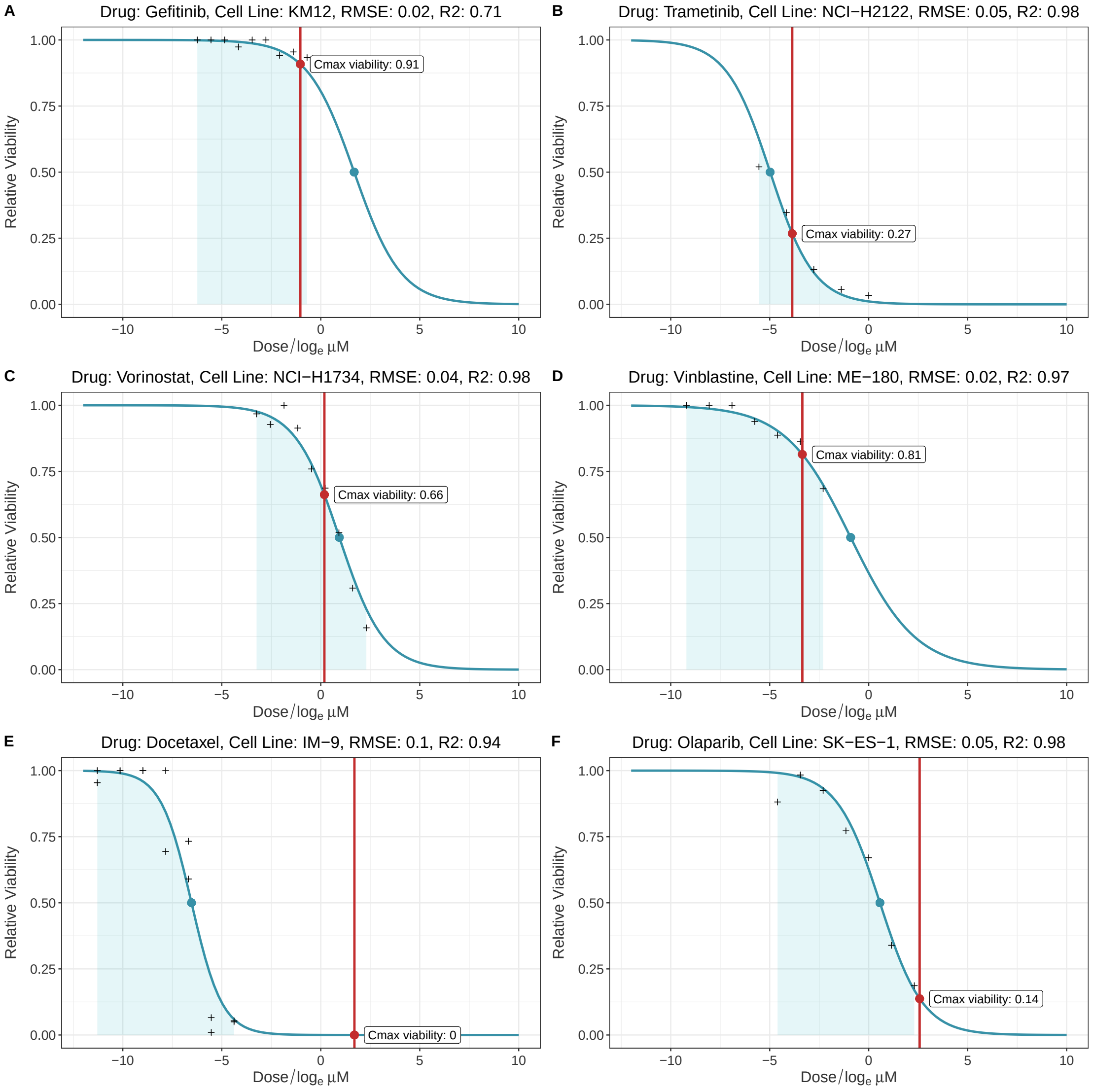

Figure 1: **Exemplary dose-response curves for six drug and cell line combinations.** The black crosses denote the dose-response measurements provided by the GDSC database. The dose-response curve that was fit to these points by applying the multilevel mixed effects model by [2] is shown in blue. The blue area under the curve highlights the tested concentration range and the blue circle, where the curve reaches a relative viability of 0.5 marks the IC50 concentration. The red vertical line marks the CMax concentration of the considered drug and the red circle, where the curves reaches the CMax concentration, denotes the CMax viability.

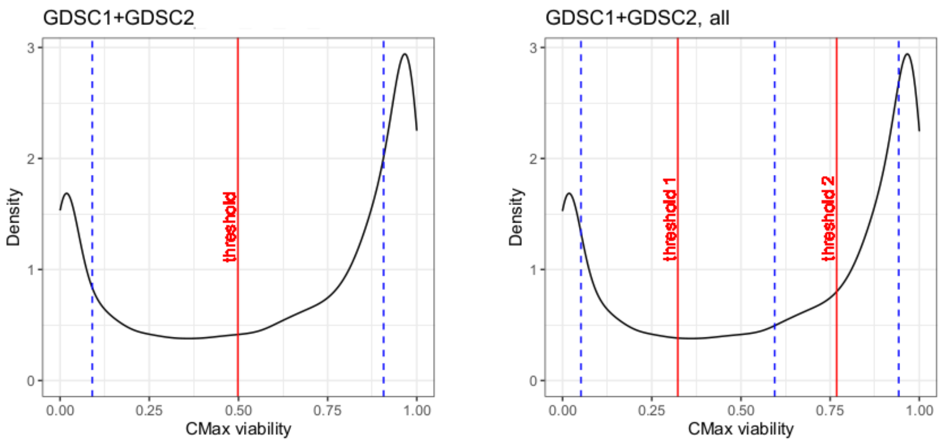

(a) One threshold

(b) Two thresholds

Figure 2: **Threshold determination.** This figure depicts the density of the CMax viability values and the derived thresholds (red lines) as well as the medoids (blue dashed lines) resulting from applying PAM. The threshold between sensitive and resistant samples in Figure (a) is located at 0.5. The thresholds in Figure (b) are located at 0.32 (sensitive vs ambiguous) and 0.77 (ambiguous vs resistant).

sampling if needed that the training sets (also during CV) contained at least one sample from each class such that the ML method can learn to distinguish between classes. For the calibration step, the True-class and the Summation score do not require each class to be present in the calibration set, while the Mondrian score does. We decided not to impose constraints to ensure this but instead discarded drugs where this condition was not fulfilled. In Figures 3 - 6, we visualize the selection process.

Table 2: Example drug prioritization using IC50 values. This table depicts the results of performing drug prioritization on IC50 values for an example cell line (COSMIC ID 905936). Shown are eight drugs for which the IC50 of cell line 905936 was below the drug-specific IC50 threshold obtained using a binarization procedure by [7]. Drugs are ordered from smallest to largest IC50. Additionally, the CMax concentrations according to [28] and IC50 thresholds are provided. The last two columns denote, whether the IC50 value for each drug is greater than the CMax concentration and IC50 threshold, respectively

	Drug	IC50 of CL 905936	CMax conc.	IC50 thr.	IC50 > CMax	IC50 > thr.
1	Trametinib	-3.56	-3.86	-1.40	TRUE	FALSE
2	Cytarabine	-1.85	4.00	-0.52	FALSE	FALSE
3	Crizotinib	0.85	-0.09	2.05	TRUE	FALSE
4	Oxaliplatin___1089	1.05	1.60	1.36	FALSE	FALSE
5	Nilotinib	2.15	-0.17	2.68	TRUE	FALSE
6	Dabrafenib	2.26	1.58	2.73	TRUE	FALSE
7	Oxaliplatin___1806	2.48	1.60	3.64	TRUE	FALSE
8	Carmustine	4.81	2.97	4.84	TRUE	FALSE

4 Selection of drugs for drug prioritization

Section 2.4 of the main manuscript presents the results of our drug-prioritization analysis: Given a particular cell line, the goal is to first identify the effective drugs and to then sort these drugs by their predicted efficiency. For this analysis, we decided to employ only drugs with at least 6% of samples in each class, since models for drugs with a

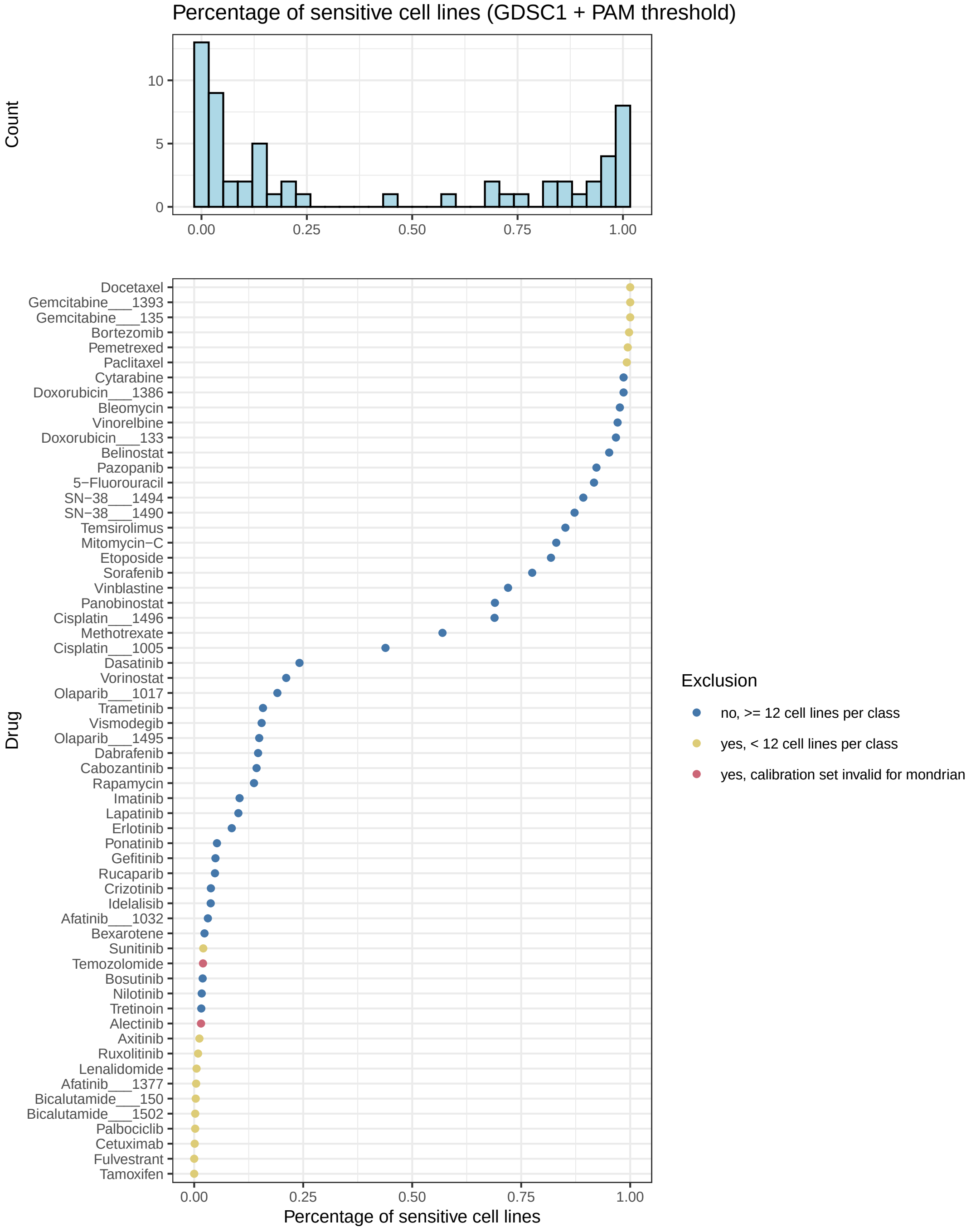

Figure 3: **Percentage of sensitive cell lines of binarized CMax viability in GDSC1.** The upper row of this figure shows a histogram for the percentage of sensitive cell lines across all available drugs. The lower row depicts the corresponding percentage for each drug. Moreover, the color of the points indicates which drugs had to be excluded for the analysis and why.

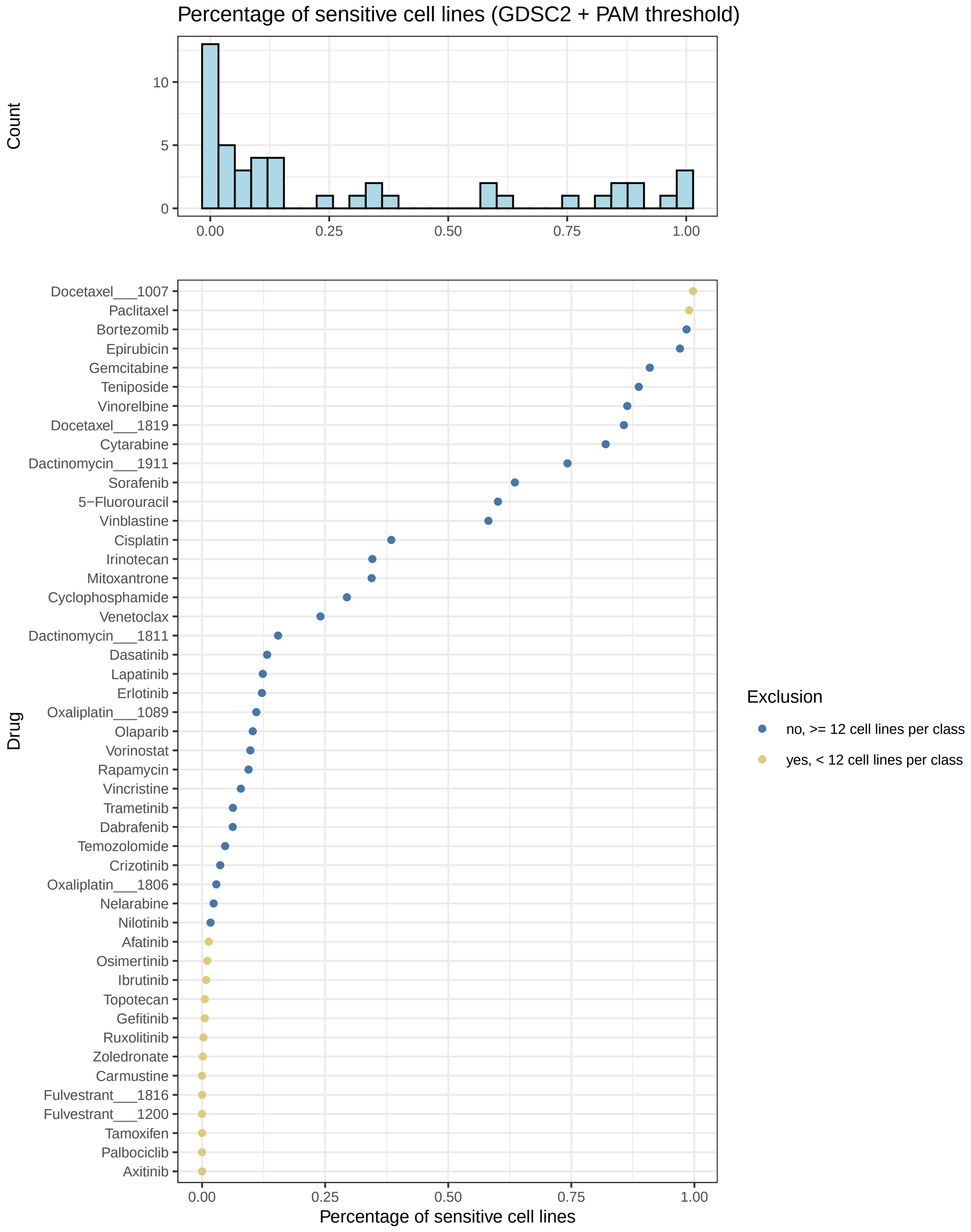

Figure 4: **Percentage of sensitive cell lines of binarized CMax viability in GDSC2.** The upper row of this figure shows a histogram for the percentage of sensitive cell lines across all available drugs. The lower row depicts the corresponding percentage for each drug. Moreover, the color of the points indicates which drugs had to be excluded for the analysis and why.

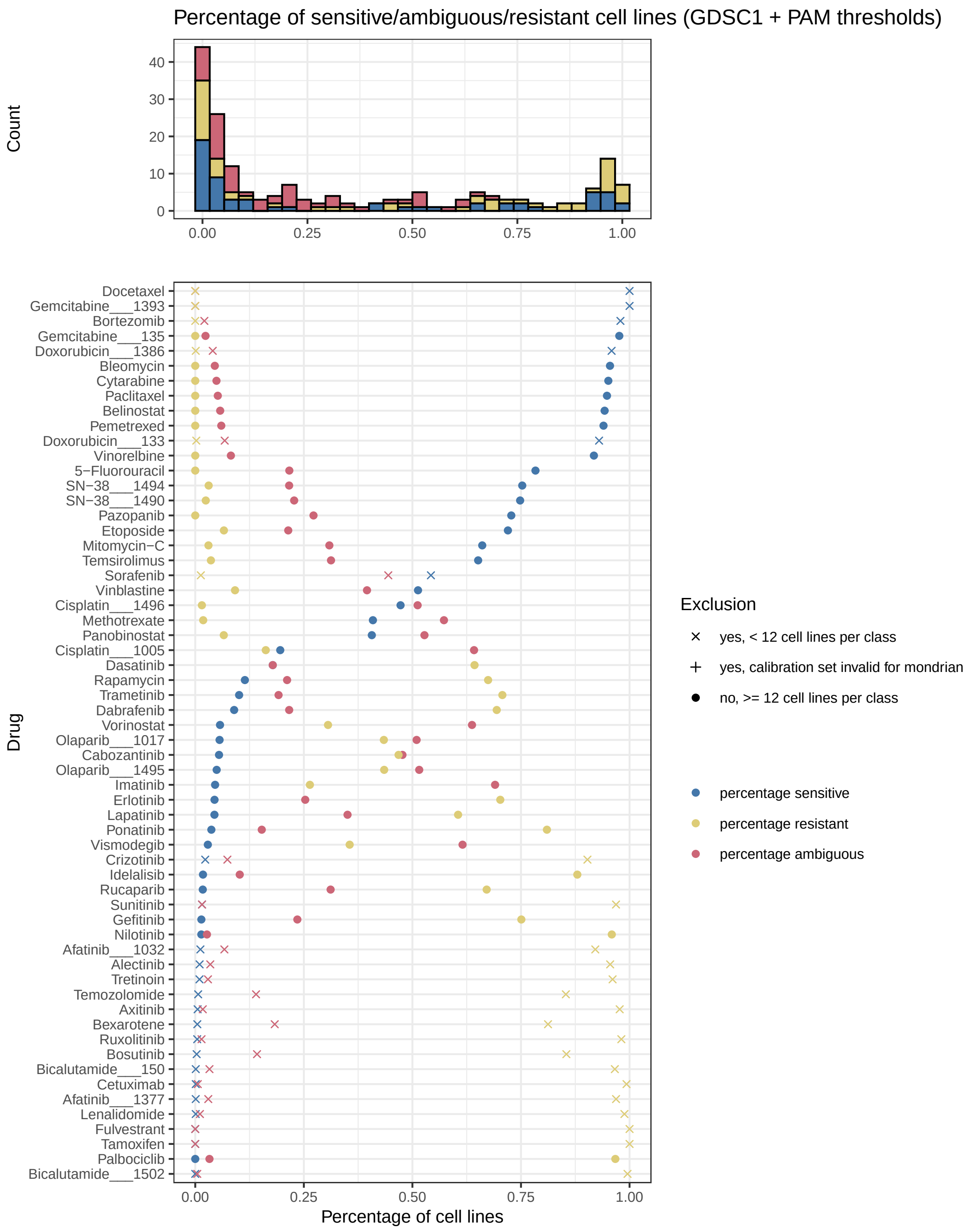

Figure 5: **Percentage of sensitive, ambiguous, and resistant cell lines of ternary CMax viability in GDSC1.** The upper row of this figure shows a histogram for the percentage of sensitive, ambiguous and resistant cell lines across all available drugs. The lower row depicts the corresponding percentages for each drug. Moreover, the shape of the points indicates which drugs had to be excluded for the analysis and why.

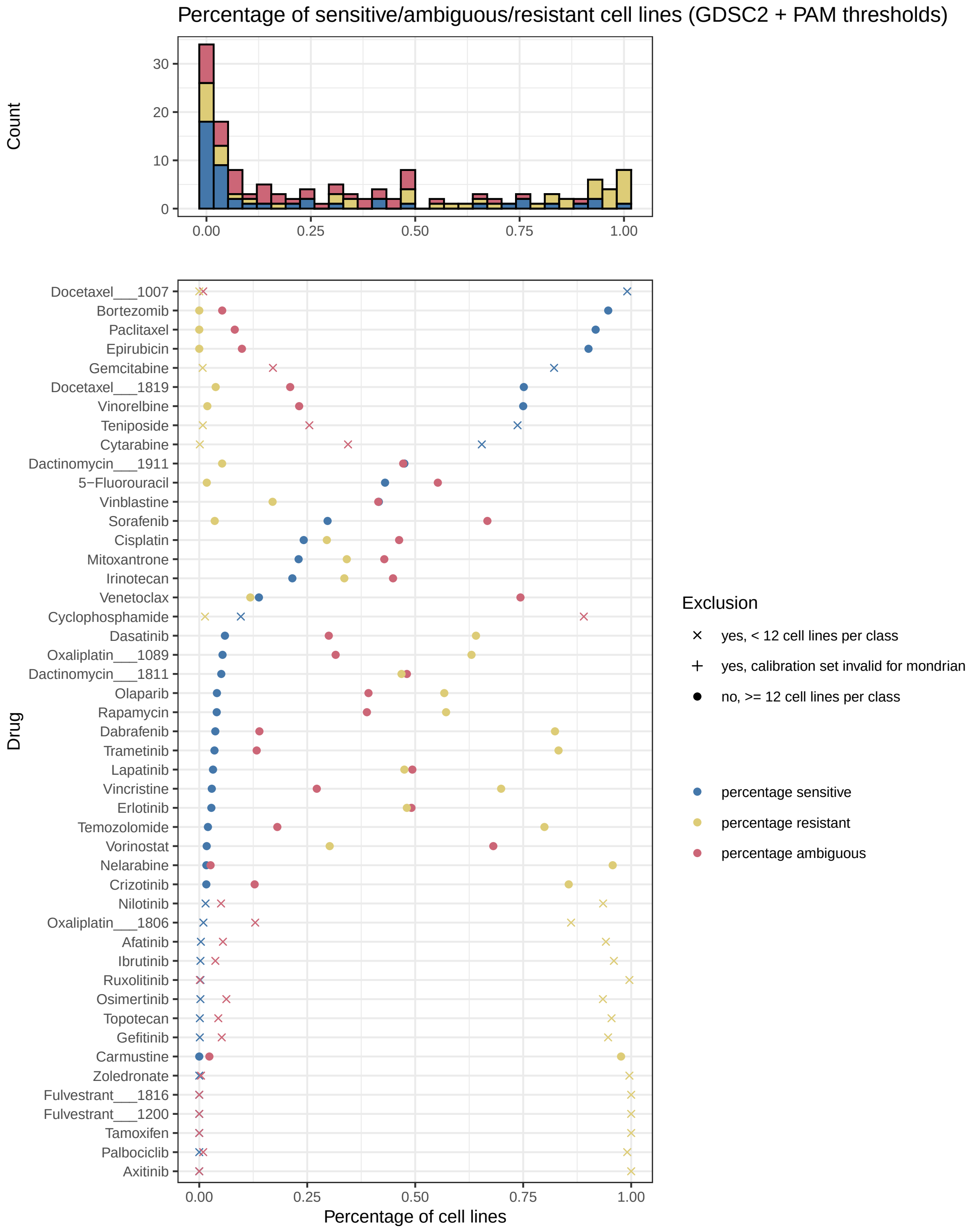

Figure 6: **Percentage of sensitive, ambiguous, and resistant cell lines of ternary CMax viability in GDSC2.** The upper row of this figure shows a histogram for the percentage of sensitive, ambiguous and resistant cell lines across all available drugs. The lower row depicts the corresponding percentages for each drug. Moreover, the shape of the points indicates which drugs had to be excluded for the analysis and why.

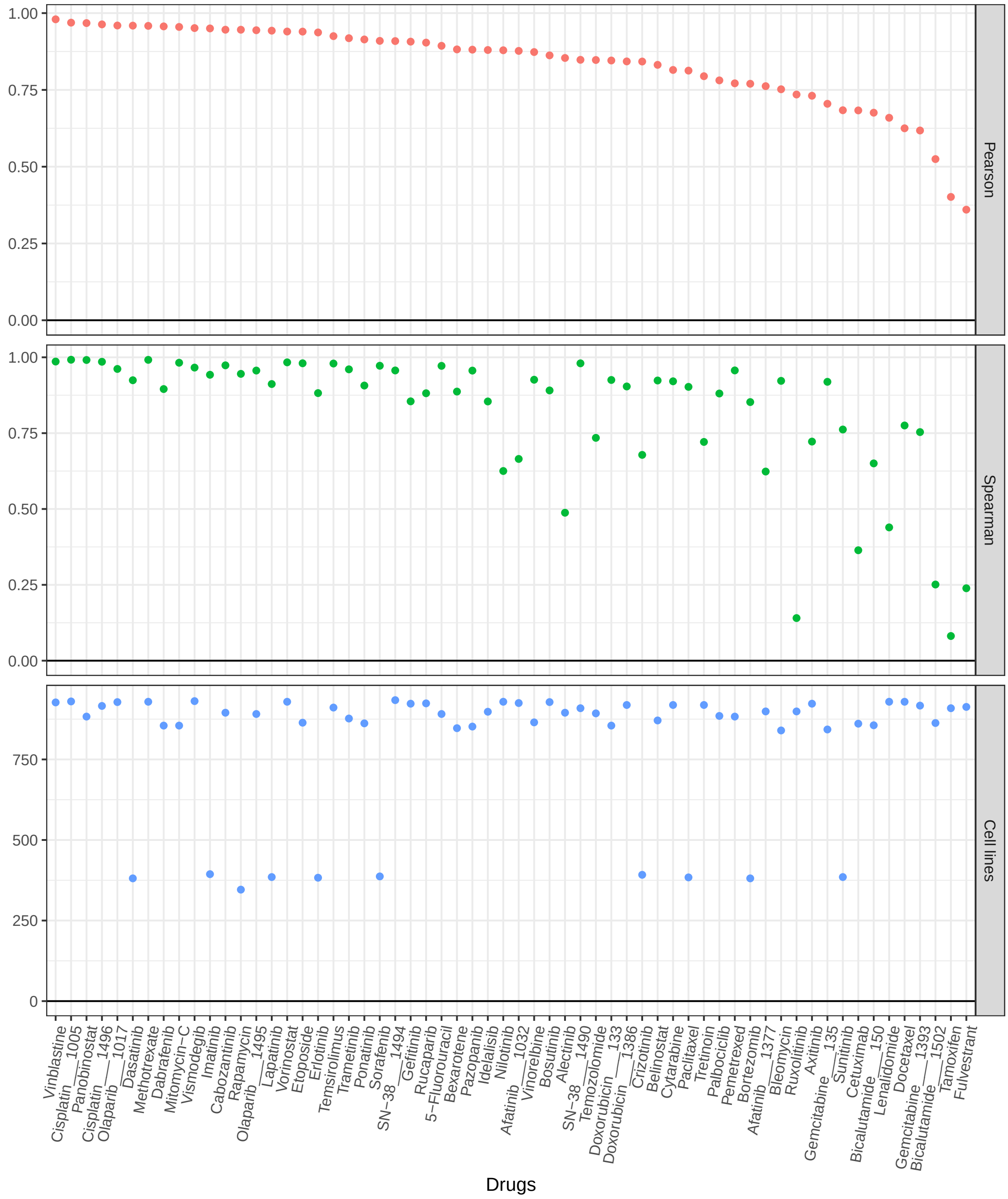

Figure 7: **Similarity between CMax viabilities and IC50 values for GDSC1.** This figure depicts the Pearson correlation, Spearman correlation, and the number of shared cell lines for all drugs with available CMax viabilities.

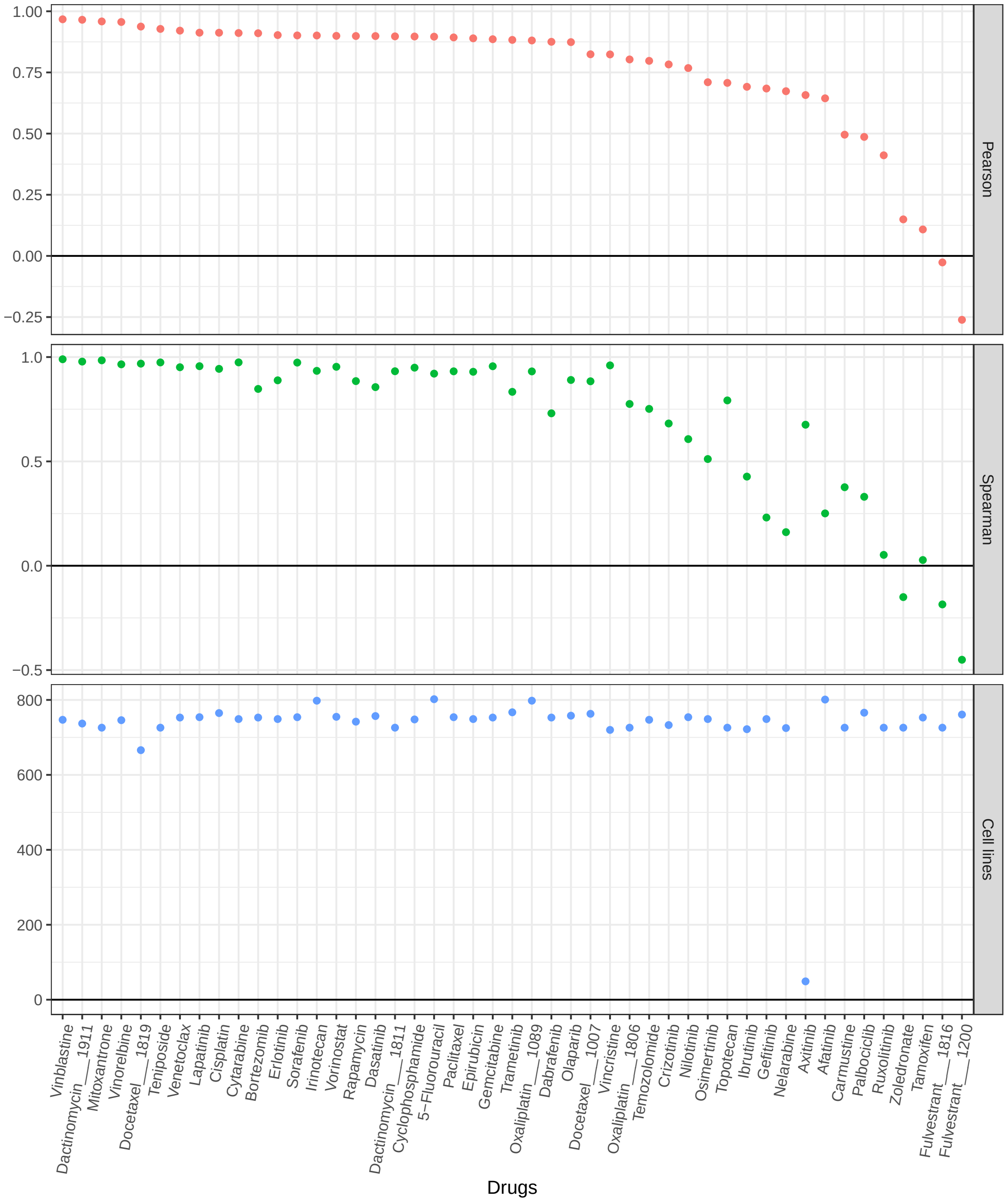

Figure 8: **Similarity between CMax viabilities and IC50 values for GDSC2.** This figure depicts the Pearson correlation, Spearman correlation, and the number of shared cell lines for all drugs with available CMax viabilities.

comparably low imbalance perform better than models trained on drugs with high class imbalance. Beyond that, the CP certainty guarantee also becomes more accurate, the more calibration samples are available, see [3, 4], which is particularly important for the minority class. In total, this constraint reduced the number of drugs to 25 for GDSC1 and 25 for GDSC2.

To prioritize drugs for one particular cell line in a realistic application case, it is necessary that this cell line is previously unseen by each drug-specific model, i.e., all drugs have to share the cell lines in the test set. To this end, we first determined the common cell lines between all investigated drugs for each sub-database, resulting in 243 common cell lines for the GDSC1 drugs and 609 common cell lines for the GDSC2 drugs. For each sub-database, we then randomly sampled a test and calibration set from the common cell lines such that their sizes are approximately equal to the average sizes from our drug-centric analyses (cf. Section 2.3 of main manuscript). The calibration- and test-sets each contained 121 cell lines for GDSC1 and 152 cell lines for GDSC2. The remaining cell lines are added to the training sets of the drugs.

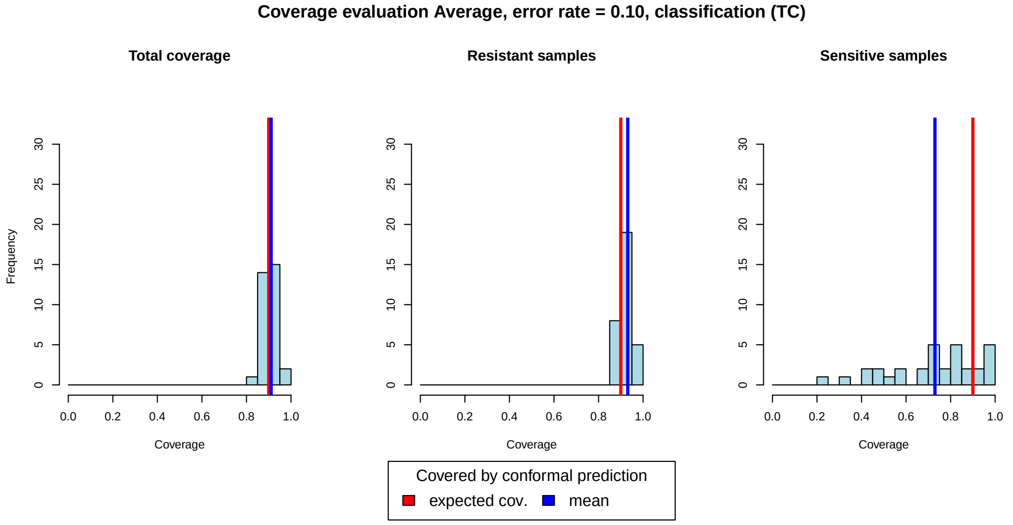

(a) True-class score

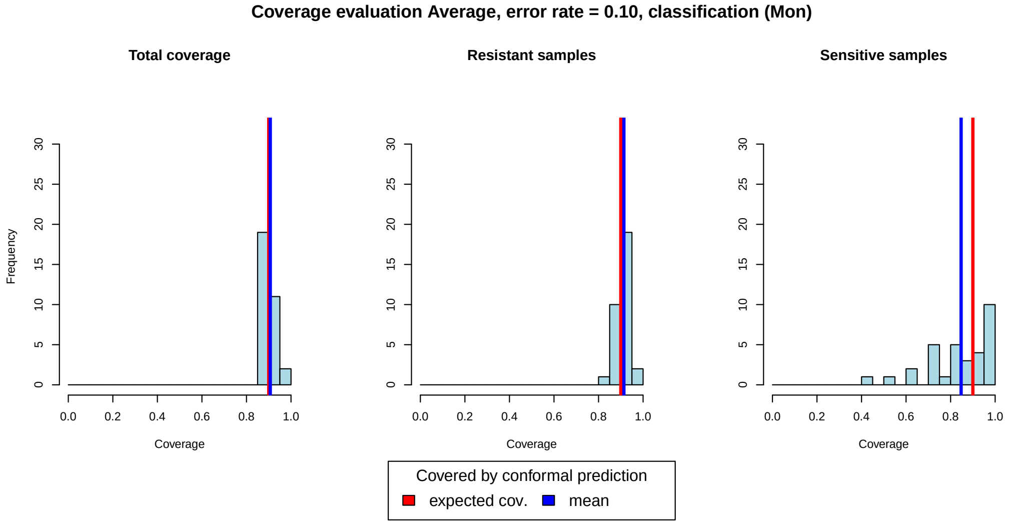

(b) Mondrian score

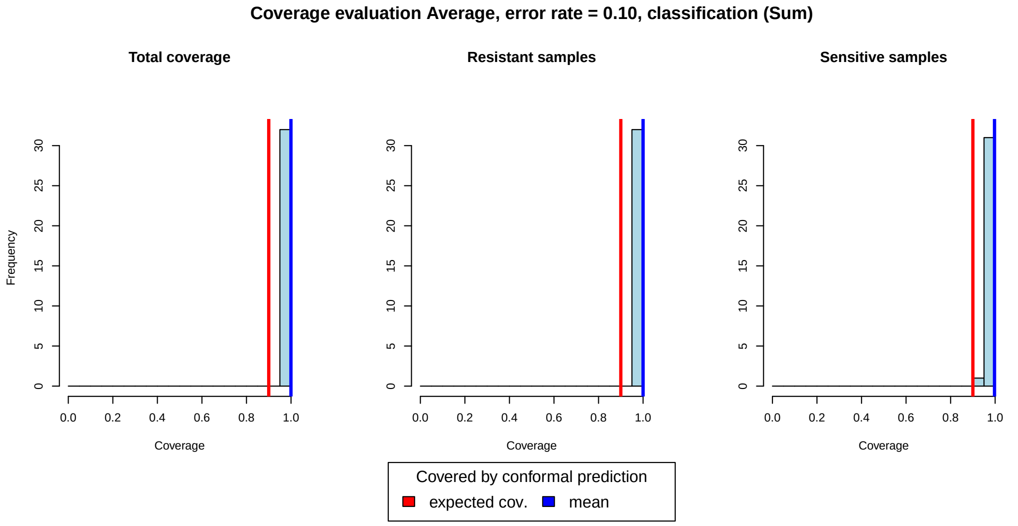

(c) Summation score

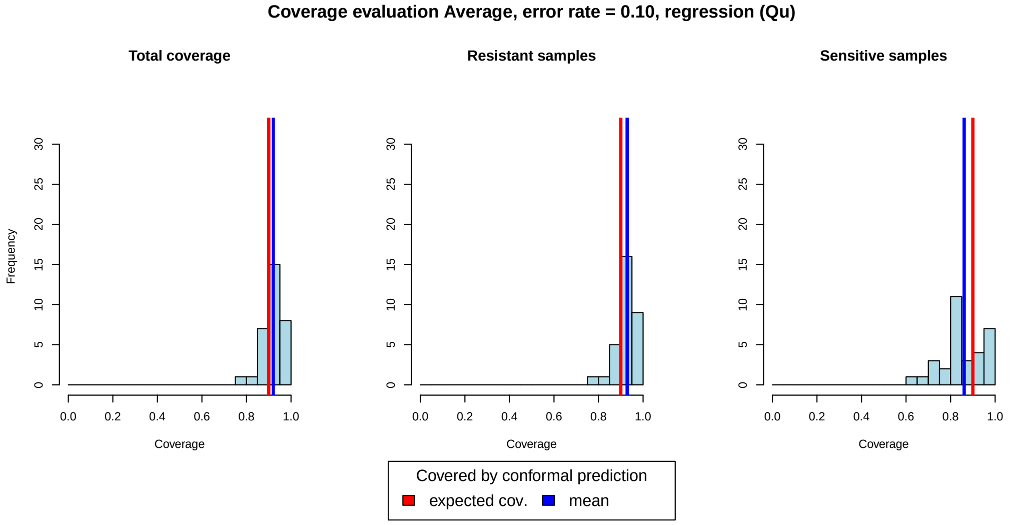

(d) Quantile score

Figure 9: **Coverage evaluation for CP models of 32 drugs from the GDSC2 database trained using IC50 values and a two-class classification setting.** This figure depicts histograms of the coverage property across CP models for 32 drugs obtained from the GDSC2 database. The left plot in each sub-figure depicts the total coverage, i.e. the fraction of cell lines from the test set of each drug, for which the true response was part of the predicted set / interval. The middle and right plots show the coverage for subsets of resistant and sensitive cell lines for each drug, respectively. The expected coverage of 0.9 for the employed error rate of $\alpha = 0.1$ is shown in red, the actual mean coverage over all investigated drugs is shown in blue. Sub-Figures (a), (b) and (c) depict histograms for the classification setting using the True-class (TC), Mondrian (Mon) and Summation (Sum) scoring functions, respectively. Sub-Figure (d) shows histograms for the regressions setting, where CP was performed using the Quantile (Qu) scoring function.

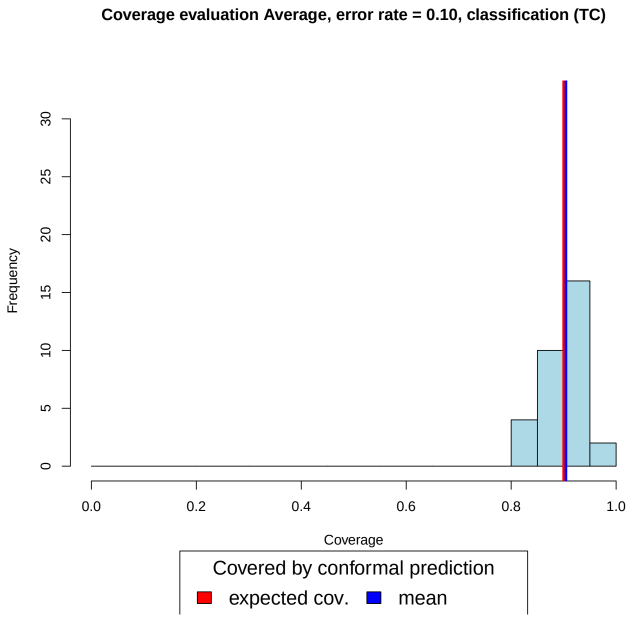

(a) True-class score

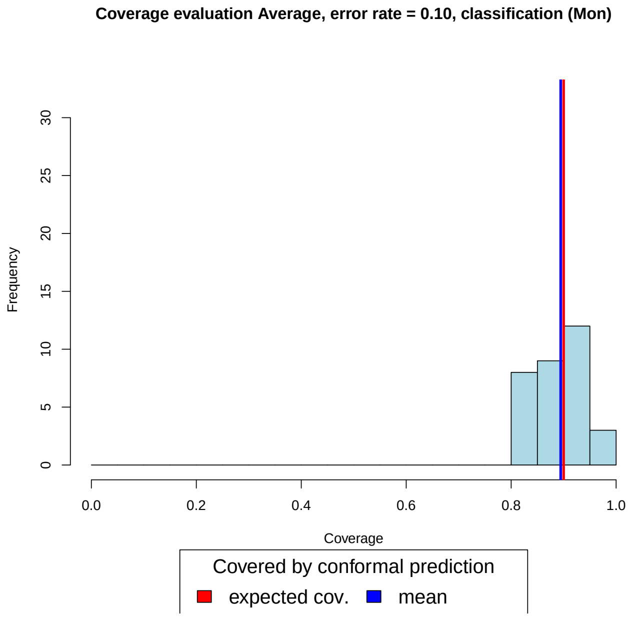

(b) Mondrian Score

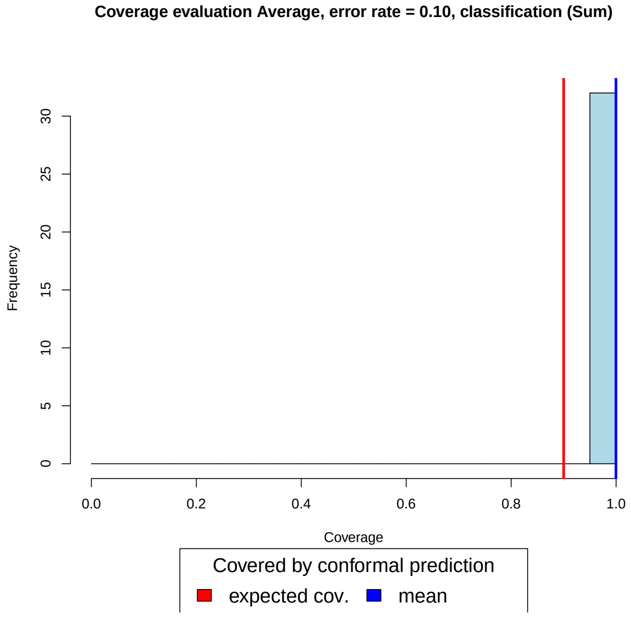

(c) Summation score

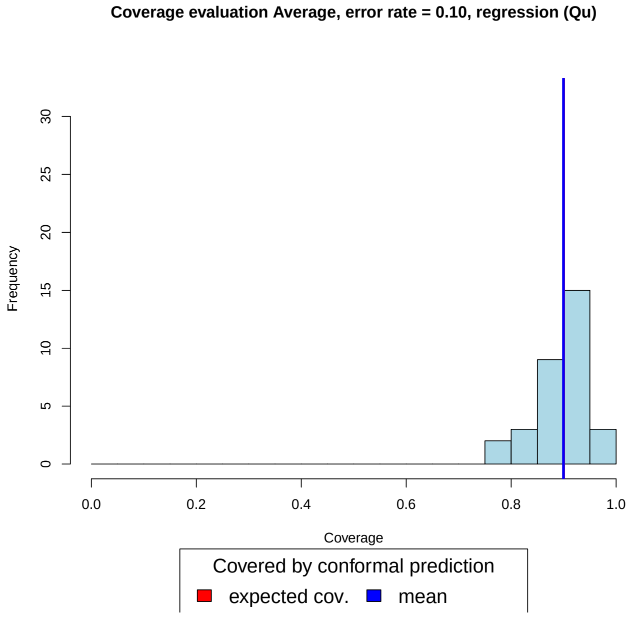

(d) Quantile score

Figure 10: **Coverage evaluation for CP models of 32 drugs from the GDSC2 database trained using CMax viability values and a two-class classification setting.** This figure depicts histograms of the coverage property across CP models for 32 drugs obtained from the GDSC2 database. The coverage is computed as the fraction of cell lines from the test set of each drug, for which the true response was part of the predicted set / interval. The expected coverage of 0.9 for the employed error rate of $\alpha = 0.1$ is shown in red, the actual mean coverage over all investigated drugs is shown in blue. Sub-Figures (a), (b) and (c) depict histograms for the classification setting using the True-class (TC), Mondrian (Mon) and Summation (Sum) scoring functions, respectively. Sub-Figure (d) shows histograms for the regressions setting, where CP was performed using the Quantile (Qu) scoring function.

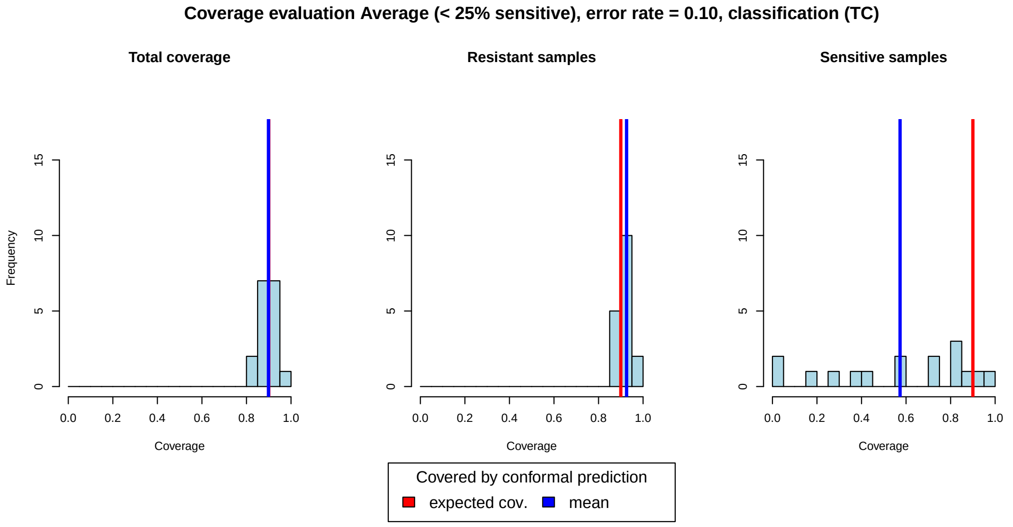

(a) True-class score

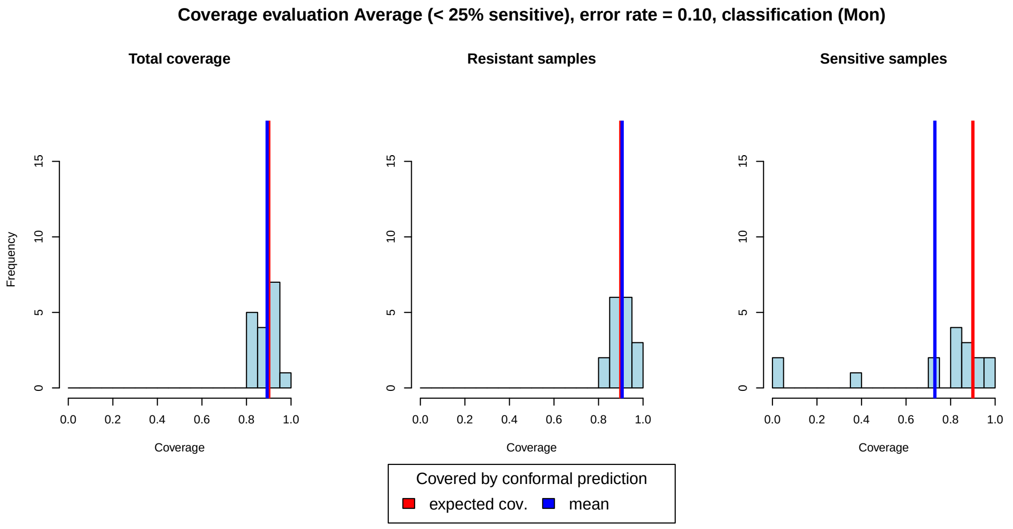

(b) Mondrian score

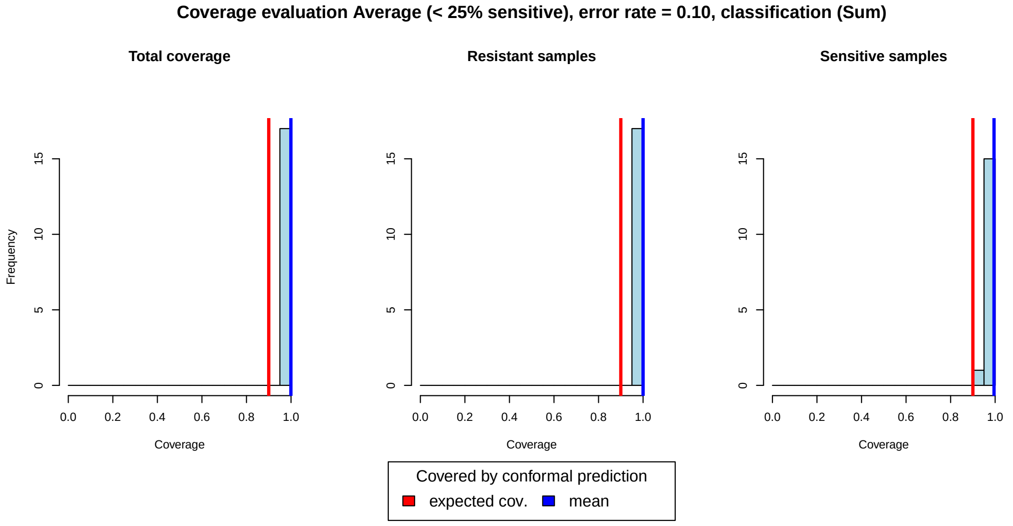

(c) Summation score

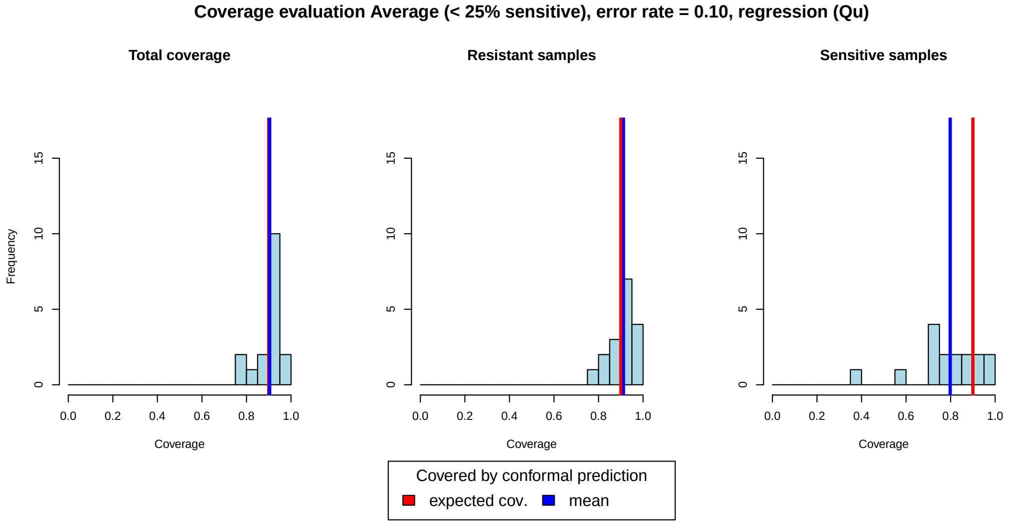

(d) Quantile score

Figure 11: **Coverage evaluation for CP models of 17 drugs from the GDSC2 database trained using CMax viability values and a two-class classification setting with underrepresentation of the sensitive class.** This figure depicts histograms of the coverage property across CP models for 17 drugs obtained from the GDSC2 database, for which the number of sensitive cell lines was less than 25%. The left plot in each sub-figure depicts the total coverage, i.e. the fraction of cell lines from the test set of each drug, for which the true response was part of the predicted set / interval. The middle and right plots show the coverage for subsets of resistant and sensitive cell lines for each drug, respectively. The expected coverage of 0.9 for the employed error rate of $\alpha = 0.1$ is shown in red, the actual mean coverage over all investigated drugs is shown in blue. Sub-Figures (a), (b) and (c) depict histograms for the classification setting using the True-class (TC), Mondrian (Mon) and Summation (Sum) scoring functions, respectively. Sub-Figure (d) shows histograms for the regressions setting, where CP was performed using the Quantile (Qu) scoring function.

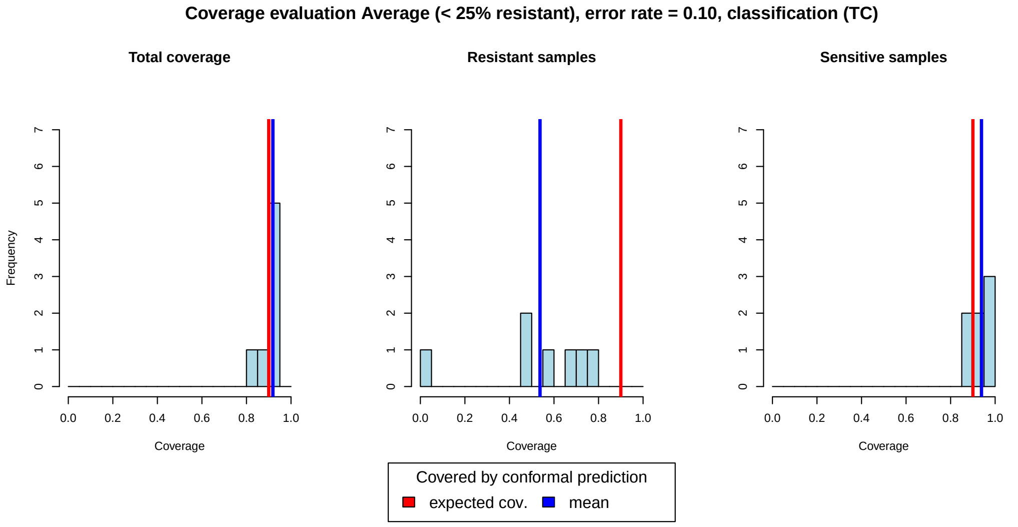

(a) True-class score

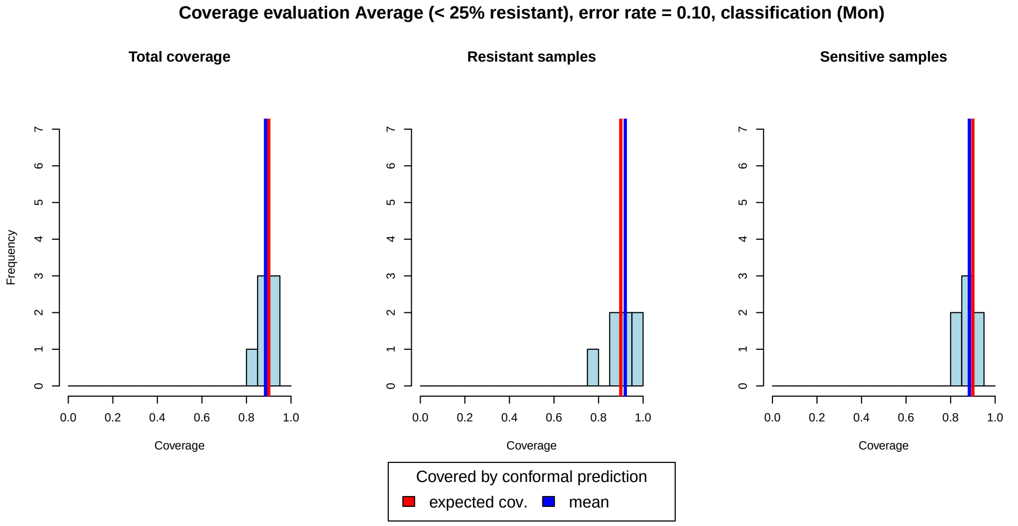

(b) Mondrian score

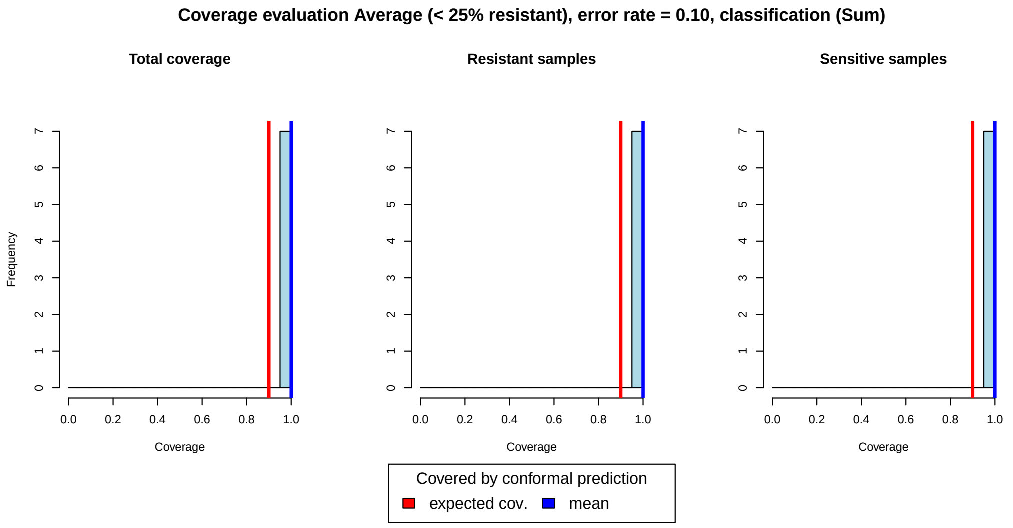

(c) Summation score

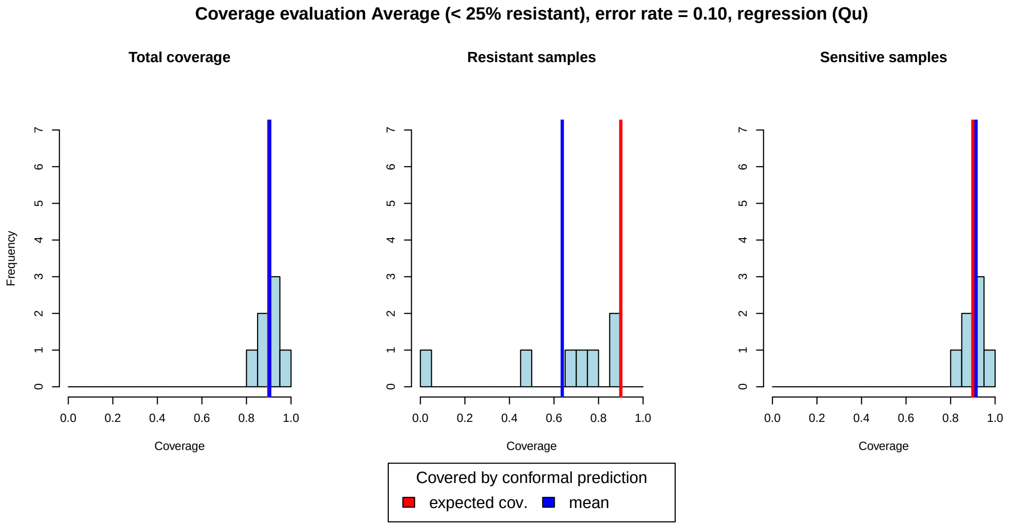

(d) Quantile score

Figure 12: **Coverage evaluation for CP models of seven drugs from the GDSC2 database trained using CMax viability values and a two-class classification setting with underrepresentation of the resistant class.** This figure depicts histograms of the coverage property across CP models for seven drugs obtained from the GDSC2 database, for which the number of resistant cell lines was less than 25%. The left plot in each sub-figure depicts the total coverage, i.e. the fraction of cell lines from the test set of each drug, for which the true response was part of the predicted set / interval. The middle and right plots show the coverage for subsets of resistant and sensitive cell lines for each drug, respectively. The expected coverage of 0.9 for the employed error rate of $\alpha = 0.1$ is shown in red, the actual mean coverage over all investigated drugs is shown in blue. Sub-Figures (a), (b) and (c) depict histograms for the classification setting using the True-class (TC), Mondrian (Mon) and Summation (Sum) scoring functions, respectively. Sub-Figure (d) shows histograms for the regressions setting, where CP was performed using the Quantile (Qu) scoring function.

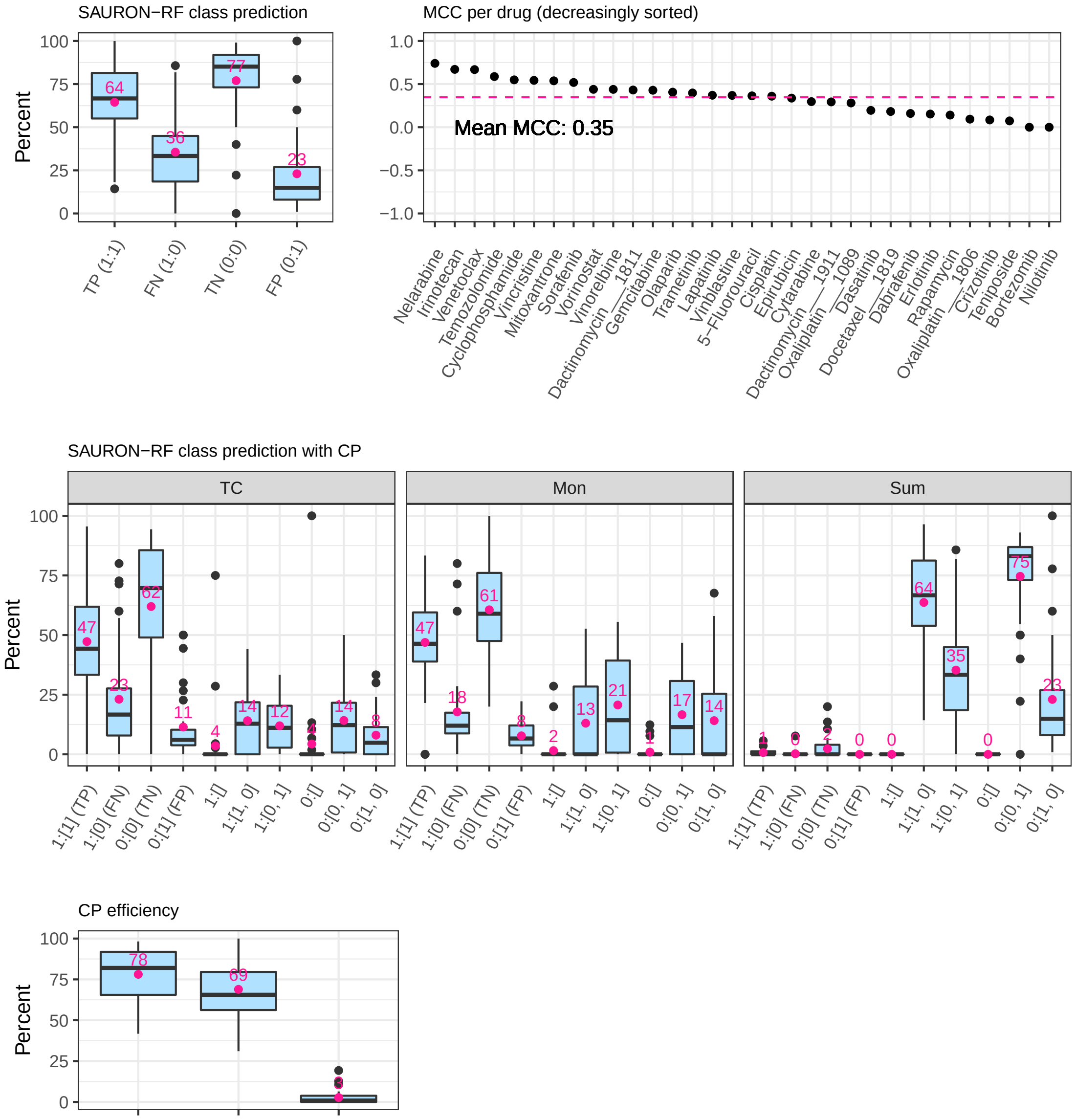

Figure 13: **Classification test set performance for 32 drugs from the GDSC2 database trained using CMax viability values and a two-class classification setting.** The upper row of this figure depicts the classification performance of SAURON-RF across the different drugs from GDSC2. The middle row shows the effects of CP on the performance in terms of true positive/negative predictions. In the lower row, the CP efficiency is presented.

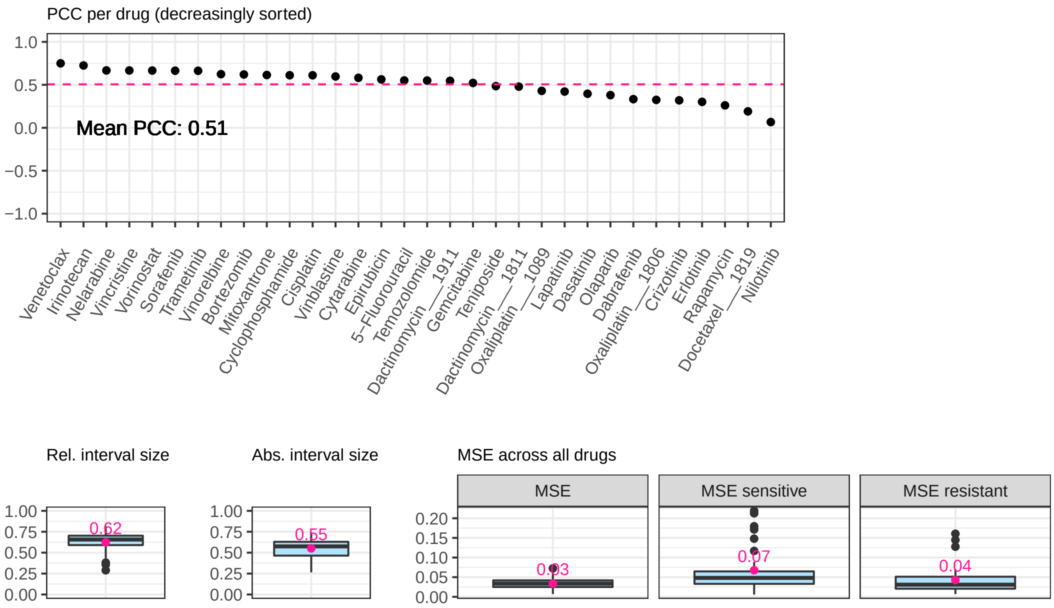

Figure 14: **Regression test set performance for 32 drugs from the GDSC2 database trained using CMax viability values and a two-class classification setting.** The upper row of this figure depicts the Pearson correlation coefficient between the actual continuous response values and the predicted continuous response values for all drugs. The lower row shows the mean-squared error (MSE) and the interval width of the CP Quantile regression score relative to the spanned training ranges of the drugs.

Table 3: Investigated drugs from GDSC1. This table lists all investigated drugs from the GDSC1 dataset, including the number of available cell lines and whether each drug was used for the two-class, three-class, and prioritization analyses, respectively.

	Drug	Cell lines	Two classes	Three classes	Prioritization
1	SN-38___1494	952	yes	yes	yes
2	Vismodegib	949	yes	yes	yes
3	Cisplatin___1005	948	yes	yes	yes
4	Vorinostat	948	yes	yes	yes
5	Methotrexate	947	yes	yes	yes
6	Nilotinib	947	yes	yes	no
7	Olaparib___1017	946	yes	yes	yes
8	Bosutinib	946	yes	no	no
9	Vinblastine	945	yes	yes	yes
10	Afatinib___1032	943	yes	no	no
11	Gefitinib	941	yes	yes	no
12	Rucaparib	940	yes	yes	no
13	Cytarabine	937	yes	yes	no
14	Tretinoin	937	yes	no	no
15	Doxorubicin___1386	934	yes	no	no
16	Cisplatin___1496	931	yes	yes	yes
17	Temsirolimus	928	yes	yes	yes
18	SN-38___1490	924	yes	yes	yes
19	Idelalisib	920	yes	yes	no
20	Cabozantinib	917	yes	yes	yes
21	5-Fluorouracil	913	yes	yes	yes
22	Olaparib___1495	906	yes	yes	yes
23	Pemetrexed	905	no	yes	no
24	Panobinostat	904	yes	yes	yes
25	Palbociclib	901	no	yes	no
26	Belinostat	892	yes	yes	no
27	Trametinib	892	yes	yes	yes
28	Vinorelbine	887	yes	yes	no
29	Etoposide	886	yes	yes	yes
30	Ponatinib	884	yes	yes	no
31	Doxorubicin___133	877	yes	no	no
32	Mitomycin-C	877	yes	yes	yes
33	Pazopanib	873	yes	yes	yes
34	Dabrafenib	870	yes	yes	yes
35	Bexarotene	869	yes	no	no
36	Gemcitabine___135	865	no	yes	no
37	Bleomycin	862	yes	yes	no
38	Imatinib	407	yes	yes	yes
39	Crizotinib	406	yes	no	no
40	Sorafenib	398	yes	no	yes
41	Paclitaxel	397	no	yes	no
42	Lapatinib	396	yes	yes	yes
43	Erlotinib	394	yes	yes	yes
44	Dasatinib	394	yes	yes	yes
45	Rapamycin	358	yes	yes	yes

Table 4: Investigated drugs from GDSC2. This table lists all investigated drugs from the GDSC2 dataset, including the number of available cell lines and whether each drug was used for the two-class, three-class, and prioritization analyses, respectively.

	Drug	Cell lines	Two classes	Three classes	Prioritization
1	5-Fluorouracil	806	yes	yes	yes
2	Oxaliplatin___1089	802	yes	yes	yes
3	Irinotecan	801	yes	yes	yes
4	Trametinib	771	yes	yes	yes
5	Cisplatin	768	yes	yes	yes
6	Olaparib	762	yes	yes	yes
7	Dasatinib	760	yes	yes	yes
8	Vorinostat	758	yes	yes	yes
9	Nilotinib	757	yes	no	no
10	Paclitaxel	757	no	yes	no
11	Sorafenib	757	yes	yes	yes
12	Dabrafenib	757	yes	yes	yes
13	Lapatinib	757	yes	yes	yes
14	Gemcitabine	756	yes	no	yes
15	Bortezomib	756	yes	yes	no
16	Venetoclax	756	yes	yes	yes
17	Cytarabine	752	yes	no	yes
18	Erlotinib	752	yes	yes	yes
19	Epirubicin	752	yes	yes	no
20	Cyclophosphamide	751	yes	no	yes
21	Vinblastine	750	yes	yes	yes
22	Temozolomide	750	yes	yes	no
23	Vinorelbine	750	yes	yes	yes
24	Rapamycin	745	yes	yes	yes
25	Dactinomycin___1911	740	yes	yes	yes
26	Crizotinib	737	yes	yes	no
27	Oxaliplatin___1806	728	yes	no	no
28	Carmustine	728	no	yes	no
29	Teniposide	728	yes	no	yes
30	Mitoxantrone	728	yes	yes	yes
31	Dactinomycin___1811	728	yes	yes	yes
32	Nelarabine	727	yes	yes	no
33	Vincristine	722	yes	yes	yes
34	Docetaxel___1819	669	yes	yes	yes

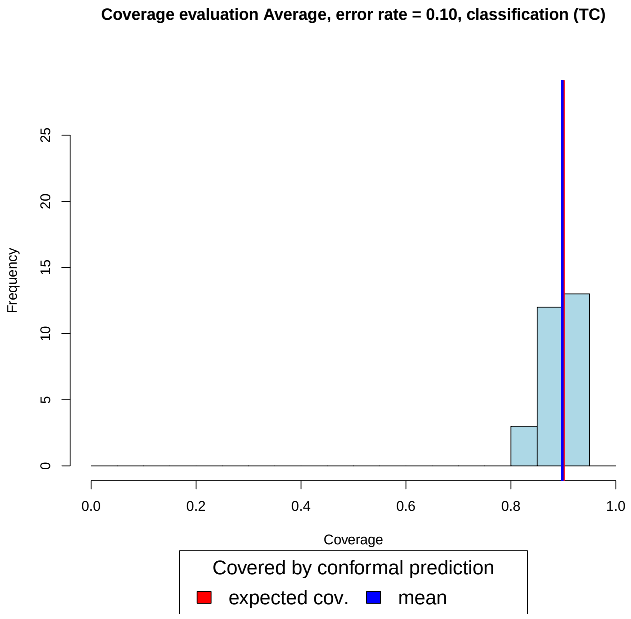

(a) True-class score

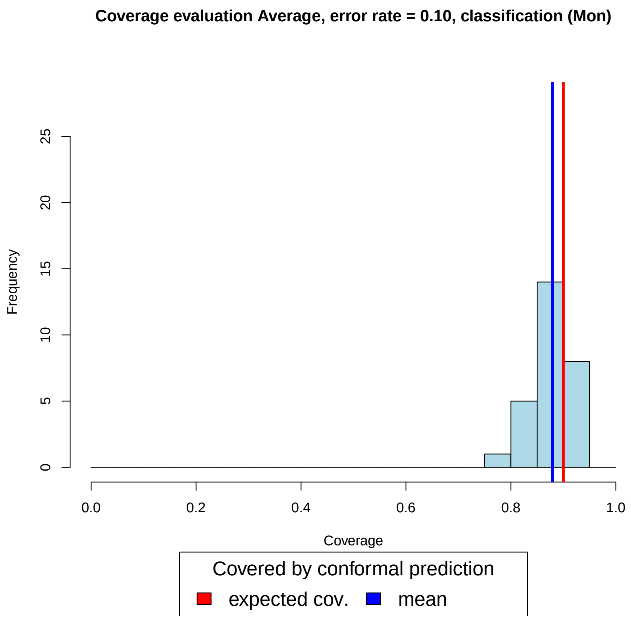

(b) Mondrian score

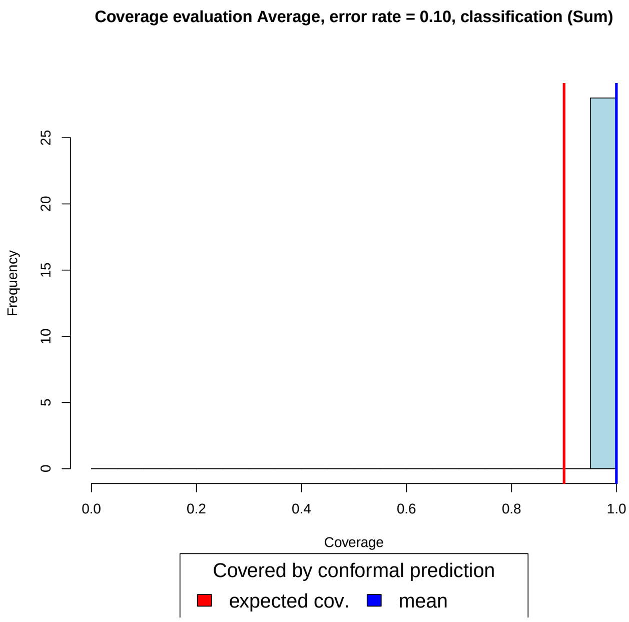

(c) Summation score

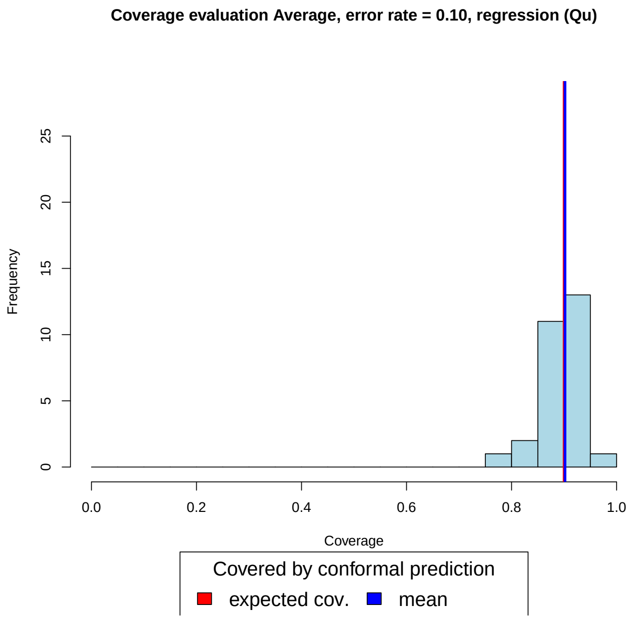

(d) Quantile score

Figure 15: **Coverage evaluation for CP models of 28 drugs from the GDSC2 database trained using CMax viability values and a three-class classification setting.** This figure depicts histograms of the coverage property across CP models for 28 drugs obtained from the GDSC2 database. The coverage is computed as the fraction of cell lines from the test set of each drug, for which the true response was part of the predicted set / interval. The expected coverage of 0.9 for the employed error rate of $\alpha = 0.1$ is shown in red, the actual mean coverage over all investigated drugs is shown in blue. Sub-Figures (a), (b) and (c) depict histograms for the classification setting using the True-class (TC), Mondrian (Mon) and Summation (Sum) scoring functions, respectively. Sub-Figure (d) shows histograms for the regressions setting, where CP was performed using the Quantile (Qu) scoring function.

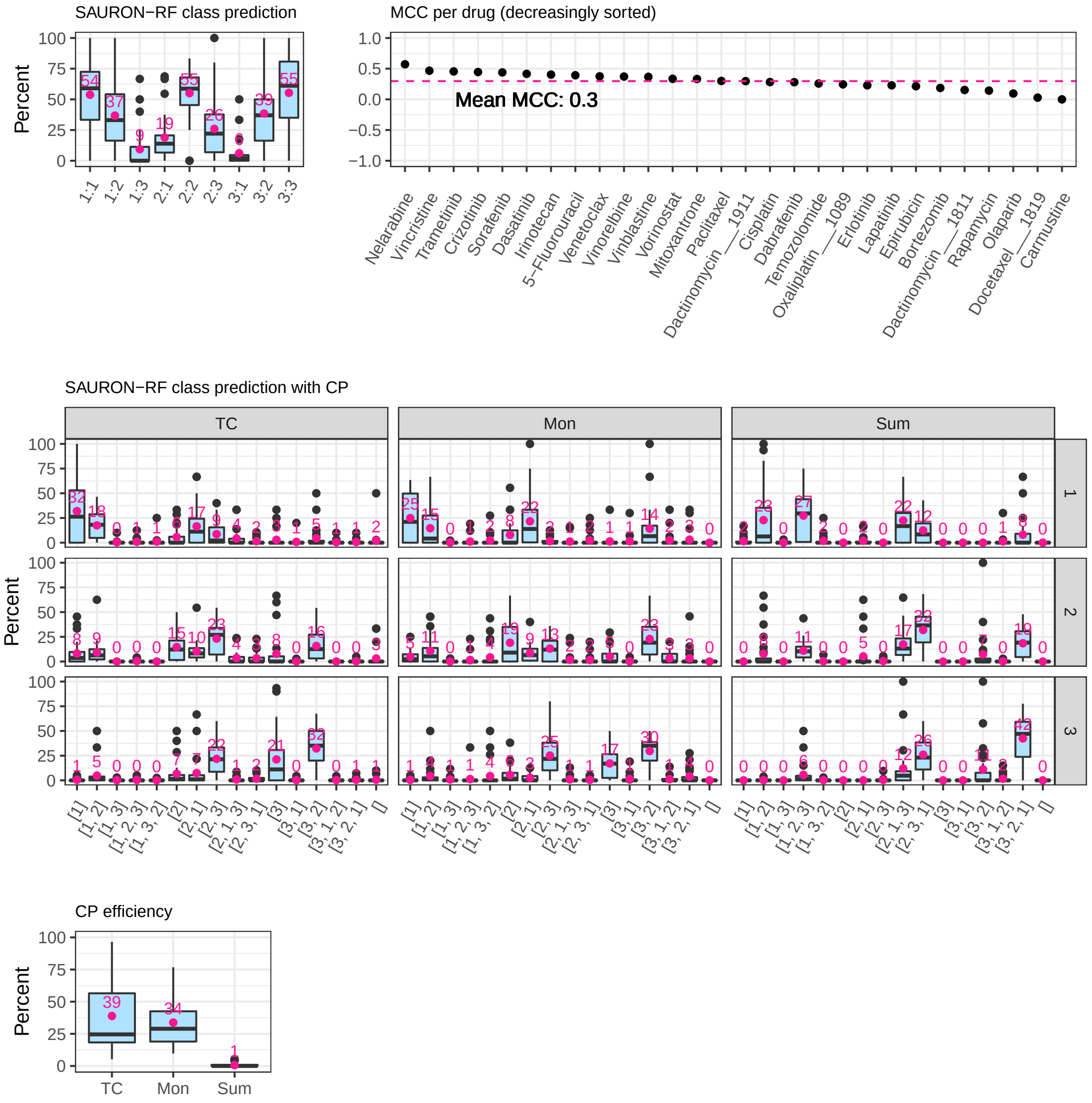

Figure 16: **Classification test set performance for 28 drugs from the GDSC2 database trained using CMax viability values and a three-class classification setting.** The upper row of this figure depicts the classification performance of SAURON-RF across the different drugs from GDSC2. The middle row shows the effects of CP on the performance. In the lower row, the CP efficiency is presented.

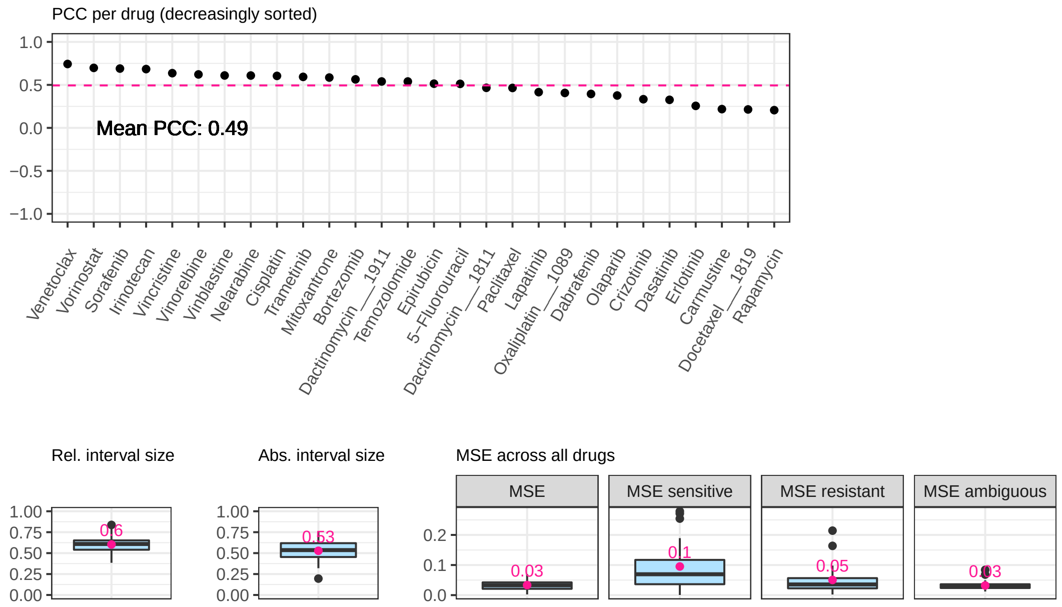

Figure 17: **Regression test set performance for 28 drugs from the GDSC2 database trained using CMax viability values and a three-class classification setting.** The upper row of this figure depicts the Pearson correlation coefficient between the actual continuous response values and the predicted continuous response values for all drugs. The lower row shows the mean-squared error (MSE) and the interval width of the CP Quantile regression score relative to the spanned training ranges of the drugs.

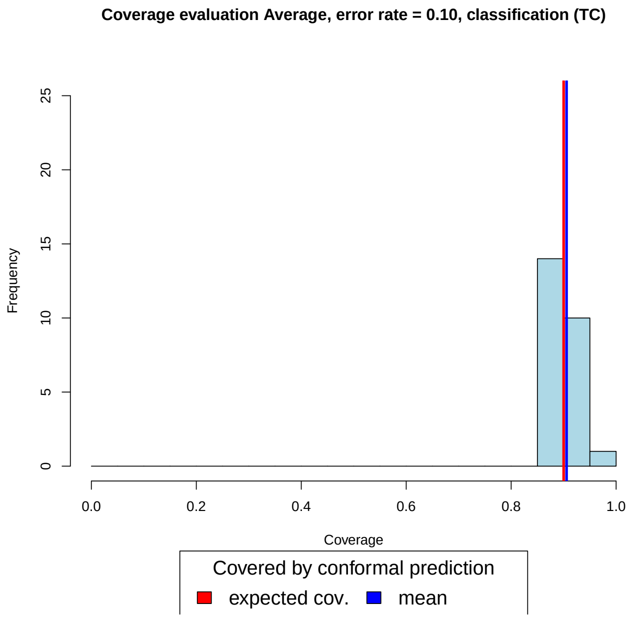

(a) True-class score

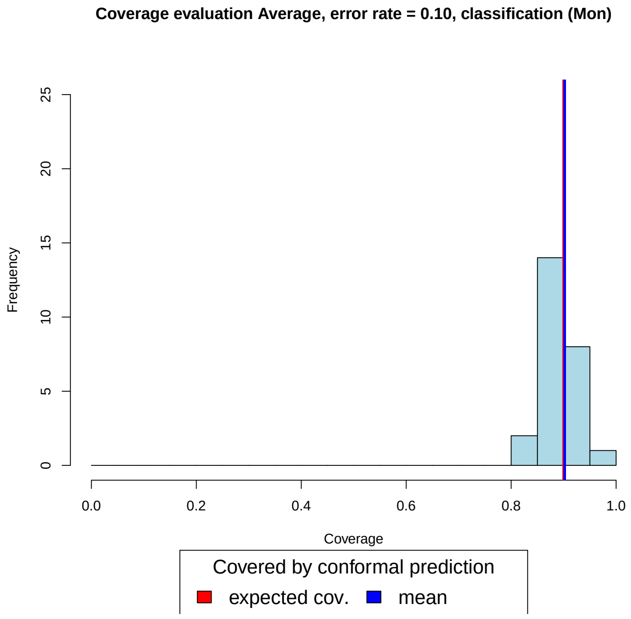

(b) Mondrian score

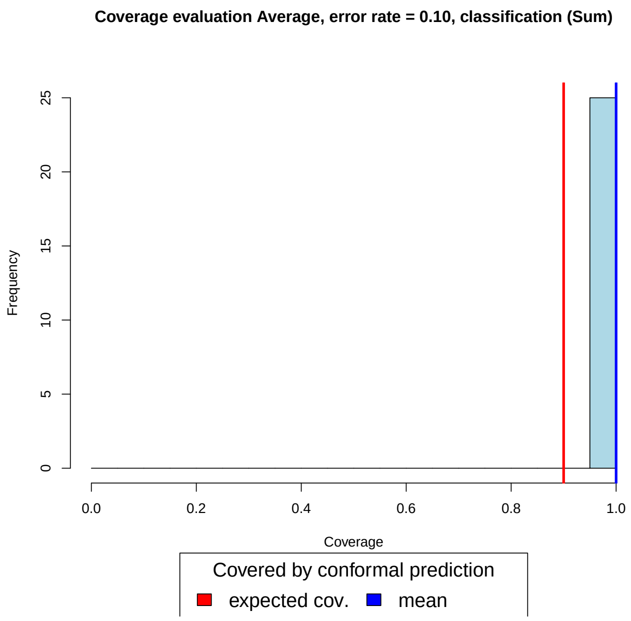

(c) Summation score

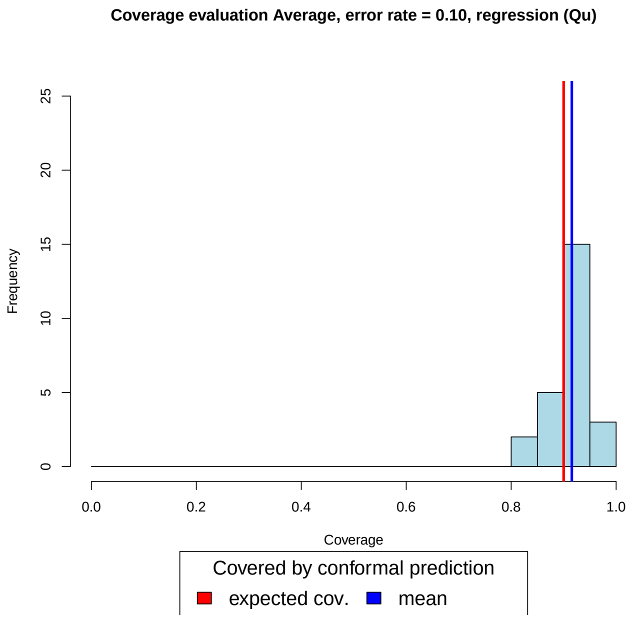

(d) Quantile score

Figure 18: **Coverage evaluation for CP models of 25 drugs from the GDSC2 database trained using CMax viability values and a two-class prioritization setting.** This figure depicts histograms of the coverage property across CP models for 25 drugs obtained from the GDSC2 database, which were used to conduct the drug prioritization analyses in Section 2.4 of the main manuscript. The coverage is computed as the fraction of cell lines from the test set of each drug, for which the true response was part of the predicted set / interval. The expected coverage of 0.9 for the employed error rate of $\alpha = 0.1$ is shown in red, the actual mean coverage over all investigated drugs is shown in blue. Sub-Figures (a), (b) and (c) depict histograms for the classification setting using the True-class (TC), Mondrian (Mon) and Summation (Sum) scoring functions, respectively. Sub-Figure (d) shows histograms for the regressions setting, where CP was performed using the Quantile (Qu) scoring function.

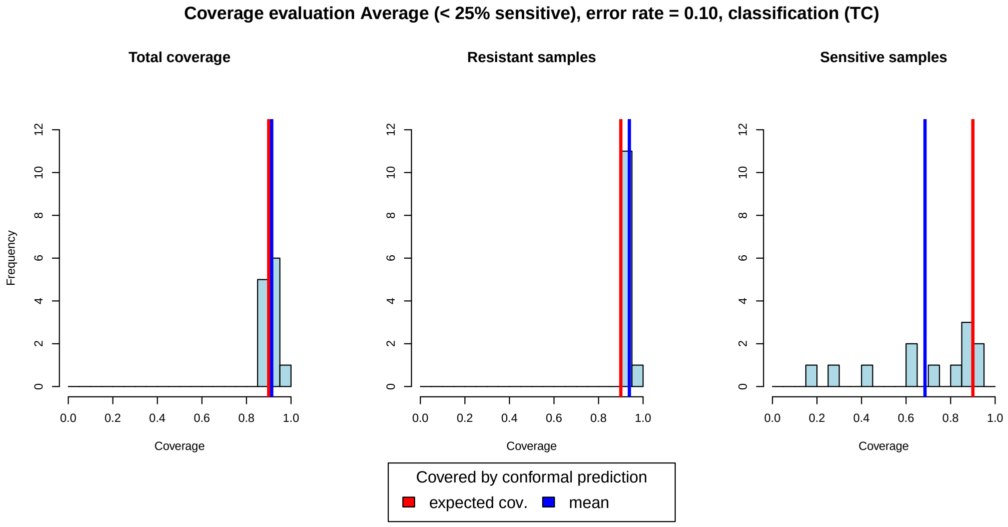

(a) True-class score

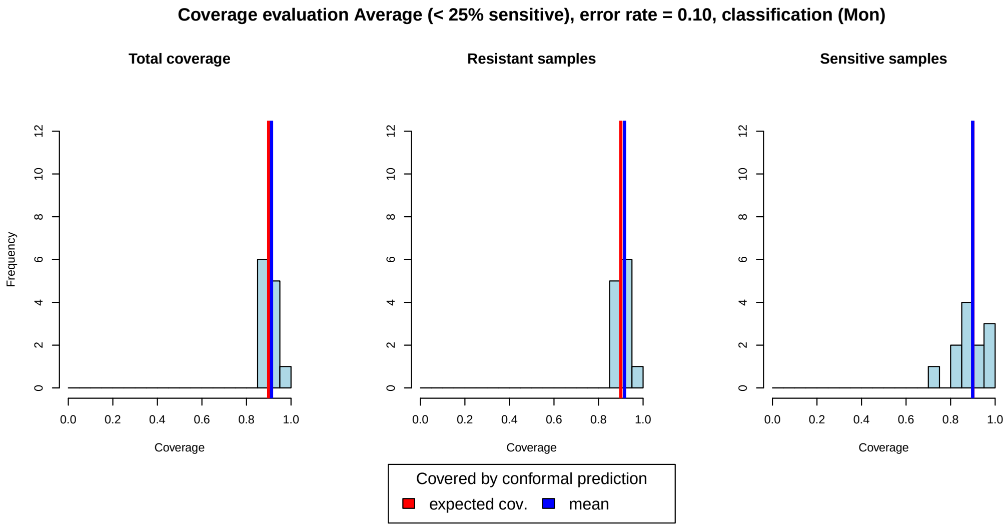

(b) Mondrian score

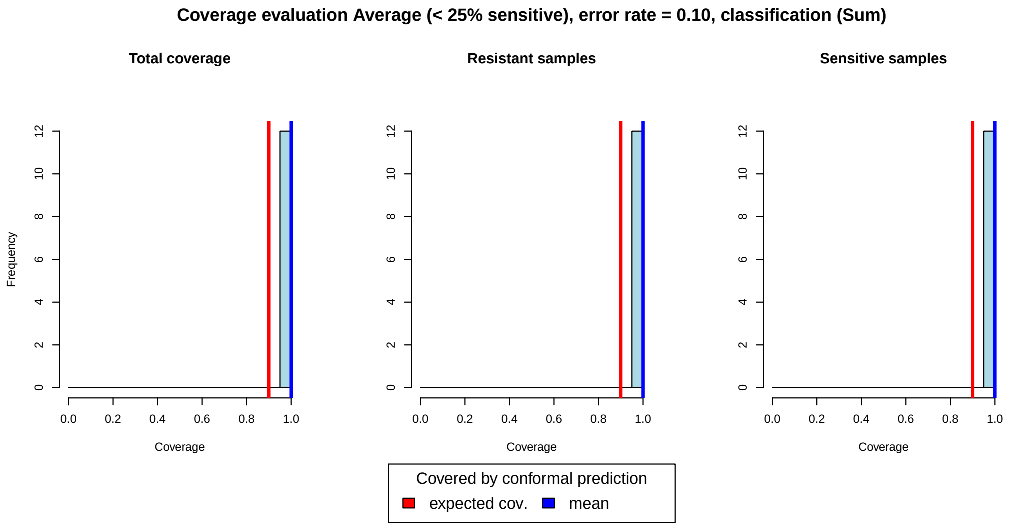

(c) Summation score

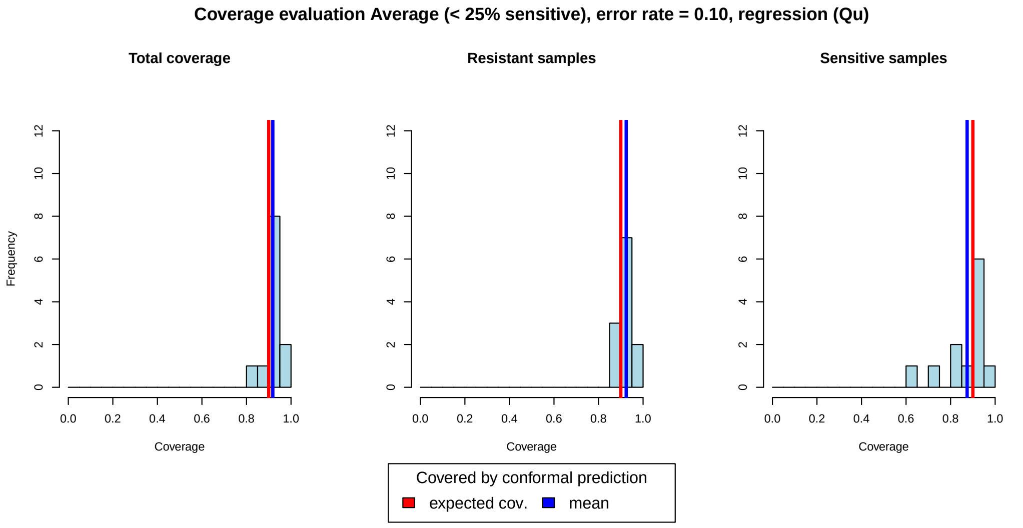

(d) Quantile score

Figure 19: **Coverage evaluation for CP models of twelve drugs from the GDSC2 database trained using CMax viability values and a two-class prioritization setting with underrepresentation of the sensitive class.** This figure depicts histograms of the coverage property across CP models for twelve drugs obtained from the GDSC2 database, for which the number of sensitive cell lines was less than 25%. These drugs were used to conduct the drug prioritization analyses in Section 2.4 of the main manuscript. The left plot in each sub-figure depicts the total coverage, i.e. the fraction of cell lines from the test set of each drug, for which the true response was part of the predicted set / interval. The middle and right plots show the coverage for subsets of resistant and sensitive cell lines for each drug, respectively. The expected coverage of 0.9 for the employed error rate of $\alpha = 0.1$ is shown in red, the actual mean coverage over all investigated drugs is shown in blue. Sub-Figures (a), (b) and (c) depict histograms for the classification setting using the True-class (TC), Mondrian (Mon) and Summation (Sum) scoring functions, respectively. Sub-Figure (d) shows histograms for the regressions setting, where CP was performed using the Quantile (Qu) scoring function.

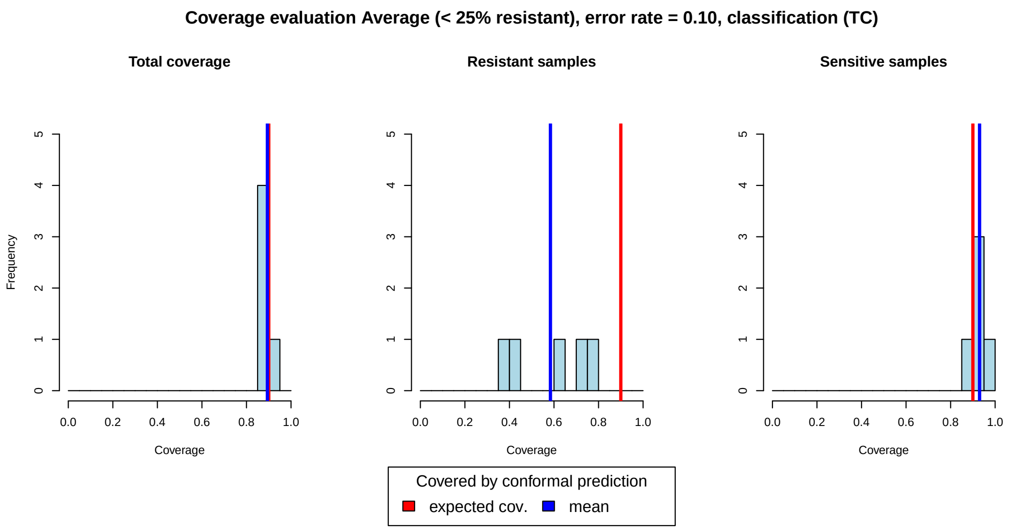

(a) True-class score

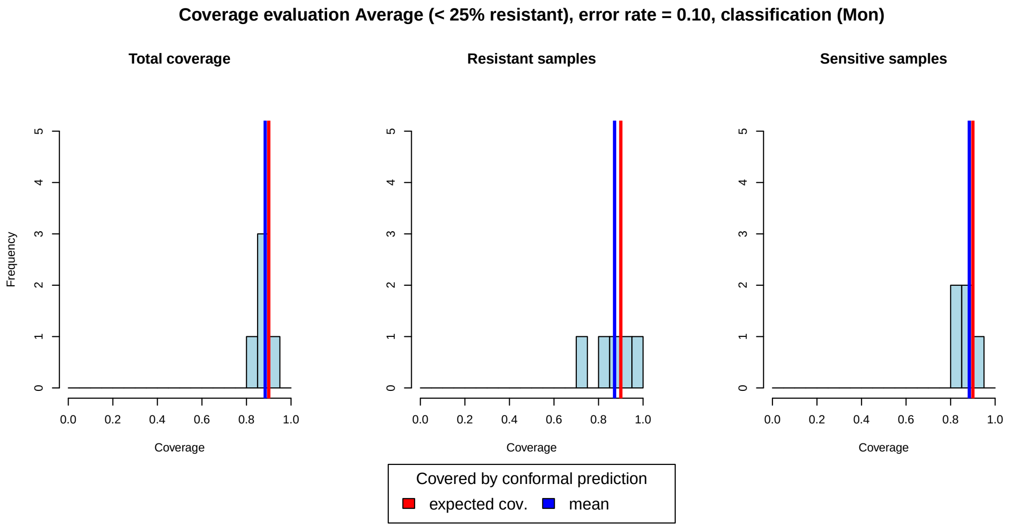

(b) Mondrian score

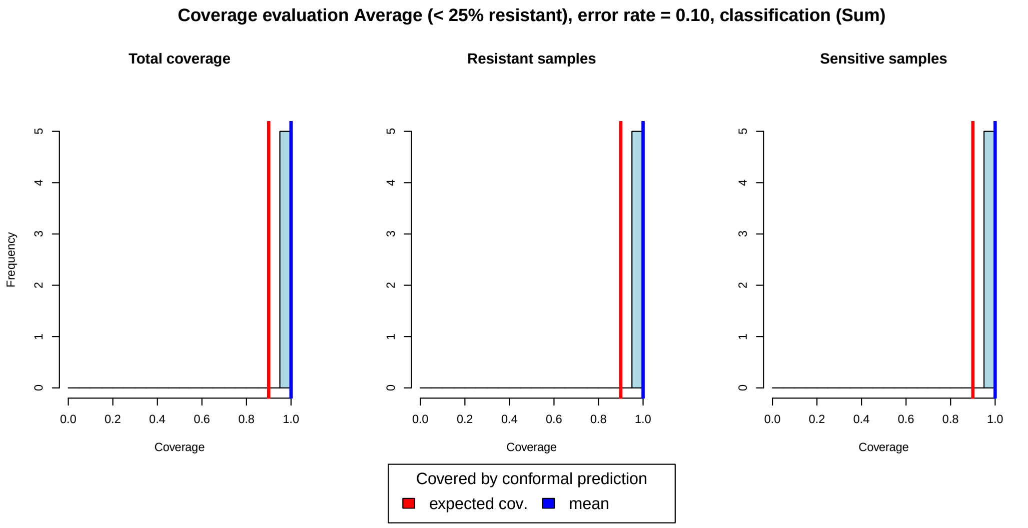

(c) Summation score

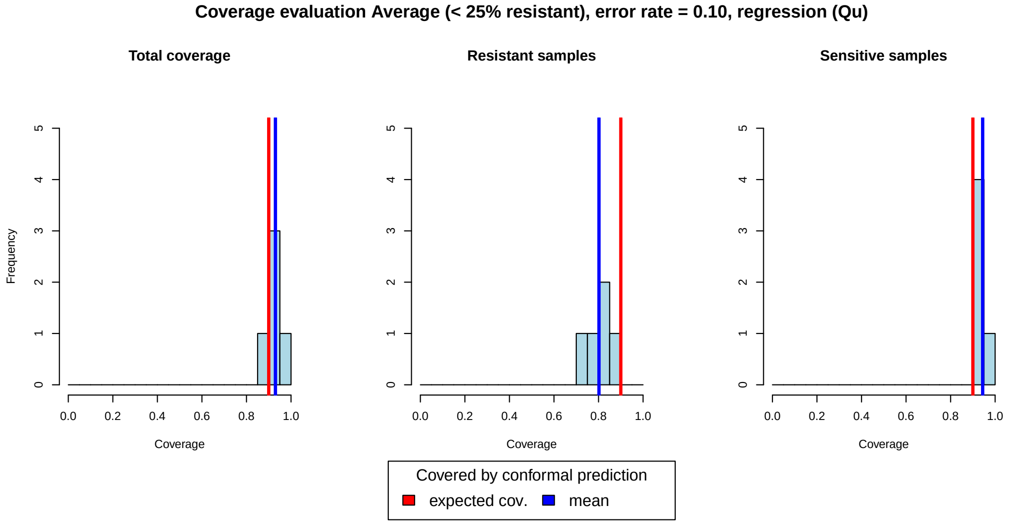

(d) Quantile score

Figure 20: **Coverage evaluation for CP models of five drugs from the GDSC2 database trained using CMax viability values and a two-class prioritization setting with underrepresentation of the resistant class.** This figure depicts histograms of the coverage property across CP models for five drugs obtained from the GDSC2 database, for which the number of resistant cell lines was less than 25%. These drugs were used to conduct the drug prioritization analyses in Section 2.4 of the main manuscript. The left plot in each sub-figure depicts the total coverage, i.e. the fraction of cell lines from the test set of each drug, for which the true response was part of the predicted set / interval. The middle and right plots show the coverage for subsets of resistant and sensitive cell lines for each drug, respectively. The expected coverage of 0.9 for the employed error rate of $\alpha = 0.1$ is shown in red, the actual mean coverage over all investigated drugs is shown in blue. Sub-Figures (a), (b) and (c) depict histograms for the classification setting using the True-class (TC), Mondrian (Mon) and Summation (Sum) scoring functions, respectively. Sub-Figure (d) shows histograms for the regressions setting, where CP was performed using the Quantile (Qu) scoring function.

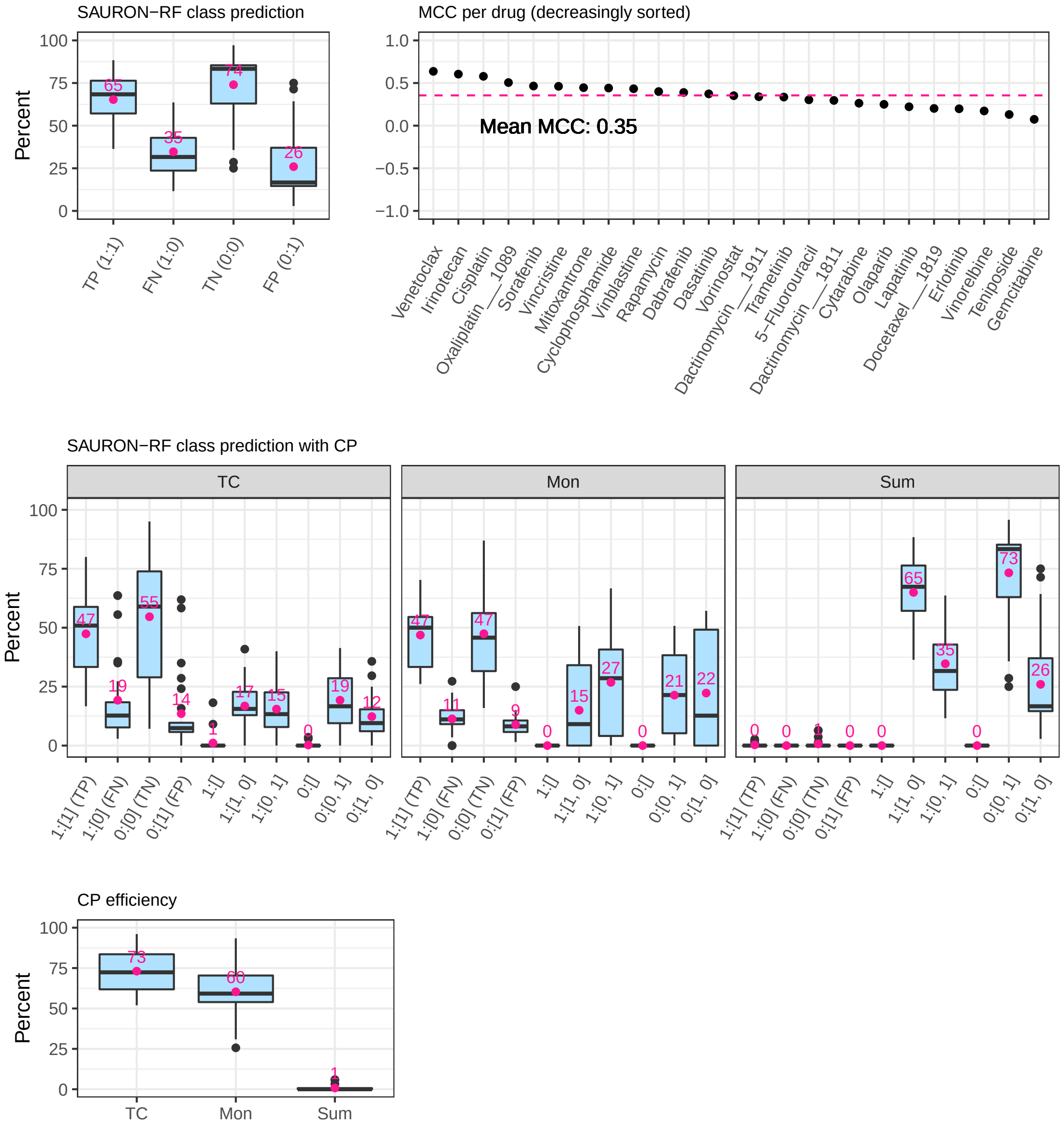

Figure 21: **Classification test set performance for 25 drugs from the GDSC2 database trained using CMax viability values and a two-class prioritization setting.** The upper row of this figure depicts the classification performance of SAURON-RF across for all GDSC2 drugs, which were used to conduct the drug prioritization analyses in Section 2.4 of the main manuscript. The middle row shows the effects of CP on the performance in terms of true positive/negative predictions. In the lower row, the CP efficiency is presented.

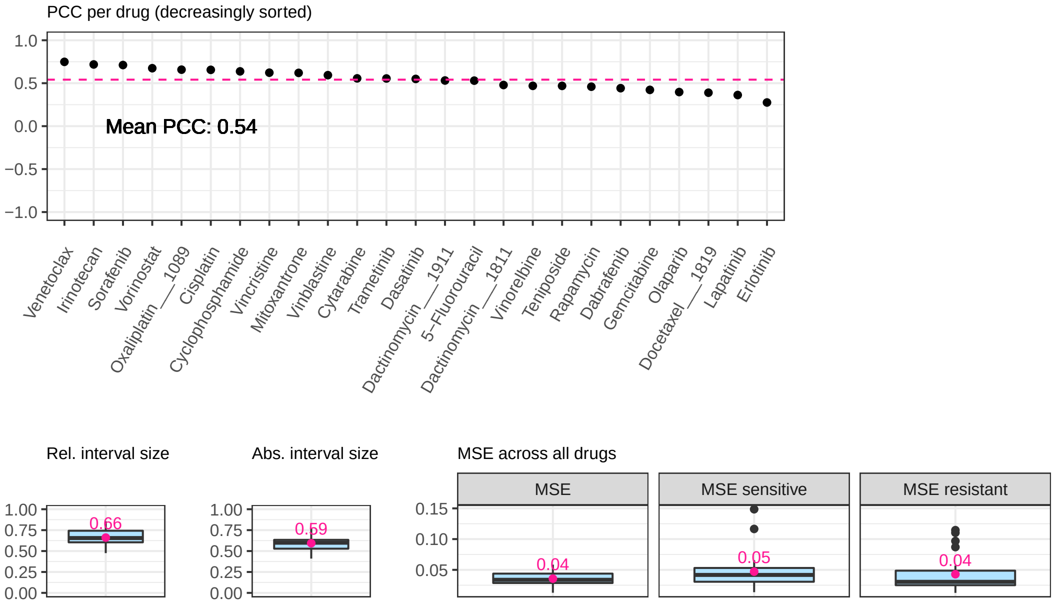

Figure 22: **Regression test set performance for 25 drugs from the GDSC2 database trained using CMax viability values and a two-class prioritization setting.** The upper row of this figure depicts the Pearson correlation coefficient between the actual continuous response values and the predicted continuous response values for all GDSC2 drugs, which were used to conduct the drug prioritization analyses in Section 2.4 of the main manuscript. The lower row shows the mean-squared error (MSE) and the interval width of the CP Quantile regression score relative to the spanned training ranges of the drugs.

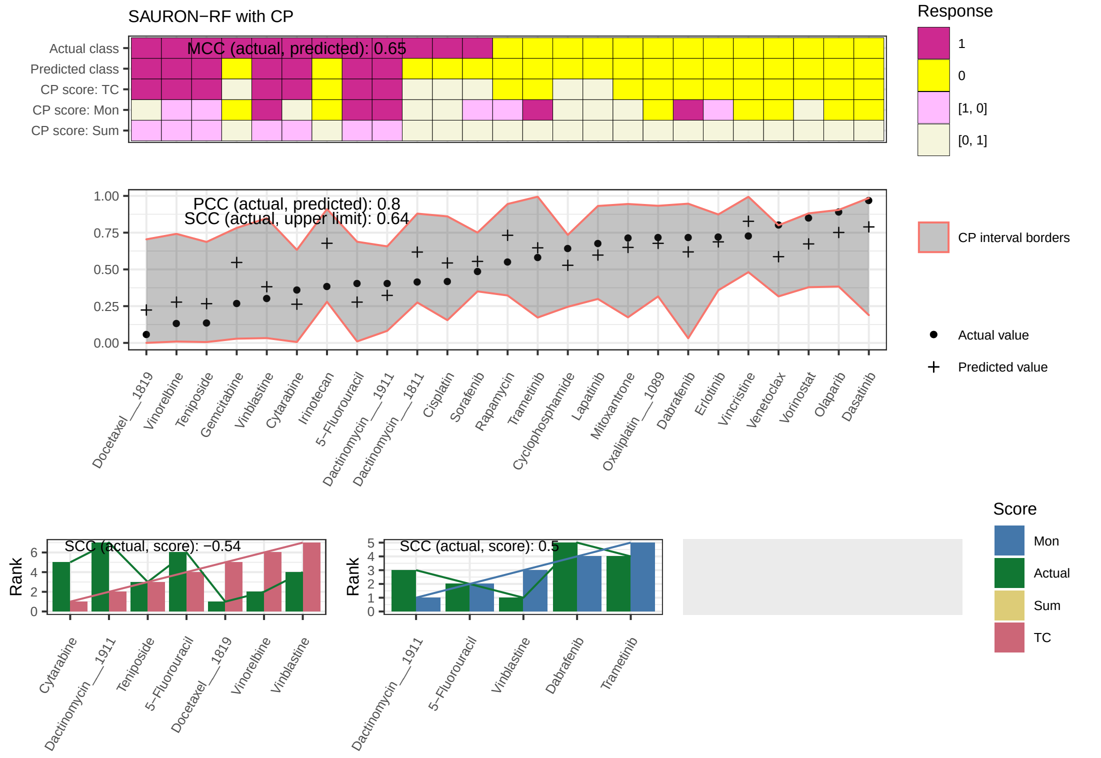

Figure 23: **Prioritization example GDSC2.**This figure exemplifies the performance of our prioritization pipeline (cf. Figure 2 in main manuscript) when applied to one particular cell line (COSMIC ID 724863) from the test set of the GDSC2 data set. The upper plot visualizes the classification performance with and without CP for all analyzed drugs. The middle plot depicts the regression result for all drugs, including the 90% CP interval, and the lower plot shows the resulting prioritized drug lists with the drugs ascendingly sorted by their upper CP limit prediction.

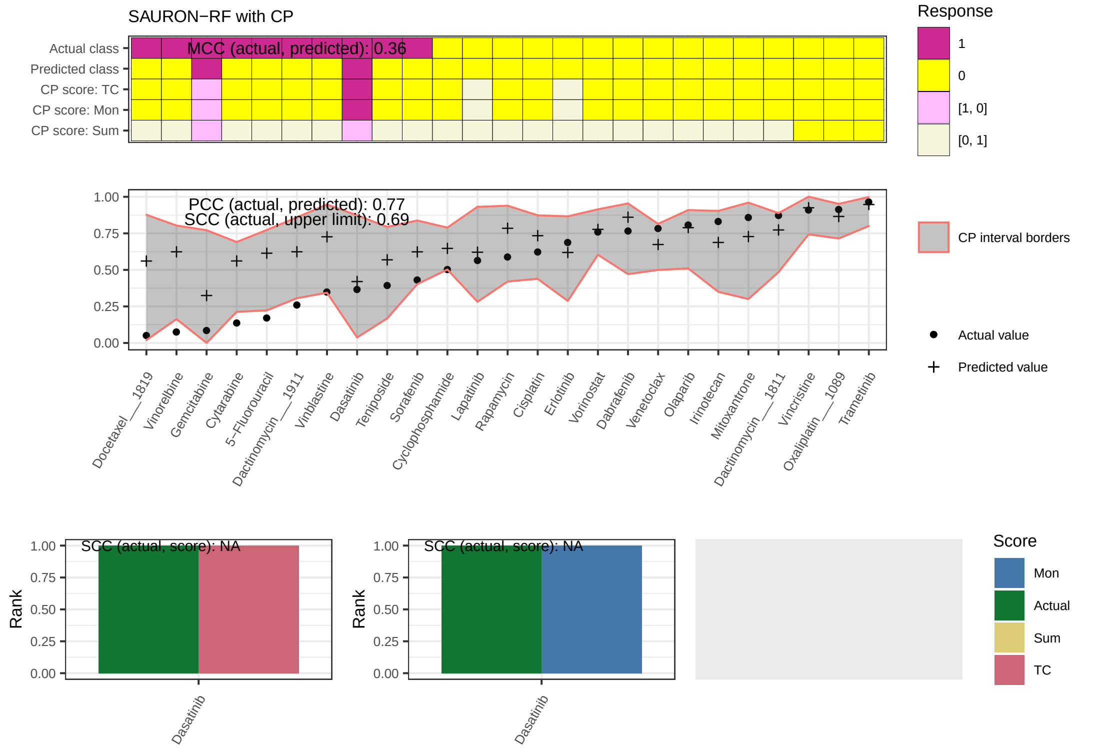

Figure 24: **Prioritization example GDSC2.**This figure exemplifies the performance of our prioritization pipeline (cf. Figure 2 in main manuscript) when applied to one particular cell line (COSMIC ID 906861) from the test set of the GDSC2 data set. The upper plot visualizes the classification performance with and without CP for all analyzed drugs. The middle plot depicts the regression result for all drugs, including the 90% CP interval, and the lower plot shows the resulting prioritized drug lists with the drugs ascendingly sorted by their upper CP limit prediction.

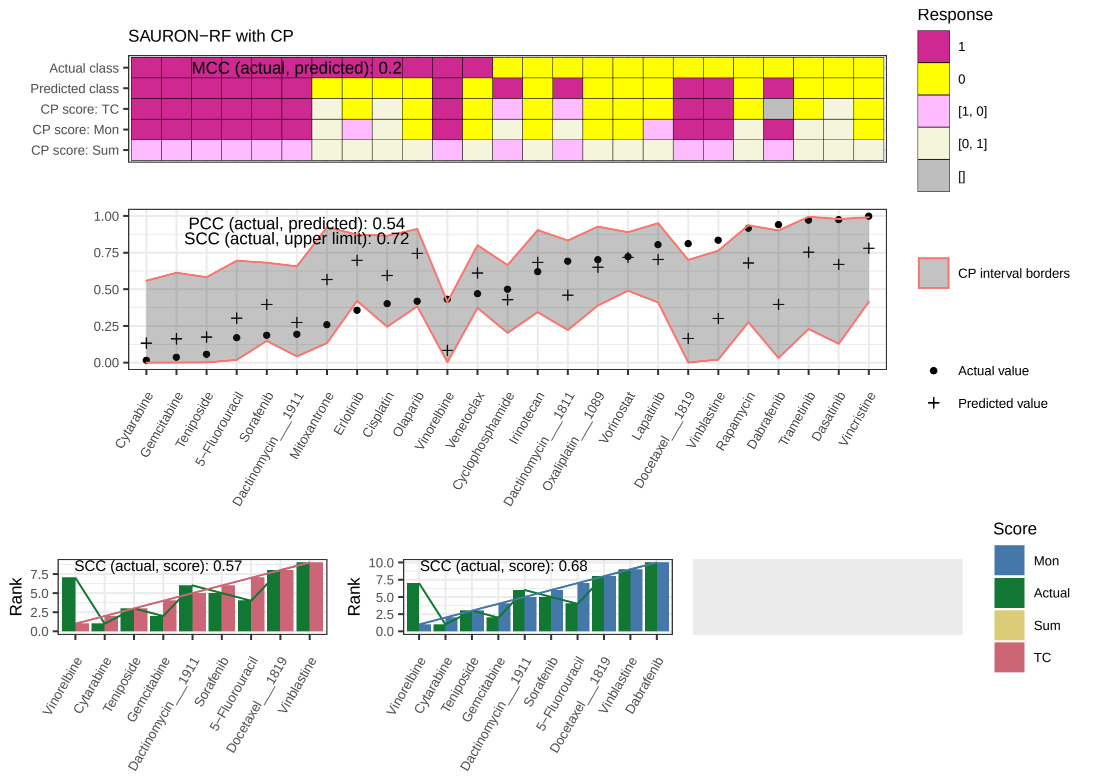

Figure 25: **Prioritization example GDSC2.**This figure exemplifies the performance of our prioritization pipeline (cf. Figure 2 in main manuscript) when applied to one particular cell line (COSMIC ID 908444) from the test set of the GDSC2 data set. The upper plot visualizes the classification performance with and without CP for all analyzed drugs. The middle plot depicts the regression result for all drugs, including the 90% CP interval, and the lower plot shows the resulting prioritized drug lists with the drugs ascendingly sorted by their upper CP limit prediction.

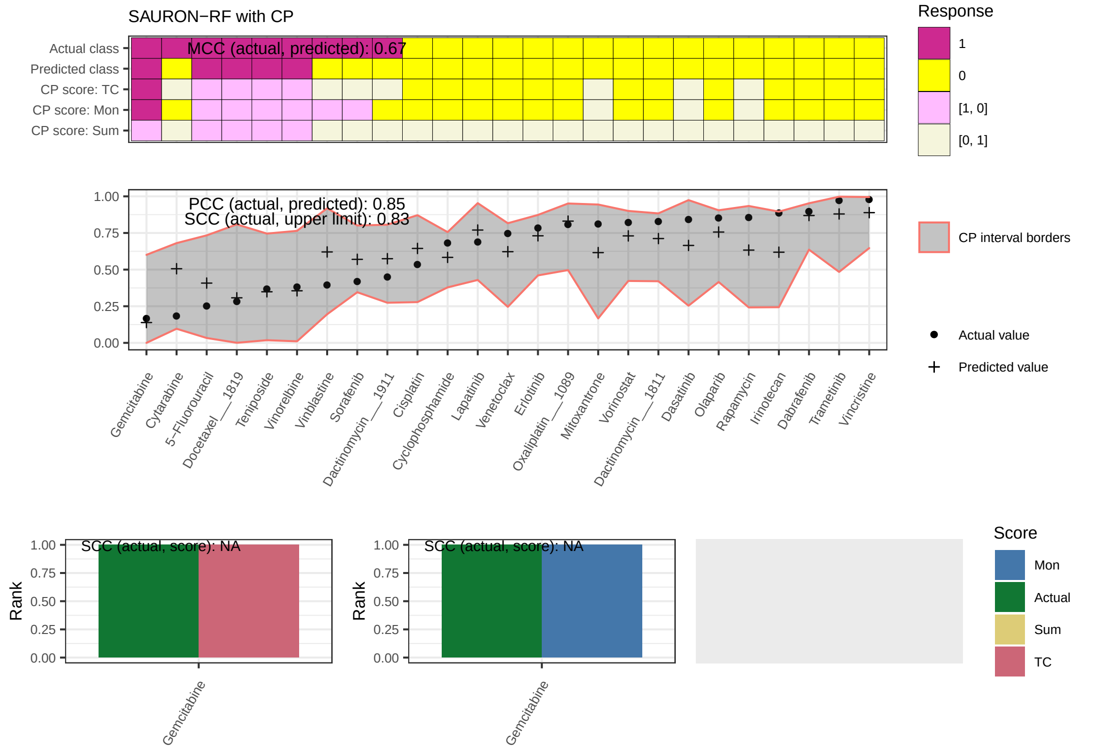

Figure 26: **Prioritization example GDSC2.**This figure exemplifies the performance of our prioritization pipeline (cf. Figure 2 in main manuscript) when applied to one particular cell line (COSMIC ID 1298216) from the test set of the GDSC2 data set. The upper plot visualizes the classification performance with and without CP for all analyzed drugs. The middle plot depicts the regression result for all drugs, including the 90% CP interval, and the lower plot shows the resulting prioritized drug lists with the drugs ascendingly sorted by their upper CP limit prediction.

5 Comparison to a multi-task multi-omics deep neural network

When we introduced SAURON-RF, we demonstrated its superiority to various drug sensitivity classification and regression approaches, including the state-of-the-art method heterogeneity-aware random forests by Rahman et al. [10] from which SAURON-RF was derived. In this manuscript, we introduce reliability guarantees and reliable drug prioritization, which have both not been described in previous work (cf. Table 1 of this supplement), i.e., there is no direct competitor to which we could compare our novel approach.

5.1 CP pipeline

However, to demonstrate the applicability of our CP pipeline to other drug sensitivity prediction methods, we decided to showcase how to apply it to a deep neural network (DNN) since DNNs enjoy relative popularity for anti-cancer drug sensitivity prediction (cf. Table 1 in this supplement). To this end, we performed an analysis with an adjusted version of the multi-task multi-omics DNN proposed by [18]. The network architecture by Chiu et al. combines a mutation autoencoder and a gene expression autoencoder with a deep neural network to predict the drug response of all drugs simultaneously.

Network architecture and hyperparameters: We have adopted the network architecture and hyperparameters as provided in the code by [18]. However, we had to adjust several (hyper)parameters to render the method amenable to our analysis setting:

- Chiu et al. performed their analyses using the CCLE RNA-seq data for the cell lines and pre-trained their expression autoencoder with the TCGA RNA-seq expression from tumours. In contrast, we use the GDSC microarray gene expression data of the cell lines in our analyses. Because of a lack of corresponding TCGA microarray data, we resorted to pre-training with the training samples instead of the tumours samples from TCGA. Note that even random initialization (without pre-training) was shown to perform well (second best approach) by Chiu et al.

- Chiu et al. performed regression with their network. We generally perform classification and regression simultaneously. Moreover, we either need a probability estimate of the classes (classification task) or quantile estimates (regression task) to apply the score functions currently implemented as part of our CP pipeline. By replacing the linear activation function in the last layer of the DNN with the sigmoidal function, we can obtain class probabilities for all drugs and consequently perform classification. Instead of the mean-squared error, we used the binary cross-entropy, a dedicated loss function for classification tasks.

Data processing: The approach by Chiu et al. requires two types of input data: binary mutation data and continuous gene expression values. As output, they employ the logarithmized IC50 values. To conduct our analyses as indicated above, we prepared these three data types as follows:

- *Mutation data:* Following the description by Chiu et al., we built a binary mutation matrix where each entry specifies whether a cell line is affected by a mutation in a gene. To generate this matrix, we employ v99 of the COSMIC cell line project (file: CellLinesProject_GenomeScreensMutant_v99_GRCh37.tsv) from which we obtained coding point mutations of the cell lines. For each gene, we add a binary vector to the mutation matrix that indicates for each cell line whether at

least one coding point mutation is present (1) or not (0). Analogous to Chiu et al., we do not consider synonymous mutations.

- *Gene expression data:* We employ the gene expression matrix as used throughout all analyses in this manuscript.
- *Drug response data:* Since we perform classification instead of regression, we had to discretize the logarithmized IC50 data. We employ the binarized IC50 data as described in the main manuscript.

After preparing these three data types, we had to ensure that the model by [18] can be applied. To this end, we had to ascertain that only cell lines with complete information for all data types (mutation, gene expression, and drug response for all drugs) are considered. Yet, not all cell lines have been screened for all drugs. Consequently, the aim is to identify the largest possible complete set of cell lines and drugs. We developed an integer linear program (ILP) to select cell lines and drugs (cf. Section 6 in this supplement). In total, we considered 600 cell lines and 170 drugs. Similar to the analyses presented in the main manuscript, we randomly split the cell lines into training (70%), calibration (15%), and test set (15%).
Results: First, we observe the same phenomenon that we already demonstrated for various approaches (including neural networks) in the SAURON-RF publication: without specific countermeasures against class or regression imbalance, the minority class (sensitive samples) is predicted poorly (cf. Figure 27 in this supplement). With CP, we can guarantee the reliability of our predictions and consequently remove false predictions (cf. Figure 28 - 30 in this supplement). It is particularly noteworthy that the class-wise calibration of the Mondrian score helps to increase the correctly identified sensitive samples (TP). In total, SAURON-RF with and without CP outperforms the approach by Chiu et al.

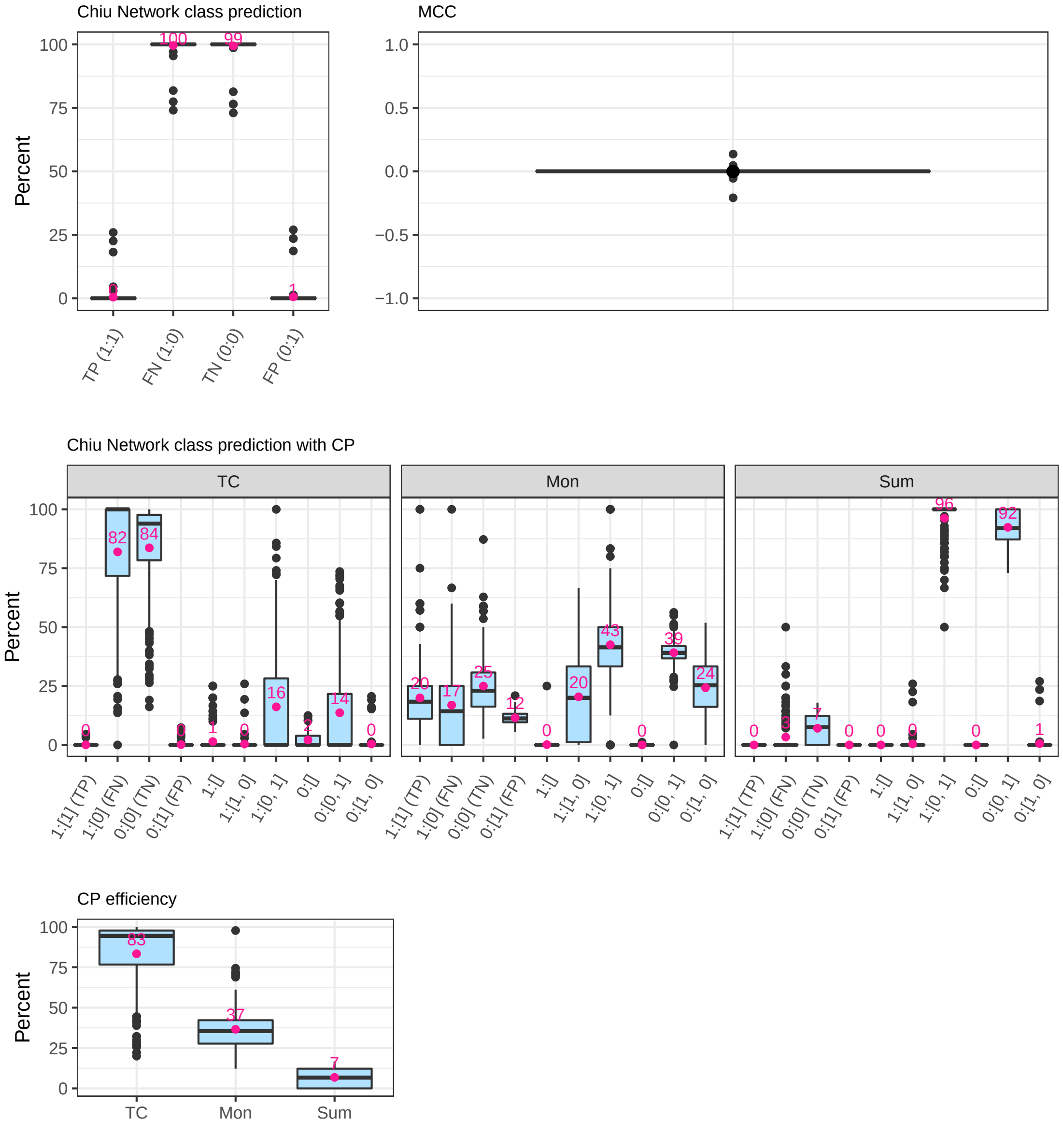

Figure 27: **Test set classification results of DNN.** This figure depicts the results of the adapted DNN by [18]. The upper row shows the classification results without CP, the middle row shows the results with the different CP score functions, and the lower row depicts the CP efficiency.

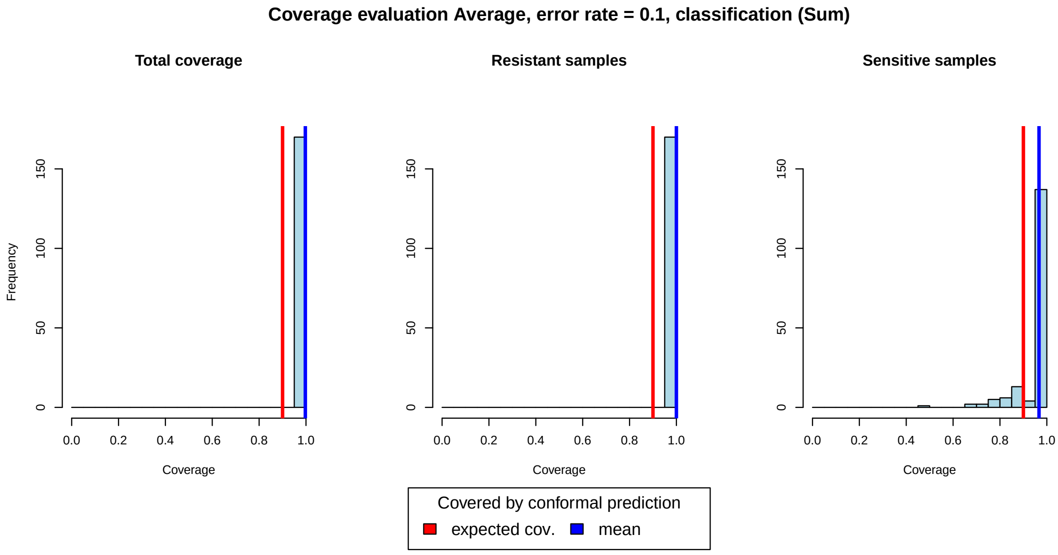

Figure 28: **Coverage of Summation score for DNN.** This figure depicts the coverage of the summation score when applied to the DNN adapted from [18]. The left plot depicts the total coverage, i.e. the fraction of cell lines from the test set of each drug, for which the true response was part of the predicted set. The middle and right plots show the coverage for subsets of resistant and sensitive cell lines for each drug, respectively. The expected coverage of 0.9 for the employed error rate of $\alpha = 0.1$ is shown in red, the actual mean coverage over all investigated drugs is shown in blue.

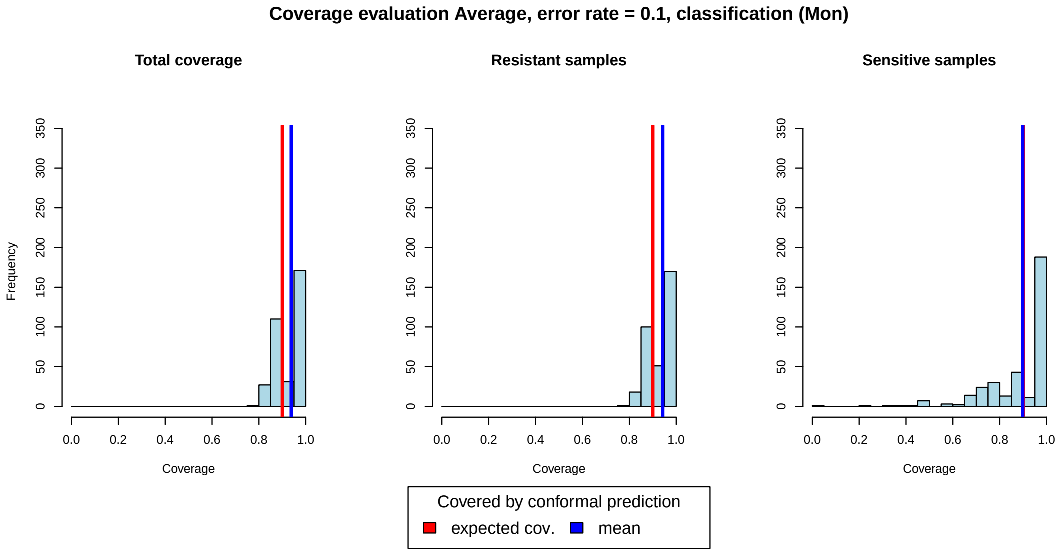

Figure 29: **Coverage of Mondrian score for DNN.** This figure depicts the coverage of the Mondrian score when applied to the DNN adapted from [18]. The left plot depicts the total coverage, i.e. the fraction of cell lines from the test set of each drug, for which the true response was part of the predicted set. The middle and right plots show the coverage for subsets of resistant and sensitive cell lines for each drug, respectively. The expected coverage of 0.9 for the employed error rate of $\alpha = 0.1$ is shown in red, the actual mean coverage over all investigated drugs is shown in blue.

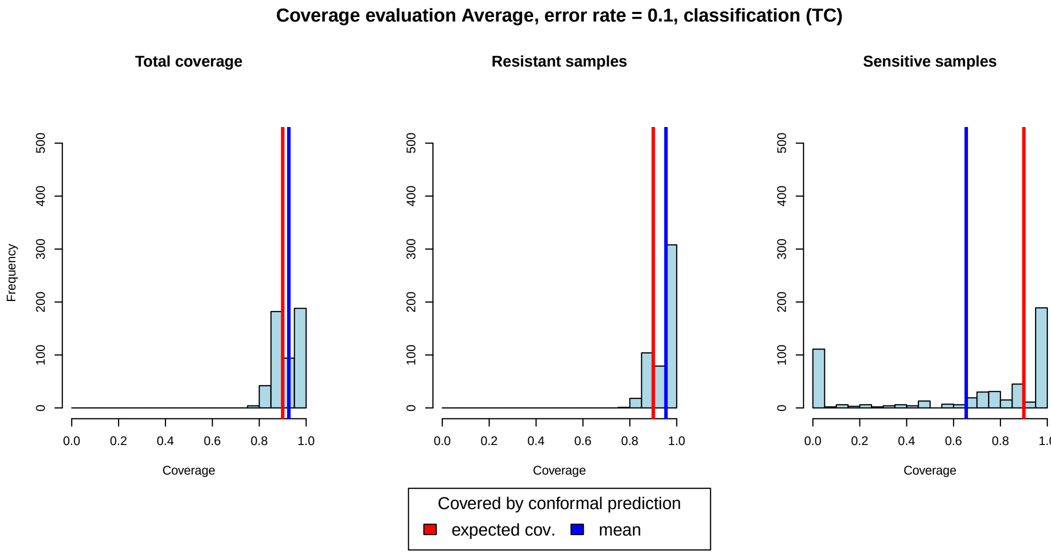

Figure 30: **Coverage of True Class score for DNN.** This figure depicts the coverage of the True Class score when applied to the DNN adapted from [18]. The left plot depicts the total coverage, i.e. the fraction of cell lines from the test set of each drug, for which the true response was part of the predicted set. The middle and right plots show the coverage for subsets of resistant and sensitive cell lines for each drug, respectively. The expected coverage of 0.9 for the employed error rate of $\alpha = 0.1$ is shown in red, the actual mean coverage over all investigated drugs is shown in blue.

5.2 CMax viability

In addition to the CP pipeline, we devised a novel drug sensitivity measure with across-drug comparability, the CMax viability. Generally, the high correlation between IC50 and CMax viability (cf. Figures 7 and 8 in this supplement) suggests that we can expect this novel measure to be predicted similarly well as established measures such as the IC50 value. Our analyses (with IC50 compared to CMax viability) with reliable SAURON-RF confirm this expectation. In order to provide a comparison with a second method, we once again performed an analysis with an adapted version of the multi-task multi-omics DNN by [18].

Network architecture and hyperparameters: In general, we adopted the network architecture and hyperparameters as described in Section 5.1. However, since we perform regression, we could use the original activation and loss function of the DNN by [18].

Data processing: Except for the drug response data, we prepared the data sets as described in Section 5.1. As drug response, we now employ the CMax viability, which is available for 47 drugs from GDSC2. By intersecting these 47 drugs with the 170 drugs yielded from the ILP, we finally obtained 42 drugs for which we could perform the analyses. We split the cell lines into a training (70 %) and test set (30 %).

Results: The overall achieved test MSE is similar to that of SAURON-RF (Chiu: 0.09, SAURON:RF: 0.03, cf. Figure 31 in this Supplement). However, SAURON-RF consistently achieves lower MSEs. Moreover, the correlation results (mean PCC Chiu: 0, mean SCC Chiu: 0.02, cf. Figure 32 in this supplement) imply that the approach by Chiu et al. is not able to sort the cell lines per drug. In contrast, Reliable SAURON-RF achieves a decent sorting (mean PCC SAURON-RF: 0.51, cf. Figure 14 in this supplement).

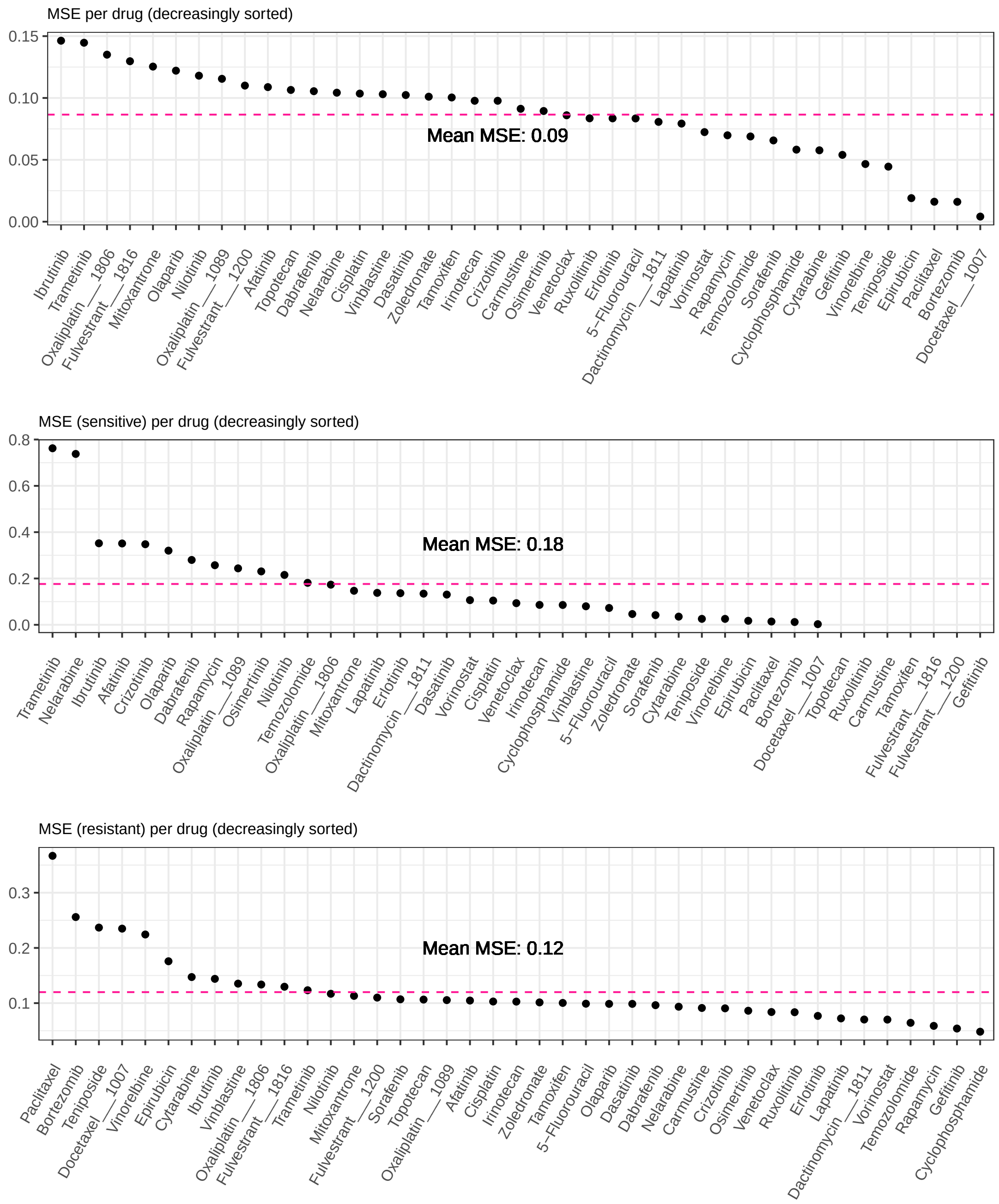

Figure 31: **Test MSE of DNN for prediction of CMax viabilities**. This figure depicts the MSE of the approach by Chiu et al. [18] when applied to 42 drugs with available CMax viabilities. The first row shows the overall test set MSE. The middle row depicts the MSE for all cell lines sensitive to the respective drug and the last row shows the MSE for the resistant cell lines.

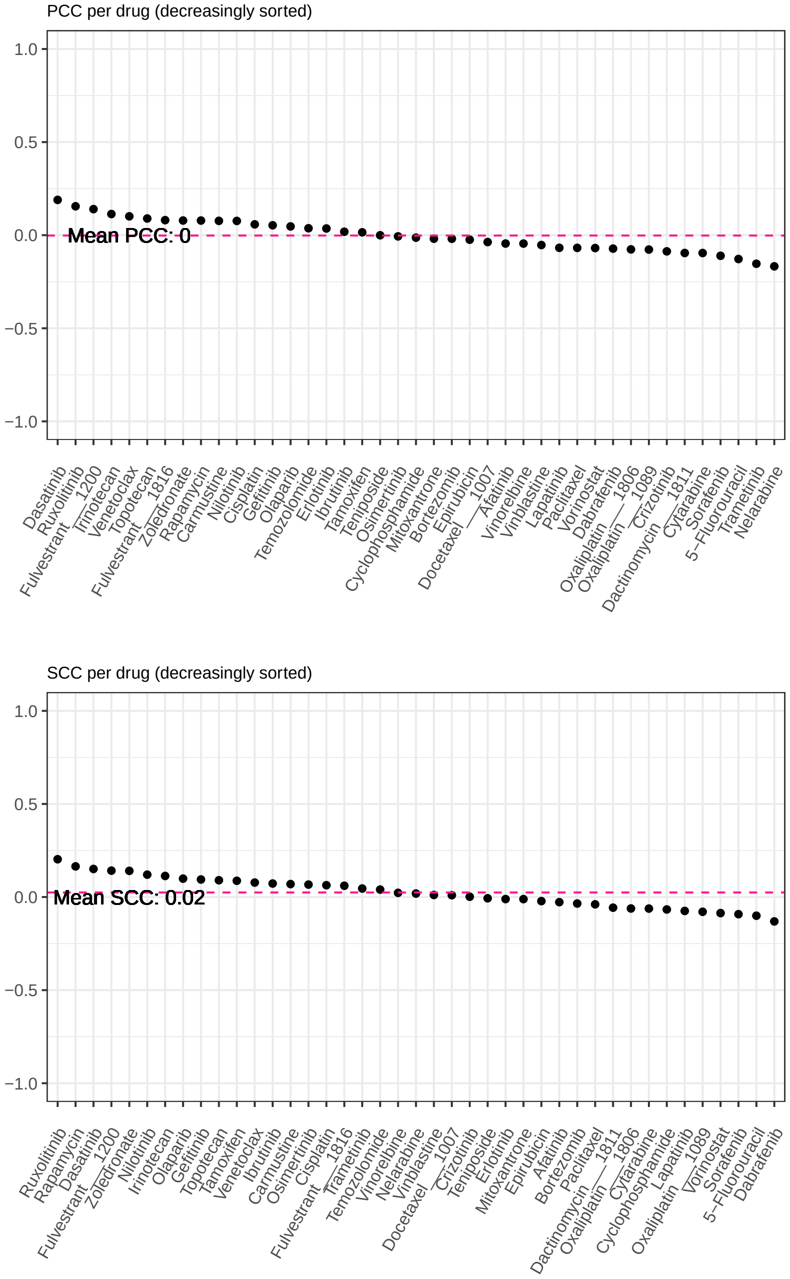

Figure 32: **Test PCC and SCC of DNN**. This figure shows the PCC and SCC results for the approach by Chiu et al. [18] applied to the 42 drugs with available CMax viabilities. The first plot shows the PCC per drug and the second one depicts the SCC per drug.

6 Drug and cell line selection integer linear program

To determine the maximal subsets of cell lines and drugs for which complete information on all used data types is available, we formulated an integer linear program (ILP) that we present in the following.
Let N be the number of cell lines and M be the number of drugs. Let $A \in \{0,1\}^{N \times M}$ denote the binary matrix that indicates whether the available data is complete for a particular cell line-drug pair, i.e., an entry in this matrix is 1 if the drug has been screened for the cell line and 0 otherwise. Cell lines with missing copy number or mutation data were already excluded from analysis before generating A. Moreover, let

$$x_i = \begin{cases} 1, & \text{if cell line is selected} \\ 0, & \text{otherwise} \end{cases} , \forall i \in \{1, \ldots, N\} \tag{1}$$

and

$$y_j = \begin{cases} 1, & \text{if drug is selected} \\ 0, & \text{otherwise} \end{cases} , \forall j \in \{1, \ldots, M\} \tag{2}$$

be the selection variables for all cell lines and drugs, respectively. Then, we aim to maximize the number of selected drugs and cell lines, i.e., the objective function of the ILP becomes

$$\text{maximize} \quad \lambda \cdot (\sum_{j=1}^{M} y_j) + (1 - \lambda) \cdot (\sum_{i=1}^{N} x_i) \tag{3}$$

with $\lambda \in [0,1]$ representing a weight factor that balances between cell lines and drugs. To ensure that cell lines and drugs without screening are omitted from the analysis, we add the following two constraints

$$\forall i \in \{1, \ldots, N\} : x_i \leq \sum_{j=1}^{M} A_{ij} \tag{4}$$

and

$$\forall j \in \{1, \ldots, M\} : y_j \leq \sum_{i=1}^{N} A_{ij}. \tag{5}$$

Furthermore, we cannot select a cell line i if no screening has been performed for one of the selected drugs (i.e., $A_{ij} = 0$ for at least one selected j). The corresponding constraint takes the form

$$\forall i \in \{1, \ldots, N\} : M \cdot x_i \leq (\sum_{\forall j : A_{ij} = 1} 1) + (\sum_{\forall j : A_{ij} = 0} 1 - y_j). \tag{6}$$

In contrast, we have to select a cell line i if, for all selected drugs, a screening has been performed, i.e., we obtain the constraint

$$\forall i \in \{1, \ldots, N\} : (\sum_{j=1}^{M} y_j) - (\sum_{j=1}^{M} A_{ij} \cdot y_j) + x_i \geq 1. \tag{7}$$

To ensure that a minimal number of drugs ($\mathrm{Min}_{\mathrm{drugs}}$) and a minimal number of cell lines ($\mathrm{Min}_{\mathrm{samples}}$) are used, we add the following two constraints:

$$\sum_{j=1}^{M} y_j \geq \mathrm{Min}_{\mathrm{drugs}} \tag{8}$$

and

$$\sum_{i=1}^{N} x_i \geq \mathrm{Min}_{\mathrm{samples}}. \tag{9}$$

Our results (170 drugs and 600 cell lines) were obtained with the following parameters:

- $\lambda = 1$, i.e., we maximize the number of drugs
- $\mathrm{Min}_{\mathrm{samples}} = 600$, i.e., we expect that at least 600 cell lines must be chosen
- $\mathrm{Min}_{\mathrm{drugs}} = 1$ (default setting)

7 Amendments and explanations to figures specifying classification performance

In our figures, we gave the classification performance in percent. In the following, we provide all formulas for the two-class case, for the three-class case they can be derived analogously. Firstly, we define several auxiliary variables:

$$
\begin{aligned}
A &:= \#\text{sensitive samples with empty class prediction}\\
B &:= \#\text{sensitive samples with [1,0] as class prediction}\\
C &:= \#\text{sensitive samples with [0,1] as class prediction}\\
D &:= \#\text{resistant samples with empty class prediction}\\
E &:= \#\text{resistant samples with [1,0] as class prediction}\\
F &:= \#\text{resistant samples with [0,1] as class prediction}
\end{aligned}
$$

Using these auxiliary variables, the classification performance was quantified as follows:

$$
\begin{aligned}
\text{percent TP, case 1:1 (equal to sensitivity)} &= \frac{TP}{TP+FN+A+B+C}\\
\text{percent FN, case 1:0 (equal to miss-rate)} &= \frac{FN}{TP+FN+A+B+C}\\
\text{percent TN, case 0:0 (equal to specificity)} &= \frac{TN}{TN+FP+D+E+F}\\
\text{percent FP, case 0:1 (equal to fall-out)} &= \frac{FP}{TN+FP+D+E+F}\\
\text{case } 1:[] &= \frac{\#\text{sensitive samples with empty class prediction}}{TP+FN+A+B+C}\\
\text{case } 1:[1,0] &= \frac{\#\text{sensitive samples with [1,0] as class prediction}}{TP+FN+A+B+C}\\
\text{case } 1:[0,1] &= \frac{\#\text{sensitive samples with [0,1] as class prediction}}{TP+FN+A+B+C}\\
\text{case } 0:[] &= \frac{\#\text{resistant samples with empty class prediction}}{TN+FP+D+E+F}\\
\text{case } 0:[1,0] &= \frac{\#\text{resistant samples with [1,0] as class prediction}}{TN+FP+D+E+F}\\
\text{case } 0:[0,1] &= \frac{\#\text{resistant samples with [0,1] as class prediction}}{TN+FP+D+E+F}
\end{aligned}
$$

References

[1] Lightfoot, H., et al. "gdscIC50: Pipeline for GDSC Curve Fitting" R package version 0.99.4 (2021)

[2] Vis, D. J., et al. "Multilevel models improve precision and speed of IC50 estimates" Pharmacogenomics 17.7 (2016): 691 – 700. https://doi.org/10.2217/pgs.16.15

[3] Vovk, V. "Conditional validity of inductive conformal predictors" Asian conference on machine learning (2012): 475 – 490.

[4] Angelopoulos, A. N. and Bates, S. "A gentle introduction to conformal prediction and distribution-free uncertainty quantification" arXiv preprint (2021). arXiv:2107.07511 https://doi.org/10.48550/arXiv.2107.07511

[5] Menden, M. P. and Iorio, F. and Garnett, M. and McDermott, U. and Benes, C. H. and Ballester, P. J. and Saez-Rodriguez, J. "Machine Learning Prediction of Cancer Cell Sensitivity to Drugs Based on Genomic and Chemical Properties" PLOS ONE 8.4 (2013): e61318. https://doi.org/10.1371/journal.pone.0061318

[6] Zhang, N. and Wang, H. and Fang, Y. and Wang, J. and Zheng, X. and Liu, X. S. "Predicting anticancer drug responses using a dual-layer integrated cell line-drug network model" PLoS computational biology 11.9 (2015): e1004498. https://doi.org/10.1371/journal.pcbi.1004498

[7] Knijnenburg, T. A. and Klau, G. W. and Iorio, F. and Garnett, M. J. and McDermott, U. and Shmulevich, I. and Wessels, L. F. A. "Logic models to predict continuous outputs based on binary inputs with an application to personalized cancer therapy" Scientific reports 6 (2016): 36812. https://doi.org/10.1101/036970

[8] Stanfield, Z. and Coşkun, M. and Koyutürk, M. "Drug response prediction as a link prediction problem" Scientific reports 7 (2017): 40321. https://doi.org/10.1038/srep40321

[9] Wang, L. and Li, X. and Zhang, L. and Gao, Q. "Improved anticancer drug response prediction in cell lines using matrix factorization with similarity regularization" BMC Cancer 17 (2017): 513. https://doi.org/10.1186/s12885-017-3500-5

[10] Rahman, R. and Matlock, K. and Ghosh, S. and Pal, R. "Heterogeneity aware random forest for drug sensitivity prediction" Scientific reports 7 (2017): 11347. https://doi.org/10.1038/s41598-017-11665-4

[11] Zhang, F. and Wang, M. and Xi, J. and Yang, J. and Li, A. "A novel heterogeneous network-based method for drug response prediction in cancer cell lines" Scientific reports 8 (2018): 3355. https://doi.org/10.1038/s41598-018-21622-4

[12] Matlock, K. and De Niz, C. and Rahman, R. and Ghosh, S. and Pal, R "Investigation of model stacking for drug sensitivity prediction" BMC Bioinformatics 19.3 (2018): 71. https://doi.org/10.1186/s12859-018-2060-2

[13] He, X. and Folkman, L. and Borgwardt, K. " Kernelized rank learning for personalized drug recommendation" Bioinformatics 34.16 (2018): 2808 – 2816. https://doi.org/10.1093/bioinformatics/bty132

[14] Basu, A. and Mitra, R: and Liu, H. and Schreiber, S. L. and Clemons, P. A. "RWEN: response-weighted elastic net for prediction of chemosensitivity of cancer cell lines" Bioinformatics 34.19 (2018): 3332 – 3339. https://doi.org/10.1093/bioinformatics/bty199

[15] Chang, Y. and Park, H. and Yang, H.-J. and Lee, S. and Lee, K.-Y. and Kim, T. S. and Jung, J. and Shin, J.-M. "Cancer drug response profile scan (CDRscan): a deep learning model that predicts drug effectiveness from cancer genomic signature" Scientific reports 8 (2018): 8857. https://doi.org/10.1038/s41598-018-27214-6

[16] Fang, Y. and Xu, P. and Yang, J. and Qin, Y. "A quantile regression forest based method to predict drug response and assess prediction reliability" PLOS ONE 13.10 (2018): e0205155. https://doi.org/10.1371/journal.pone.0205155

[17] Liu, H. and Zhao, Y. and Zhang, L: and Chen X. "Anti-cancer Drug Response Prediction Using Neighbor-Based Collaborative Filtering with Global Effect Removal" Molecular Therapy-Nucleic Acids 13 (2018): 303 – 311. https://doi.org/10.1016/j.omtn.2018.09.011

[18] Chiu, Y.-C. and Chen, H.-I. H. and Zhang, T. and Zhang, S. and Gorthi, A. and Wang, L.-J. and Huang, Y. and Chen, Y. "Predicting drug response of tumors from integrated genomic profiles by deep neural networks" BMC medical genomics 12.1 (2019): 143 – 155. https://doi.org/10.1186/s12920-018-0460-9

[19] Su, R. and Liu, X. and Wei, L. and Zou, Q. "Deep-Resp-Forest: a deep forest model to predict anti-cancer drug response" Methods 166 (2019): 91 – 102. https://doi.org/10.1016/j.ymeth.2019.02.009

[20] Rampášek, L. and Hidru, D. and Smirnov, P. and Haibe-Kains, B. and Goldenberg, A. "Dr. VAE: improving drug response prediction via modeling of drug perturbation effects" Bioinformatics 35.19 (2019): 3743–3751. https://doi.org/10.1093/bioinformatics/btz158

[21] Oskooei, A. and Manica, M. and Mathis, R. and Martínez, M. R. "Network-based biased tree ensembles (NetBiTE) for drug sensitivity prediction and drug sensitivity biomarker identification in cancer" Scientific reports 9 (2019): 15918. https://doi.org/10.1038/s41598-019-52093-w

[22] Deng, L. and Cai, Y. and Zhang, W. and Yang, W. and Gao, B. and Liu, H. "Pathway-Guided Deep Neural Network toward Interpretable and Predictive Modeling of Drug Sensitivity" Journal of Chemical Information and Modeling 60.10 (2020): 4497 – 4505. https://doi.org/10.1021/acs.jcim.0c00331

[23] Tang, Y.-C. and Gottlieb, A. "Explainable drug sensitivity prediction through cancer pathway enrichment" Scientific reports 11 (2021): 3128. https://doi.org/10.1038/s41598-021-82612-7

[24] Lenhof, K. and Gerstner, N. and Kehl, T. and Eckhart, L. and Schneider, L. and Lenhof, H.-P. "MERIDA: a novel Boolean logic-based integer linear program for personalized cancer therapy" Bioinformatics 37.21 (2021): 3881 – 3888. https://doi.org/10.1093/bioinformatics/btab546

[25] Nguyen, T. and Nguyen, G. T. T. and Nguyen, T. and Le D.-H. "Graph Convolutional Networks for Drug Response Prediction" IEEE/ACM transactions on computational biology and bioinformatics 19.1 (2022): 146 – 154. https://doi.org/10.1101/2020.04.07.030908

[26] Liu, M. and Shen, X. and Pan, W. "Deep reinforcement learning for personalized treatment recommendation" Statistics in medicine 41.20 (2022): 4034 – 4056 https://doi.org/10.1002/sim.9491

[27] Lenhof, K. and Eckhart, L. and Gerstner, N. and Kehl, T. and Lenhof, H.-P. "Simultaneous regression and classification for drug sensitivity prediction using an advanced random forest method" Scientific reports 12 (2022):13458 https://doi.org/10.1038/s41598-022-17609-x

[28] Liston, D. R. and Davis, M. "Clinically Relevant Concentrations of Anticancer Drugs: A Guide for Nonclinical StudiesGuide to Clinical Exposures of Anticancer Drugs" Clinical cancer research 23.14 (2017): 3489 – 3498 https://doi.org/10.1158/1078-0432.ccr-16-3083

[29] Ahmadi Moughari, M. and Eslahchi, C. "ADRML: anticancer drug response prediction using manifold learning" Scientific reports 10.1 (2020): 14245 https://doi.org/10.1038/s41598-020-71257-7

[30] Meybodi, F. Y. and Eslahchi, C. "Predicting anti-cancer drug response by finding optimal subset of drugs" Bioinformatics 37.23 (2021): 4509 -– 4516 https://doi.org/10.1093/bioinformatics/btab466
