# Supplementary material for: Reliable anti-cancer drug sensitivity prediction and prioritization
Source: Sci Rep. 2024 May 29;14:12303. doi: 10.1038/s41598-024-62956-6 (PMC11137046; doi:10.1038/s41598-024-62956-6)
Supplement: Supplementary file 2 — Supplementary Information 2. [file 41598_2024_62956_MOESM2_ESM.pdf]

# - Supplement 2 -
Reliable Anti-Cancer Drug Sensitivity Prediction and Prioritization

Kerstin Lenhof, Lea Eckhart, Lisa-Marie Rolli, Andrea Volkamer, and Hans-Peter Lenhof

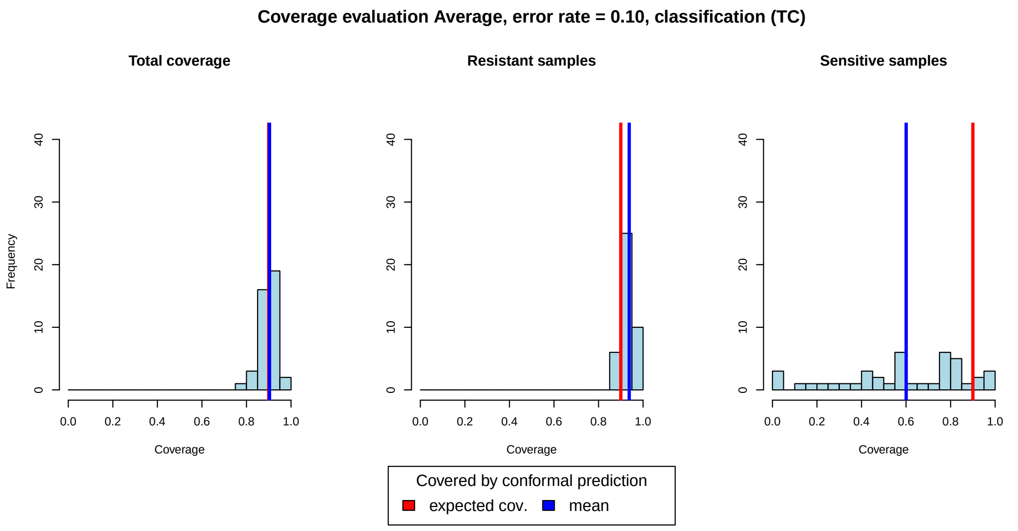

(a) True-class score

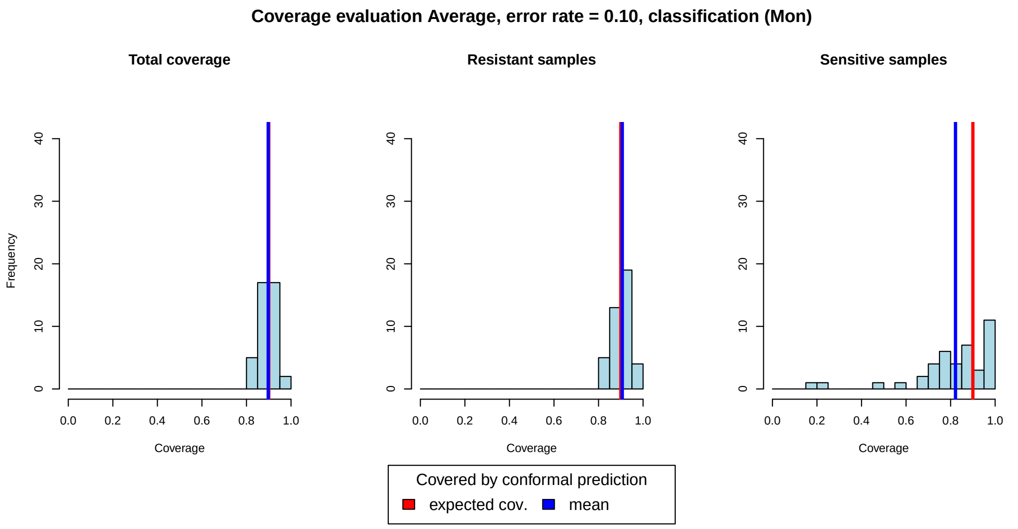

(b) Mondrian score

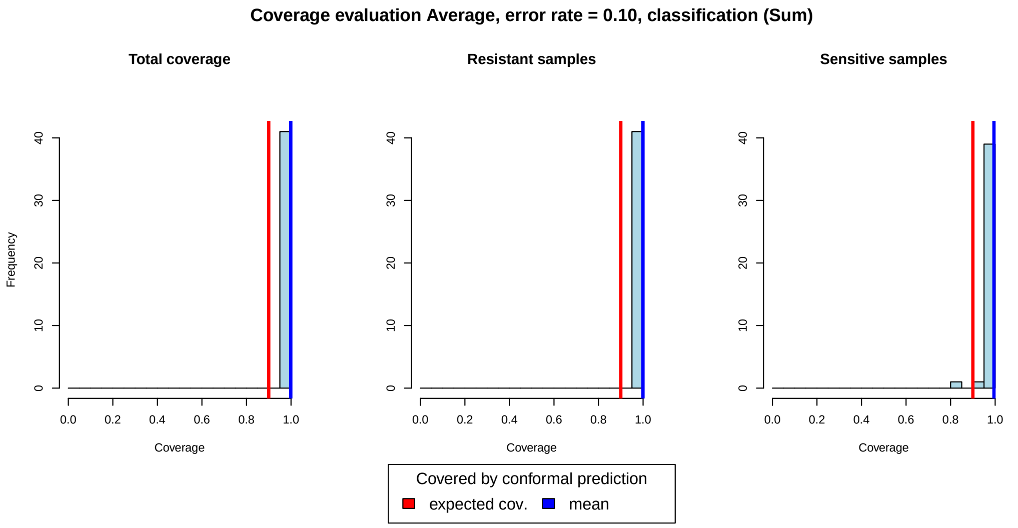

(c) Summation score

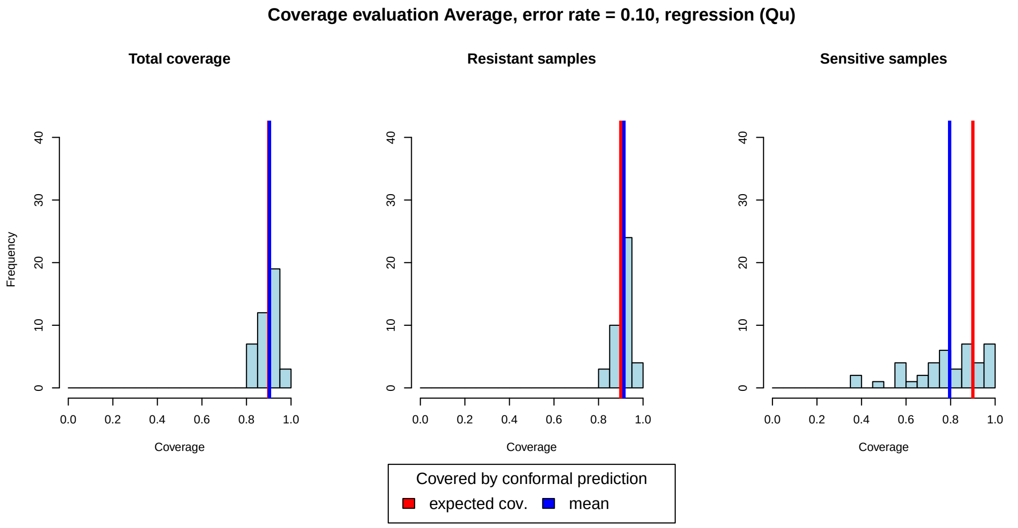

(d) Quantile score

Figure 1: **Coverage evaluation for CP models of 41 drugs from the GDSC1 database trained using IC50 values and a two-class classification setting.** This figure depicts histograms of the coverage property across CP models for 41 drugs obtained from the GDSC1 database. The left plot in each sub-figure depicts the total coverage, i.e. the fraction of cell lines from the test set of each drug, for which the true response was part of the predicted set / interval. The middle and right plots show the coverage for subsets of resistant and sensitive cell lines for each drug, respectively. The expected coverage of 0.9 for the employed error rate of $\alpha = 0.1$ is shown in red, the actual mean coverage over all investigated drugs is shown in blue. Sub-Figures (a), (b) and (c) depict histograms for the classification setting using the True-class (TC), Mondrian (Mon) and Summation (Sum) scoring functions, respectively. Sub-Figure (d) shows histograms for the regressions setting, where CP was performed using the Quantile (Qu) scoring function.

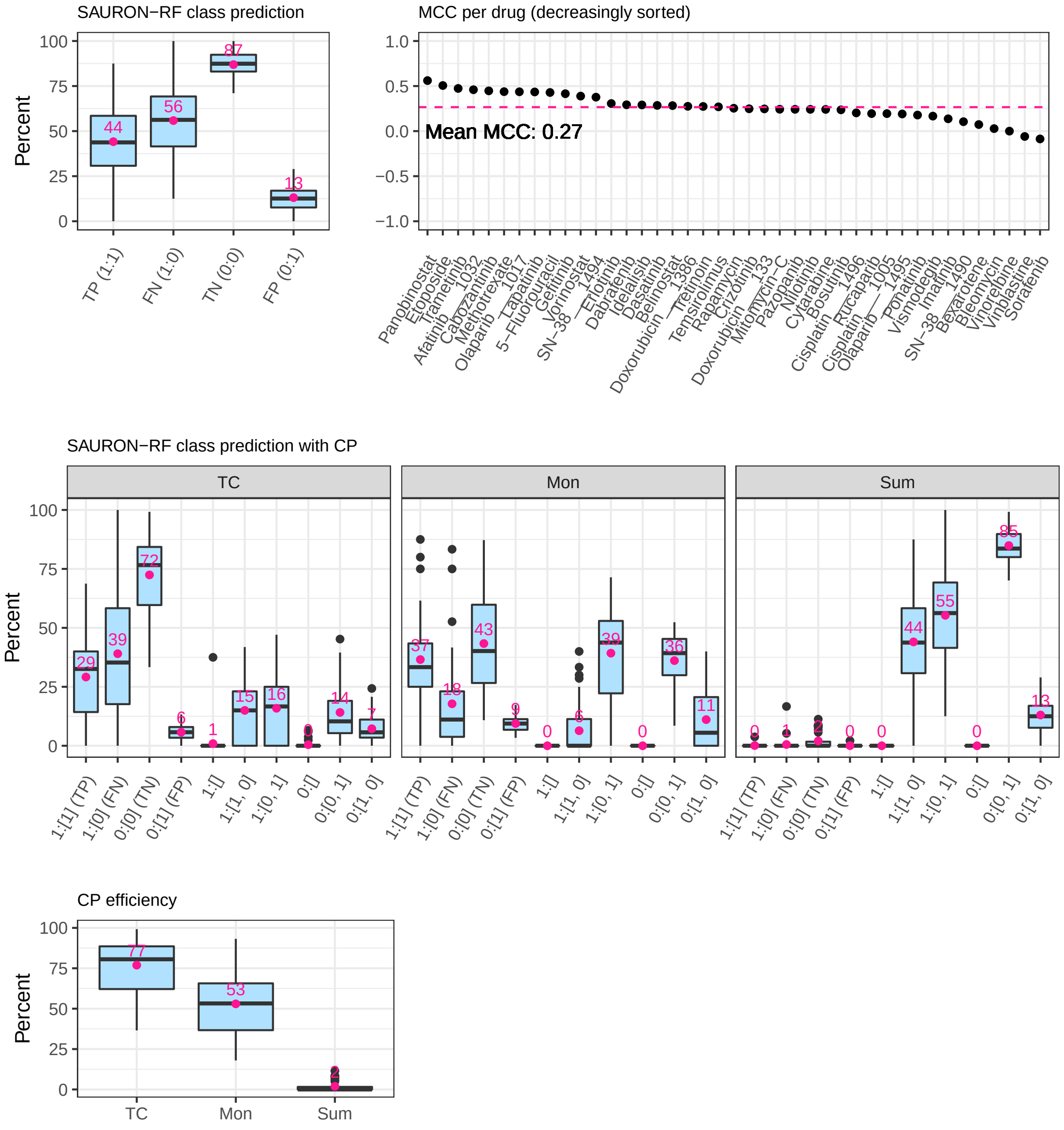

Figure 2: **Classification test set performance for 41 drugs from the GDSC1 database trained using IC50 values and a two-class classification setting.** The upper row of this figure depicts the classification performance of SAURON-RF across the different drugs. The middle row shows the effects of CP on the performance in terms of true positive/negative predictions. In the lower row, the CP efficiency is presented.

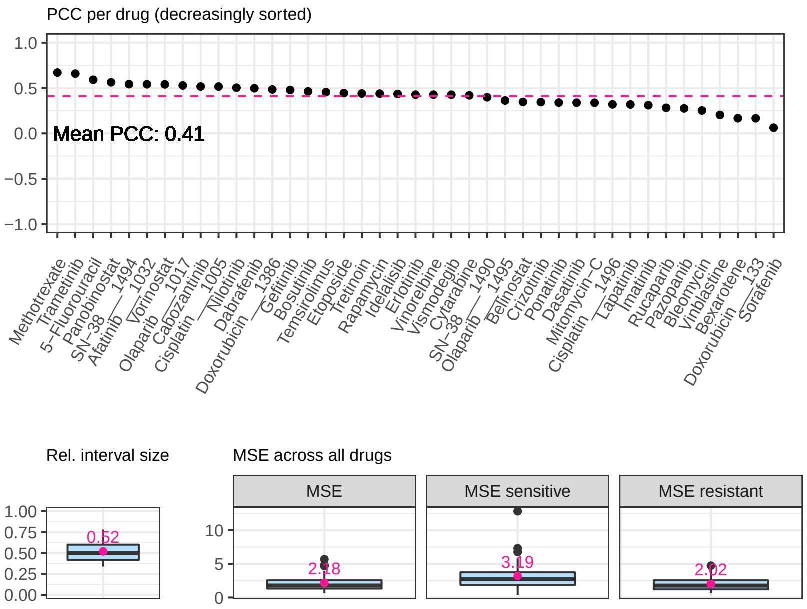

Figure 3: **Regression test set performance for 41 drugs from the GDSC1 database trained using IC50 values and a two-class classification setting.** The upper row of this figure depicts the Pearson correlation coefficient between the actual continuous response values and the predicted continuous response values for all drugs. The lower row shows the mean-squared error (MSE) and the interval width of the CP Quantile regression score relative to the spanned training ranges of the drugs.

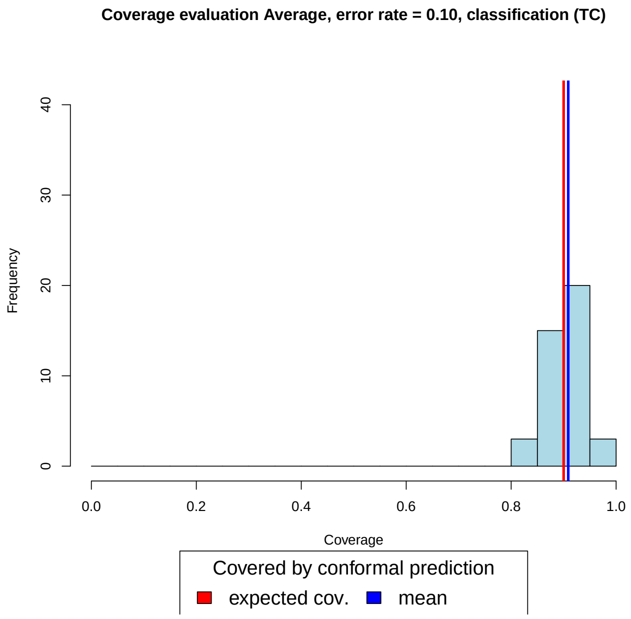

(a) True-class score

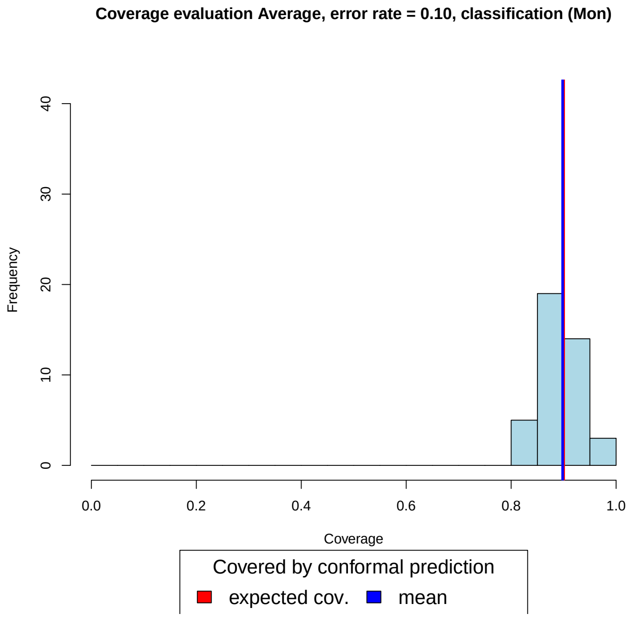

(b) Mondrian Score

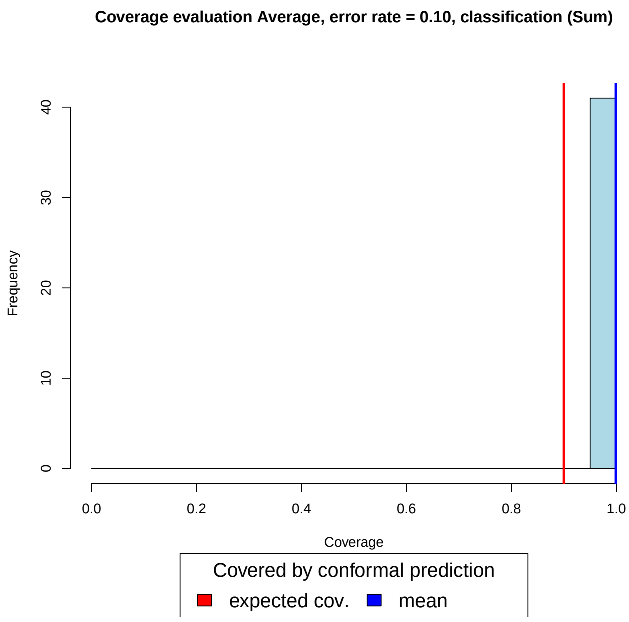

(c) Summation score

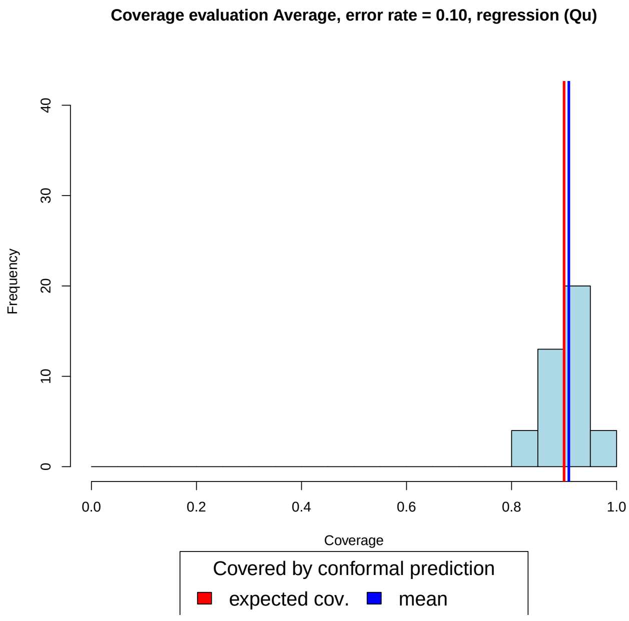

(d) Quantile score

Figure 4: **Coverage evaluation for CP models of 41 drugs from the GDSC1 database trained using CMax viability values and a two-class classification setting.** This figure depicts histograms of the coverage property across CP models for 41 drugs obtained from the GDSC1 database. The coverage is computed as the fraction of cell lines from the test set of each drug, for which the true response was part of the predicted set / interval. The expected coverage of 0.9 for the employed error rate of $\alpha = 0.1$ is shown in red, the actual mean coverage over all investigated drugs is shown in blue. Sub-Figures (a), (b) and (c) depict histograms for the classification setting using the True-class (TC), Mondrian (Mon) and Summation (Sum) scoring functions, respectively. Sub-Figure (d) shows histograms for the regressions setting, where CP was performed using the Quantile (Qu) scoring function.

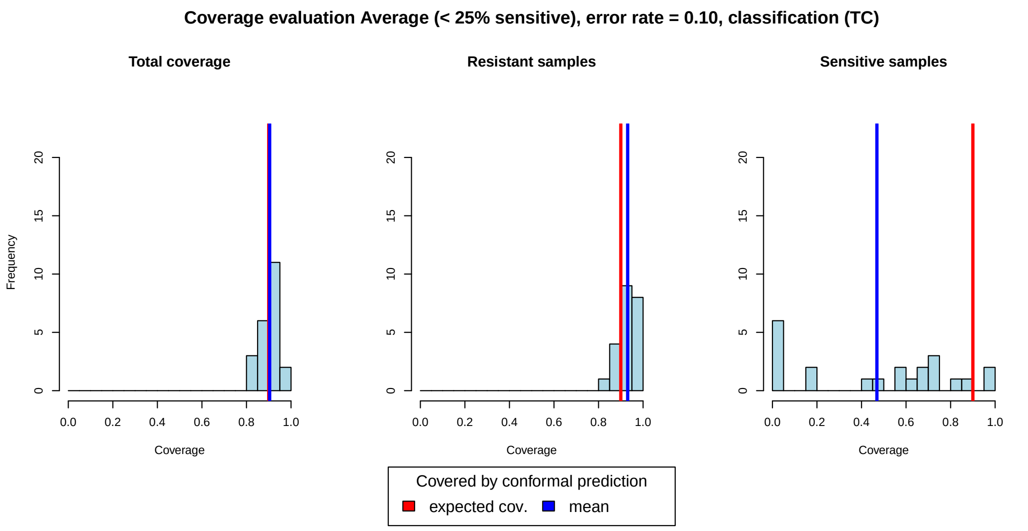

(a) True-class score

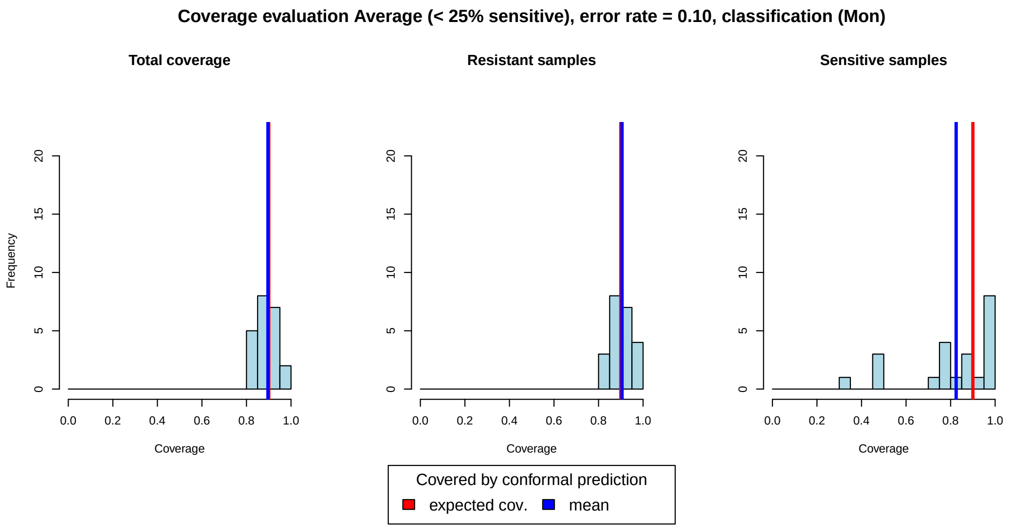

(b) Mondrian score

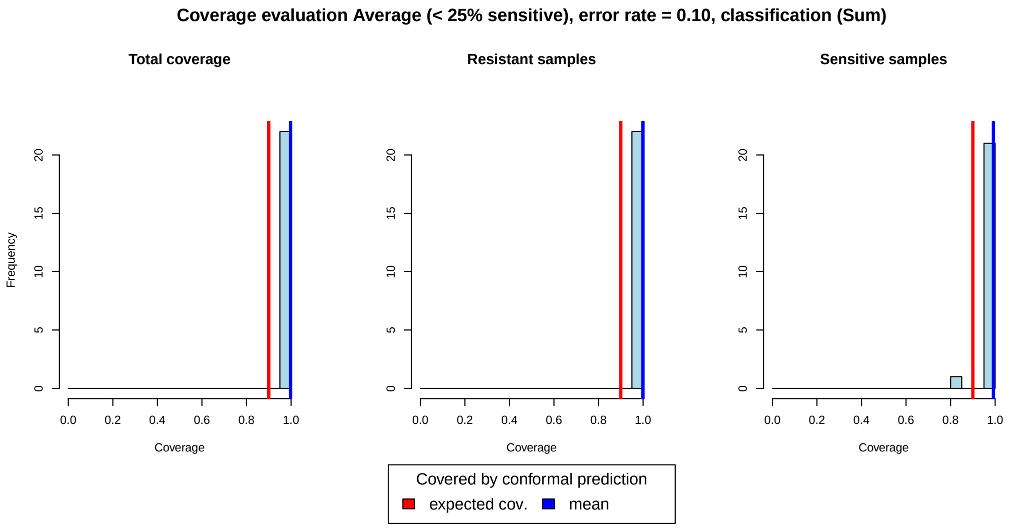

(c) Summation score

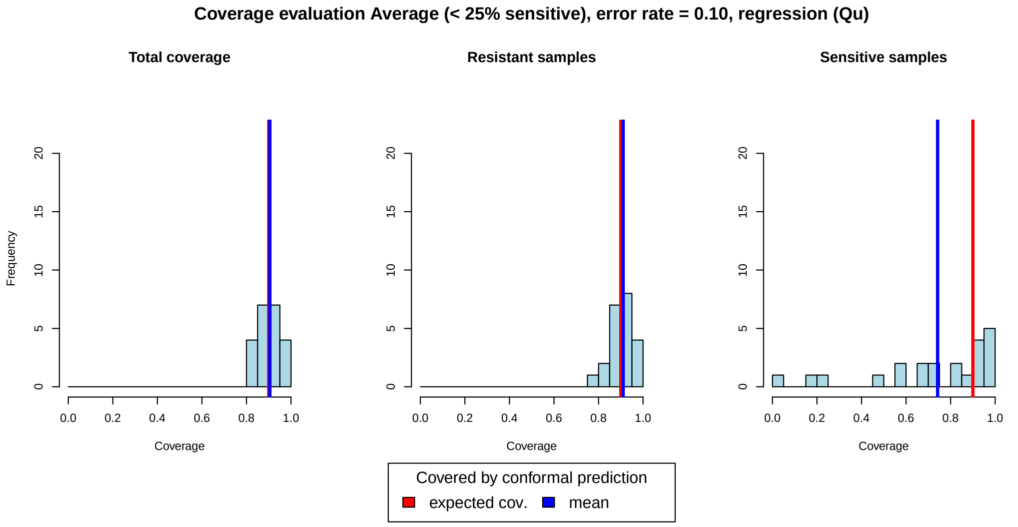

(d) Quantile score

Figure 5: **Coverage evaluation for CP models of 22 drugs from the GDSC1 database trained using CMax viability values and a two-class classification setting with underrepresentation of the sensitive class.** This figure depicts histograms of the coverage property across CP models for 22 drugs obtained from the GDSC1 database, for which the number of sensitive cell lines was less than 25%. The left plot in each sub-figure depicts the total coverage, i.e. the fraction of cell lines from the test set of each drug, for which the true response was part of the predicted set / interval. The middle and right plots show the coverage for subsets of resistant and sensitive cell lines for each drug, respectively. The expected coverage of 0.9 for the employed error rate of $\alpha = 0.1$ is shown in red, the actual mean coverage over all investigated drugs is shown in blue. Sub-Figures (a), (b) and (c) depict histograms for the classification setting using the True-class (TC), Mondrian (Mon) and Summation (Sum) scoring functions, respectively. Sub-Figure (d) shows histograms for the regressions setting, where CP was performed using the Quantile (Qu) scoring function.

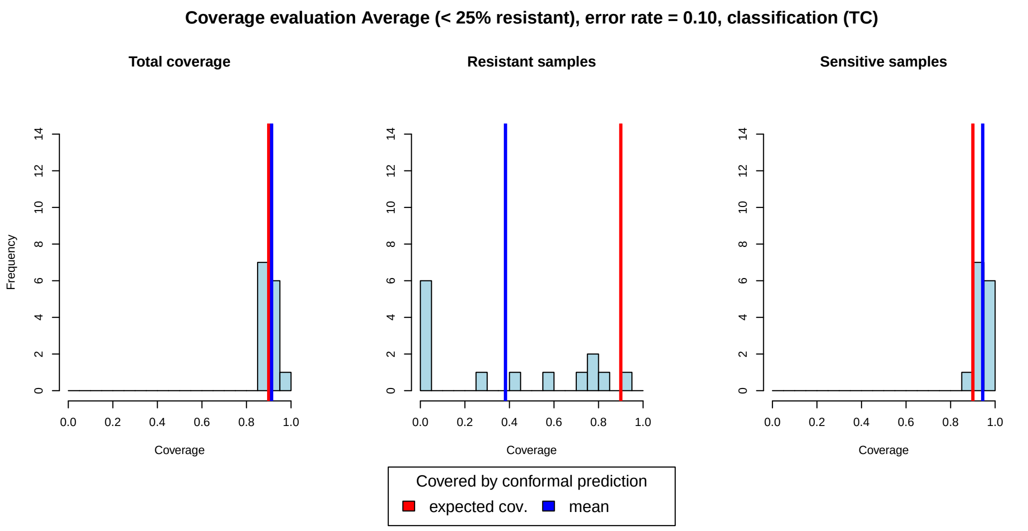

(a) True-class score

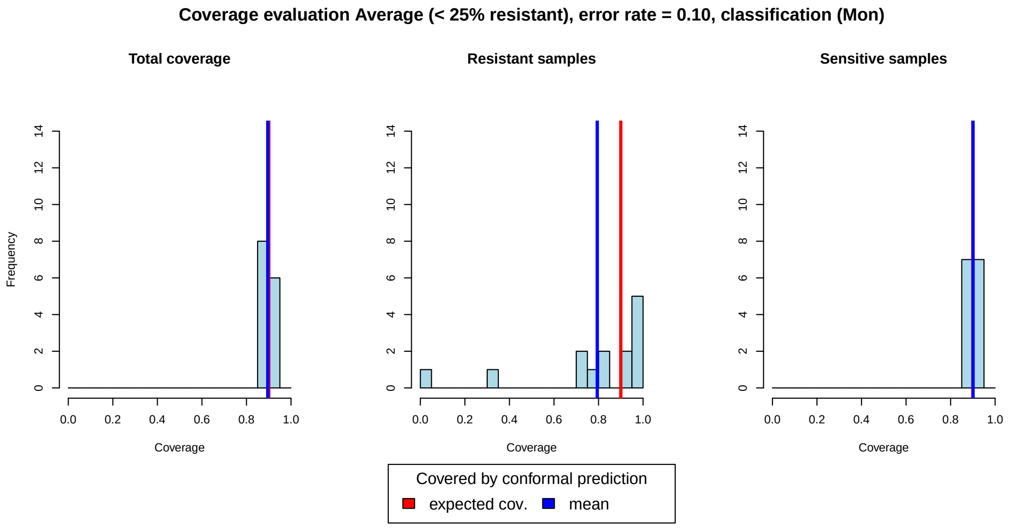

(b) Mondrian score

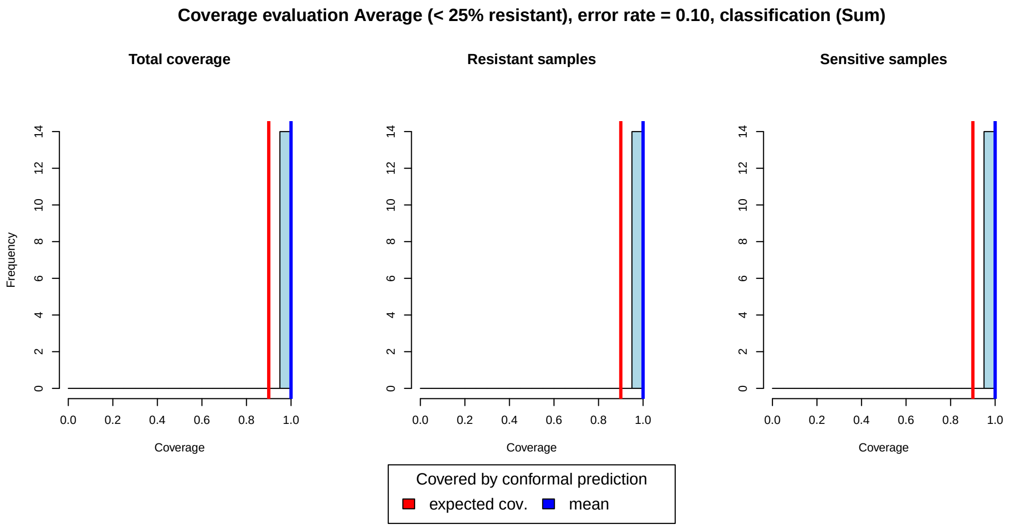

(c) Summation score

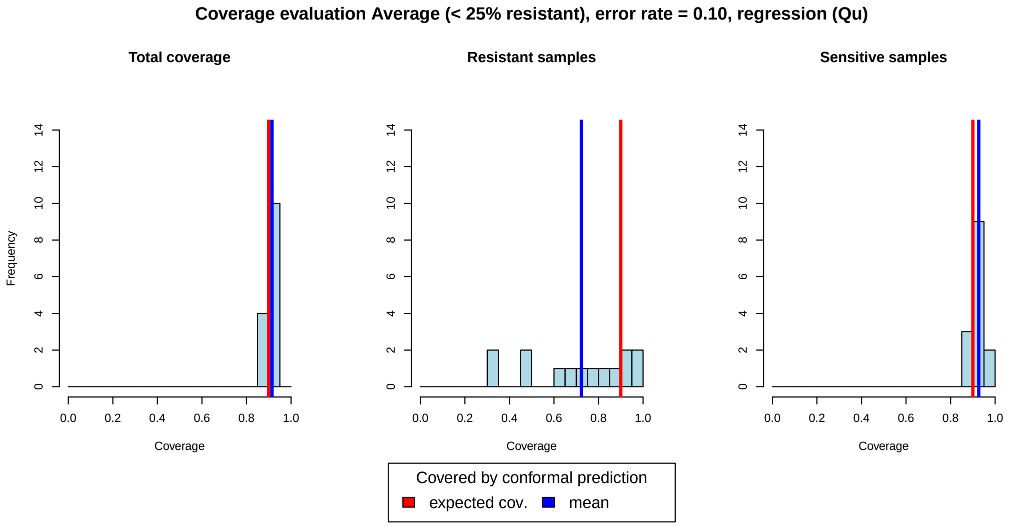

(d) Quantile score

Figure 6: **Coverage evaluation for CP models of 14 drugs from the GDSC1 database trained using CMax viability values and a two-class classification setting with underrepresentation of the resistant class.** This figure depicts histograms of the coverage property across CP models for 14 drugs obtained from the GDSC1 database, for which the number of resistant cell lines was less than 25%. The left plot in each sub-figure depicts the total coverage, i.e. the fraction of cell lines from the test set of each drug, for which the true response was part of the predicted set / interval. The middle and right plots show the coverage for subsets of resistant and sensitive cell lines for each drug, respectively. The expected coverage of 0.9 for the employed error rate of $\alpha = 0.1$ is shown in red, the actual mean coverage over all investigated drugs is shown in blue. Sub-Figures (a), (b) and (c) depict histograms for the classification setting using the True-class (TC), Mondrian (Mon) and Summation (Sum) scoring functions, respectively. Sub-Figure (d) shows histograms for the regressions setting, where CP was performed using the Quantile (Qu) scoring function.

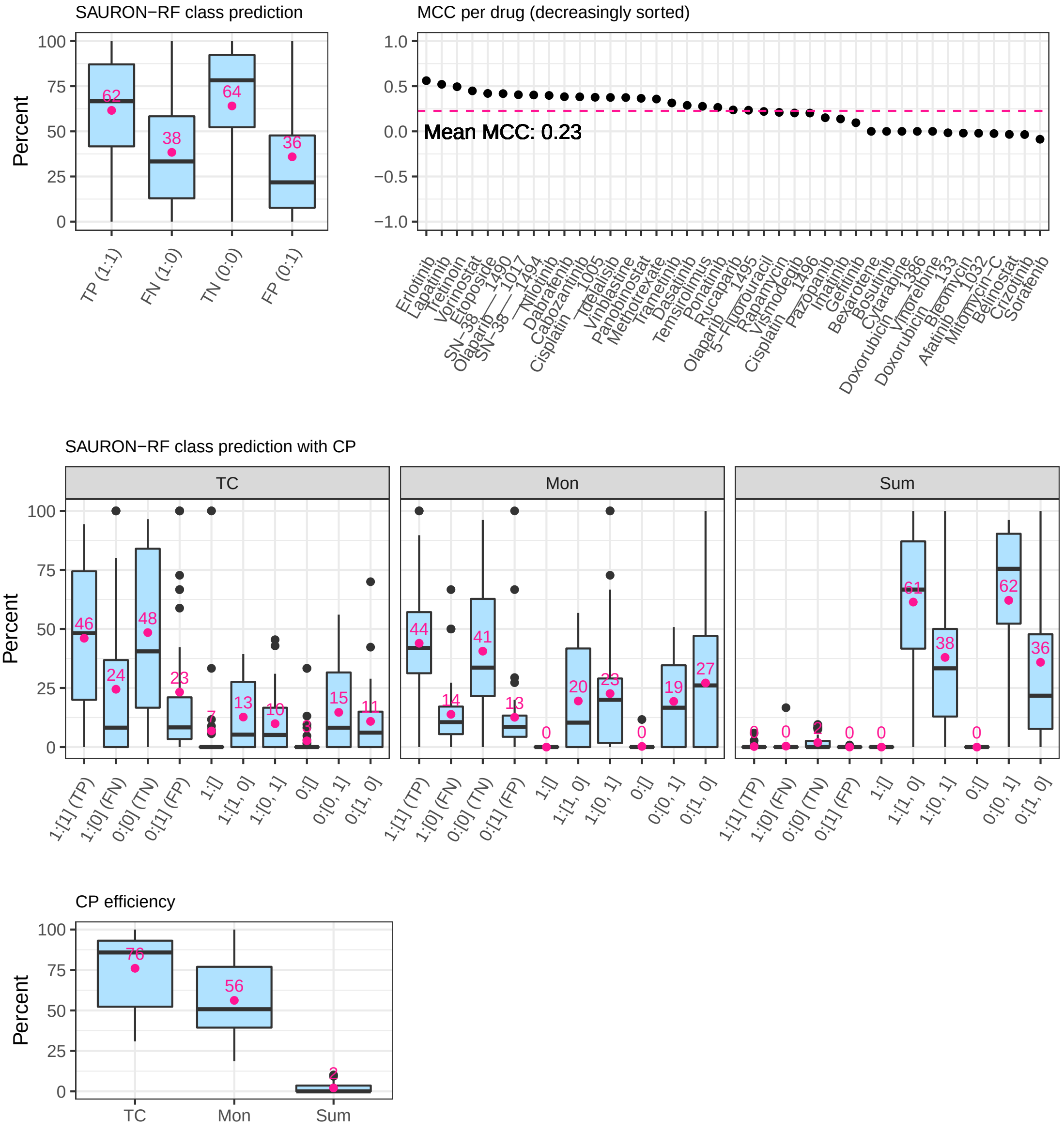

Figure 7: **Classification test set performance for 41 drugs from the GDSC1 database trained using CMax viability values and a two-class classification setting.** The upper row of this figure depicts the classification performance of SAURON-RF across the different drugs. The middle row shows the effects of CP on the performance in terms of true positive/negative predictions. In the lower row, the CP efficiency is presented.

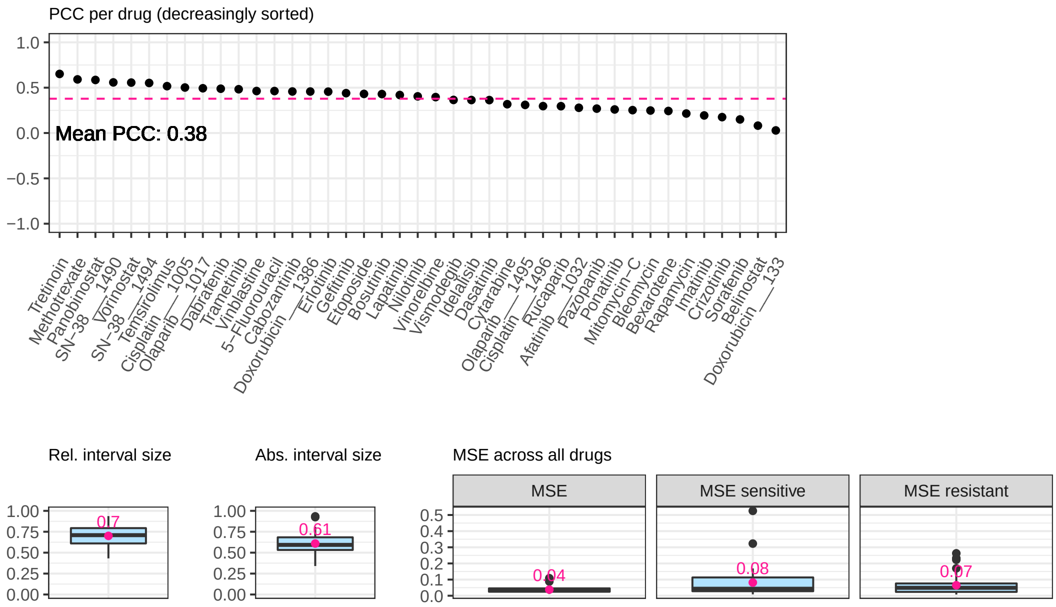

Figure 8: **Regression test set performance for 41 drugs from the GDSC1 database trained using CMax viability values and a two-class classification setting.** The upper row of this figure depicts the Pearson correlation coefficient between the actual continuous response values and the predicted continuous response values for all drugs. The lower row shows the mean-squared error (MSE) and the interval width of the CP Quantile regression score relative to the spanned training ranges of the drugs.

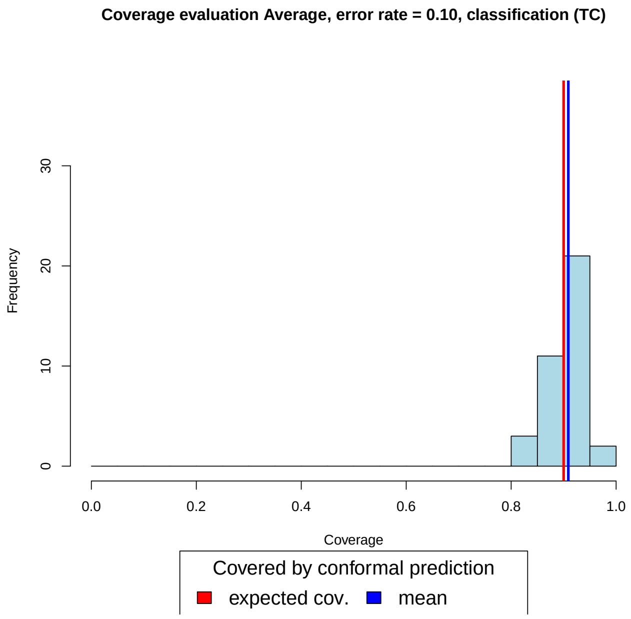

(a) True-class score

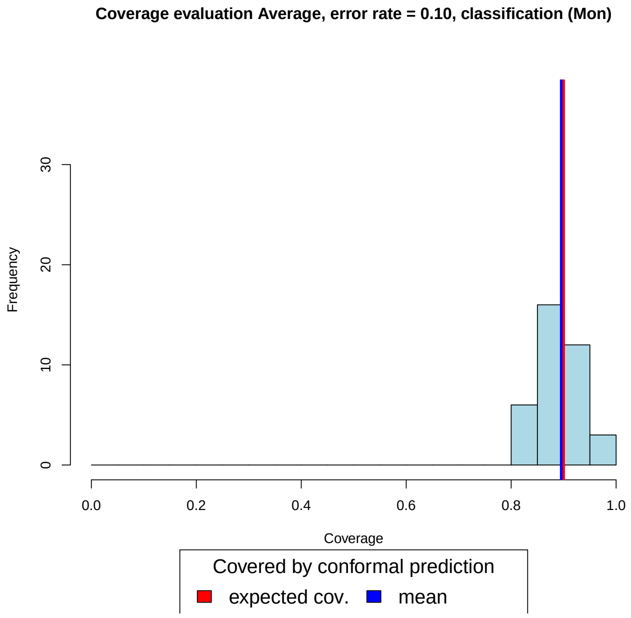

(b) Mondrian score

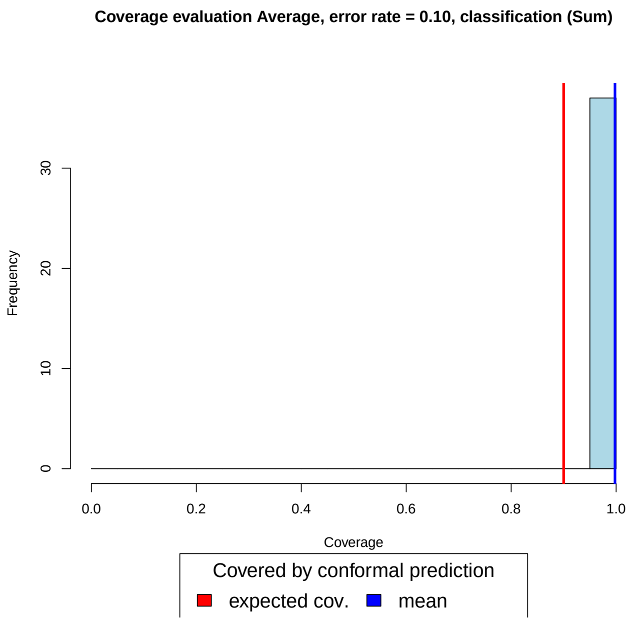

(c) Summation score

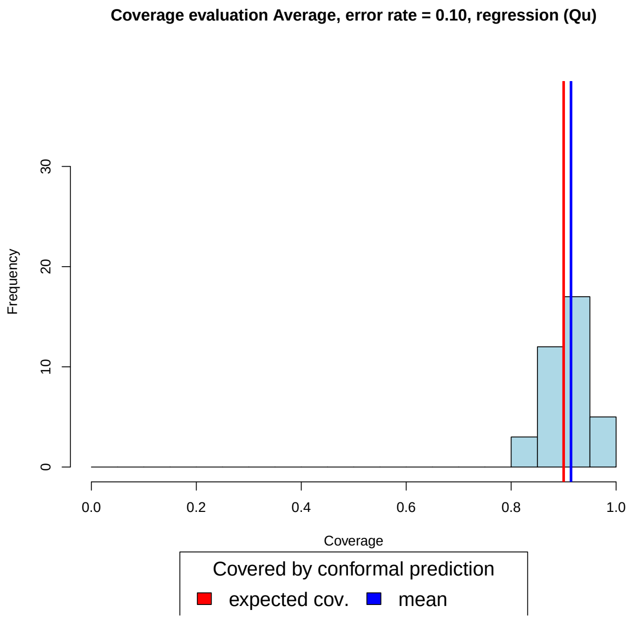

(d) Quantile score

Figure 9: **Coverage evaluation for CP models of 37 drugs from the GDSC1 database trained using CMax viability values and a three-class classification setting.** This figure depicts histograms of the coverage property across CP models for 37 drugs obtained from the GDSC1 database. The coverage is computed as the fraction of cell lines from the test set of each drug, for which the true response was part of the predicted set / interval. The expected coverage of 0.9 for the employed error rate of $\alpha = 0.1$ is shown in red, the actual mean coverage over all investigated drugs is shown in blue. Sub-Figures (a), (b) and (c) depict histograms for the classification setting using the True-class (TC), Mondrian (Mon) and Summation (Sum) scoring functions, respectively. Sub-Figure (d) shows histograms for the regressions setting, where CP was performed using the Quantile (Qu) scoring function.

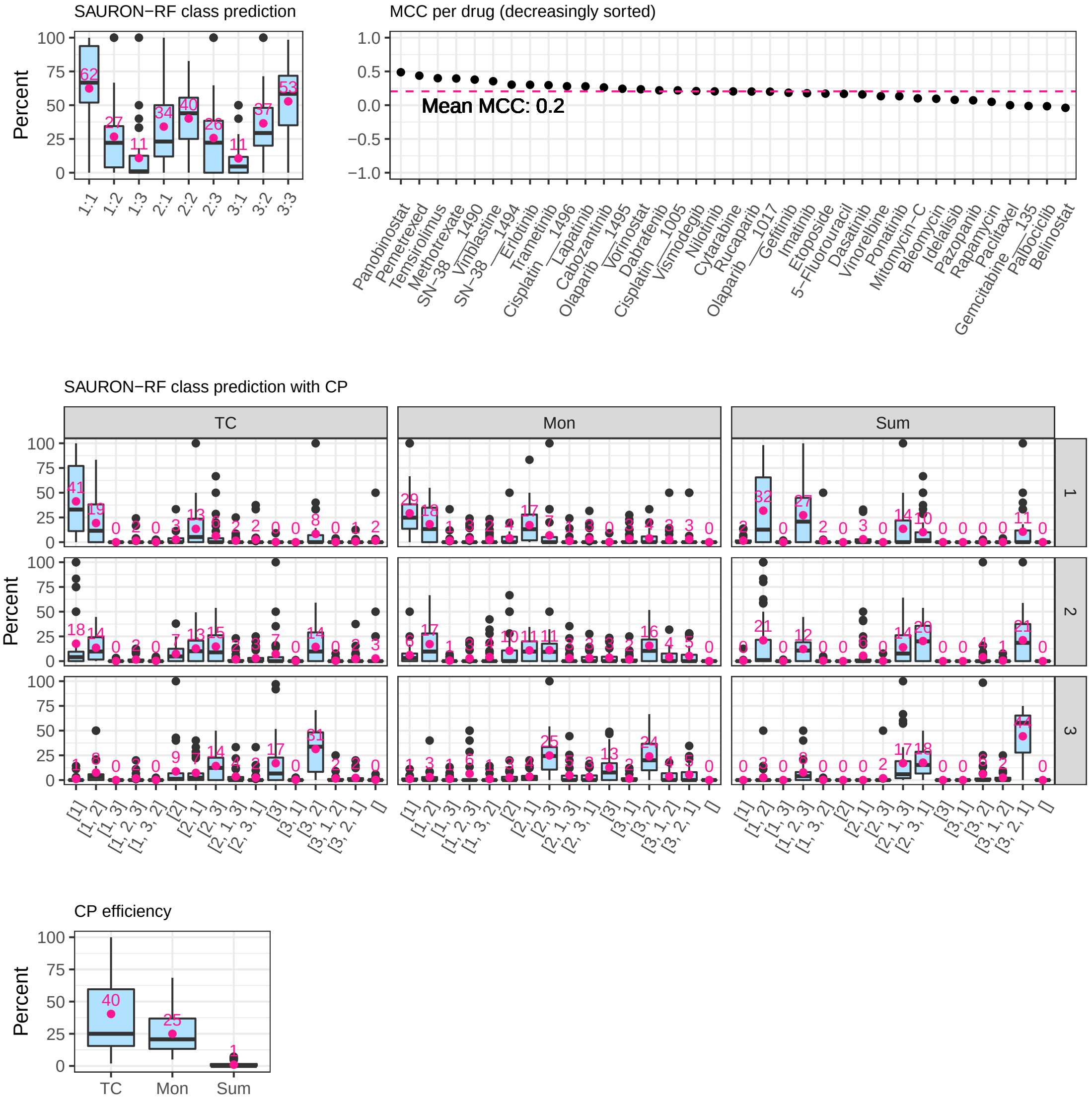

Figure 10: **Classification test set performance for 37 drugs from the GDSC1 database trained using CMax viability values and a three-class classification setting.** The upper row of this figure depicts the classification performance of SAURON-RF across the different drugs from GDSC1. The middle row shows the effects of CP on the performance. In the lower row, the CP efficiency is presented.

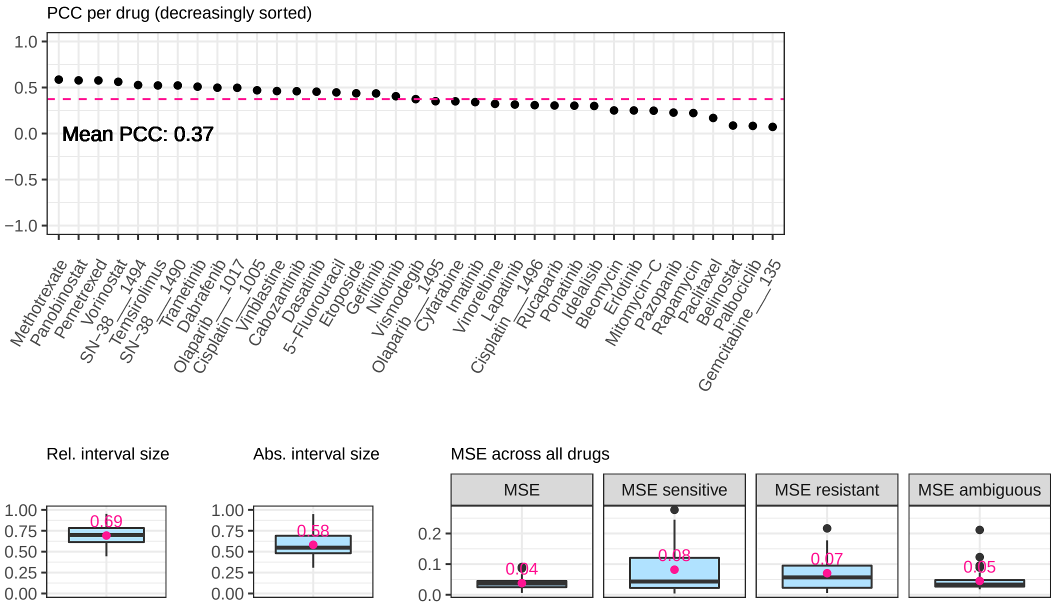

Figure 11: **Regression test set performance for 37 drugs from the GDSC1 database trained using CMax viability values and a three-class classification setting.** The upper row of this figure depicts the Pearson correlation coefficient between the actual continuous response values and the predicted continuous response values for all drugs. The lower row shows the mean-squared error (MSE) and the interval width of the CP Quantile regression score relative to the spanned training ranges of the drugs.

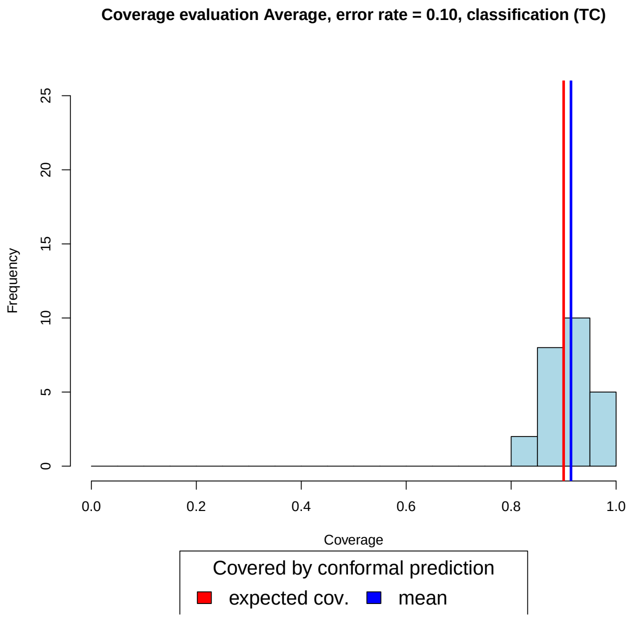

(a) True-class score

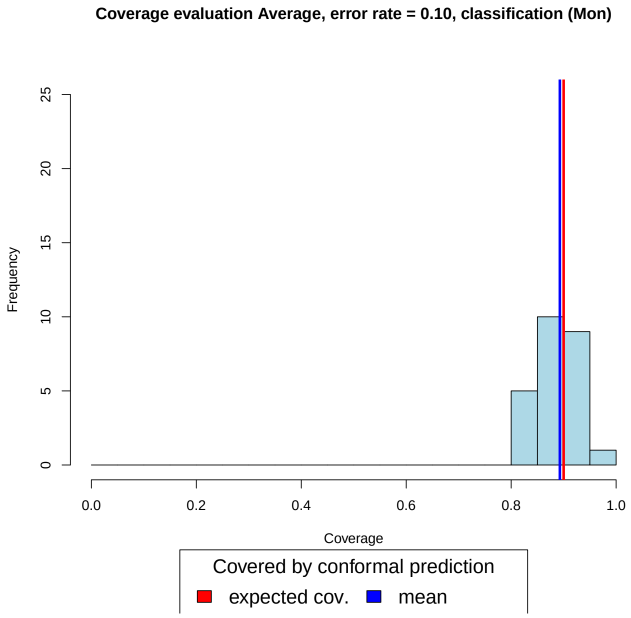

(b) Mondrian score

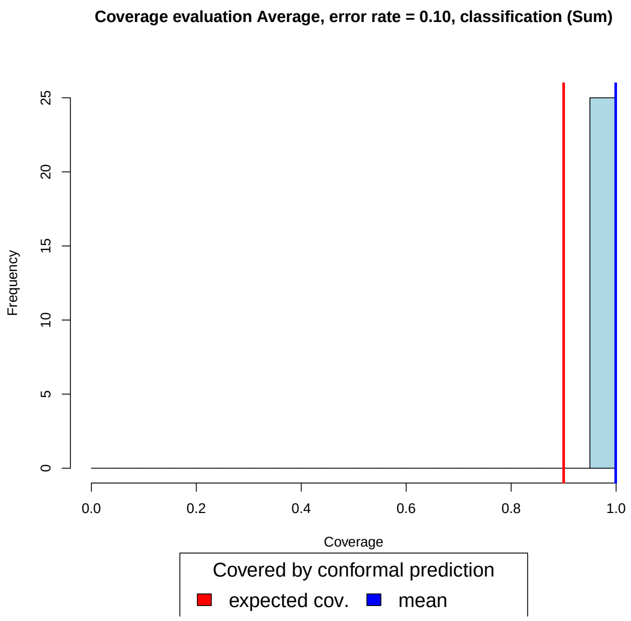

(c) Summation score

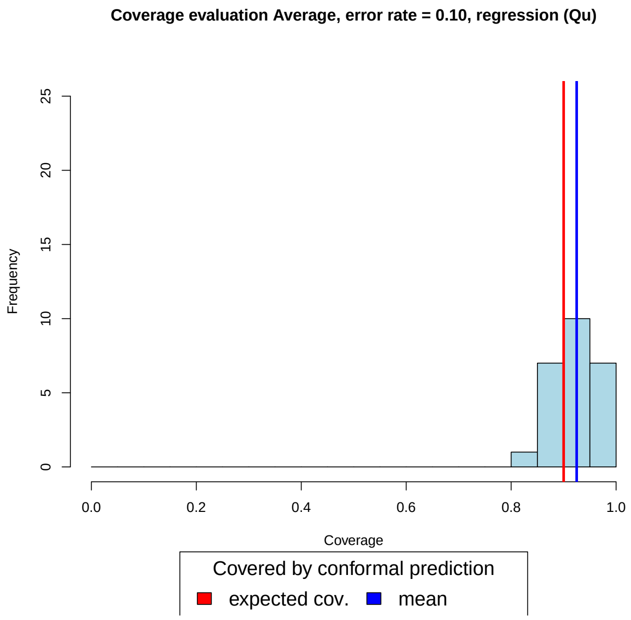

(d) Quantile score

Figure 12: **Coverage evaluation for CP models of 25 drugs from the GDSC1 database trained using CMax viability values and a two-class prioritization setting.** This figure depicts histograms of the coverage property across CP models for 25 drugs obtained from the GDSC1 database, which were used to conduct the drug prioritization analyses in Section 2.4 of the main manuscript. The coverage is computed as the fraction of cell lines from the test set of each drug, for which the true response was part of the predicted set / interval. The expected coverage of 0.9 for the employed error rate of $\alpha = 0.1$ is shown in red, the actual mean coverage over all investigated drugs is shown in blue. Sub-Figures (a), (b) and (c) depict histograms for the classification setting using the True-class (TC), Mondrian (Mon) and Summation (Sum) scoring functions, respectively. Sub-Figure (d) shows histograms for the regressions setting, where CP was performed using the Quantile (Qu) scoring function.

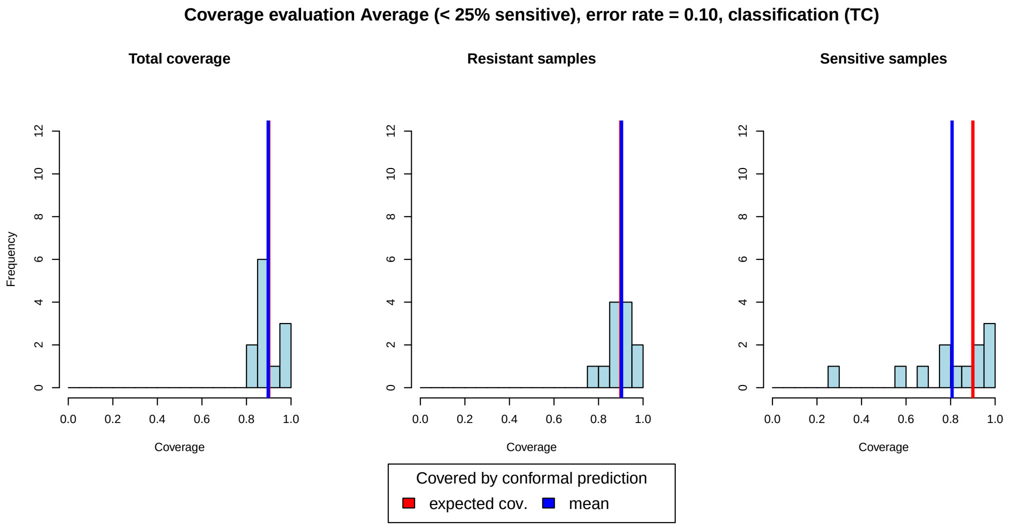

(a) True-class score

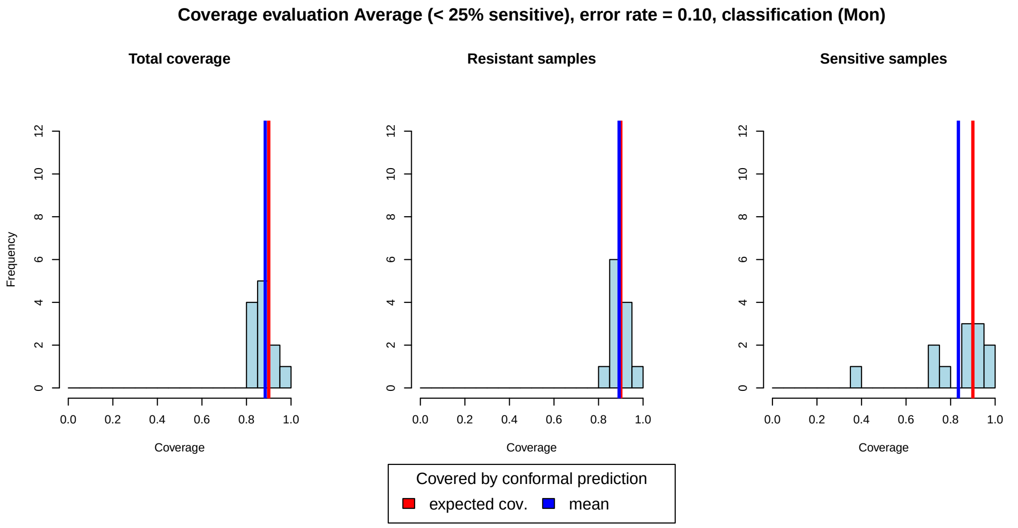

(b) Mondrian score

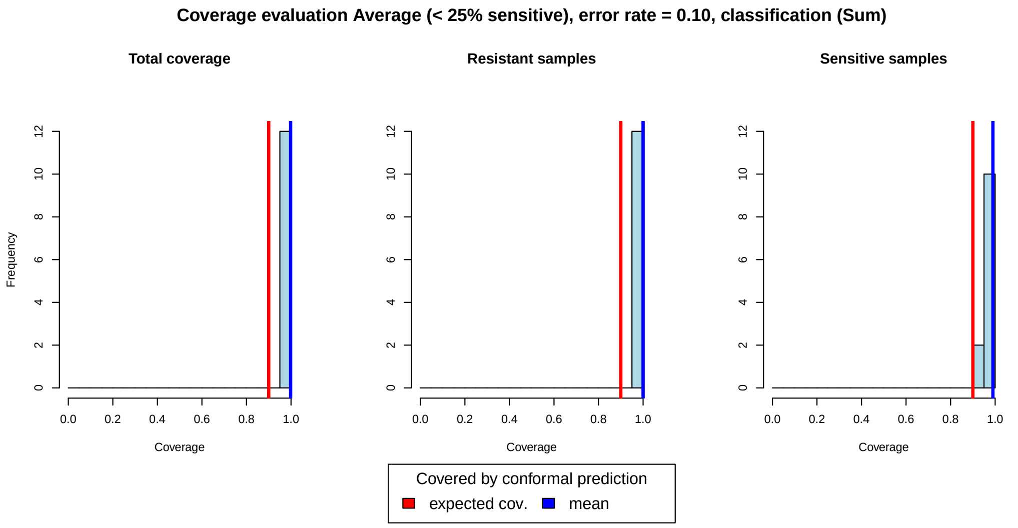

(c) Summation score

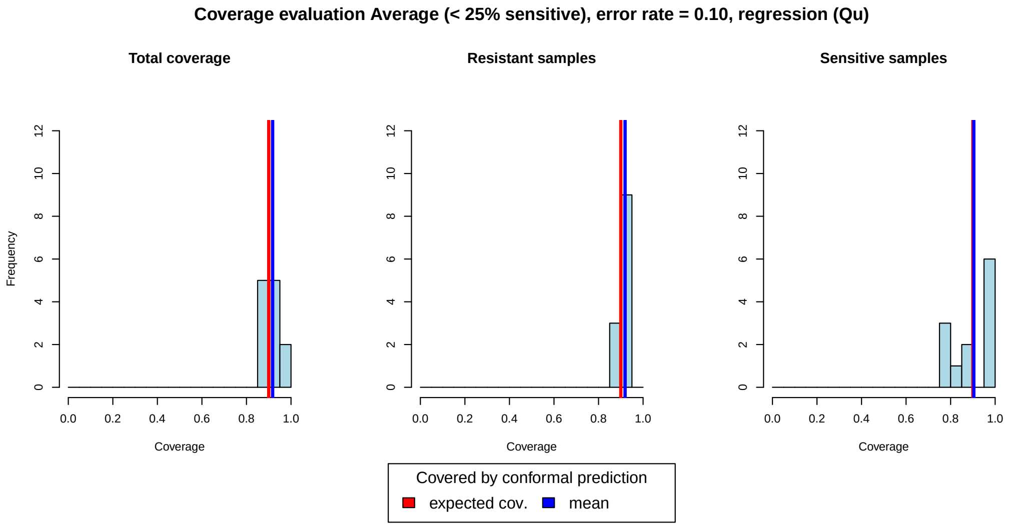

(d) Quantile score

Figure 13: **Coverage evaluation for CP models of twelve drugs from the GDSC1 database trained using CMax viability values and a two-class prioritization setting with underrepresentation of the sensitive class.** This figure depicts histograms of the coverage property across CP models for twelve drugs obtained from the GDSC1 database, for which the number of sensitive cell lines was less than 25%. These drugs were used to conduct the drug prioritization analyses in Section 2.4 of the main manuscript. The left plot in each sub-figure depicts the total coverage, i.e. the fraction of cell lines from the test set of each drug, for which the true response was part of the predicted set / interval. The middle and right plots show the coverage for subsets of resistant and sensitive cell lines for each drug, respectively. The expected coverage of 0.9 for the employed error rate of $\alpha = 0.1$ is shown in red, the actual mean coverage over all investigated drugs is shown in blue. Sub-Figures (a), (b) and (c) depict histograms for the classification setting using the True-class (TC), Mondrian (Mon) and Summation (Sum) scoring functions, respectively. Sub-Figure (d) shows histograms for the regressions setting, where CP was performed using the Quantile (Qu) scoring function.

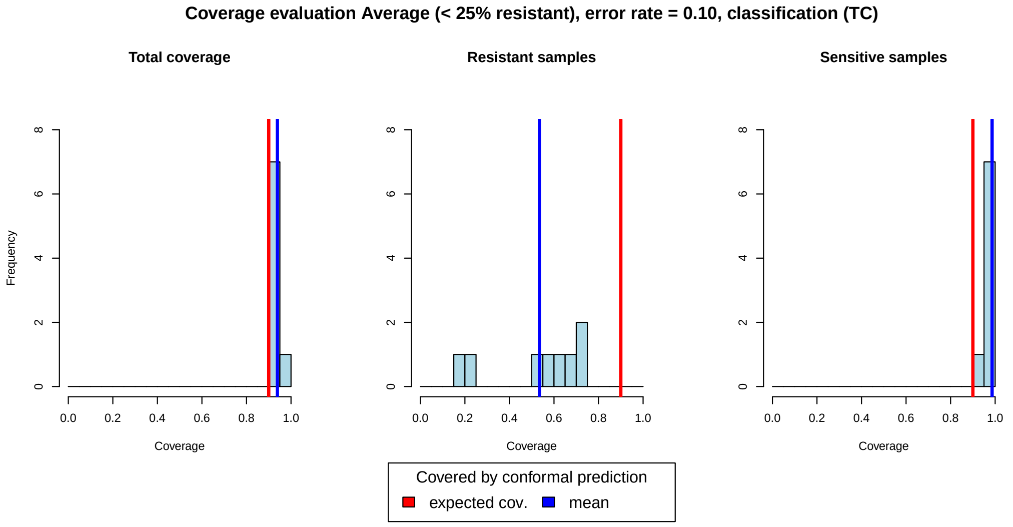

(a) True-class score

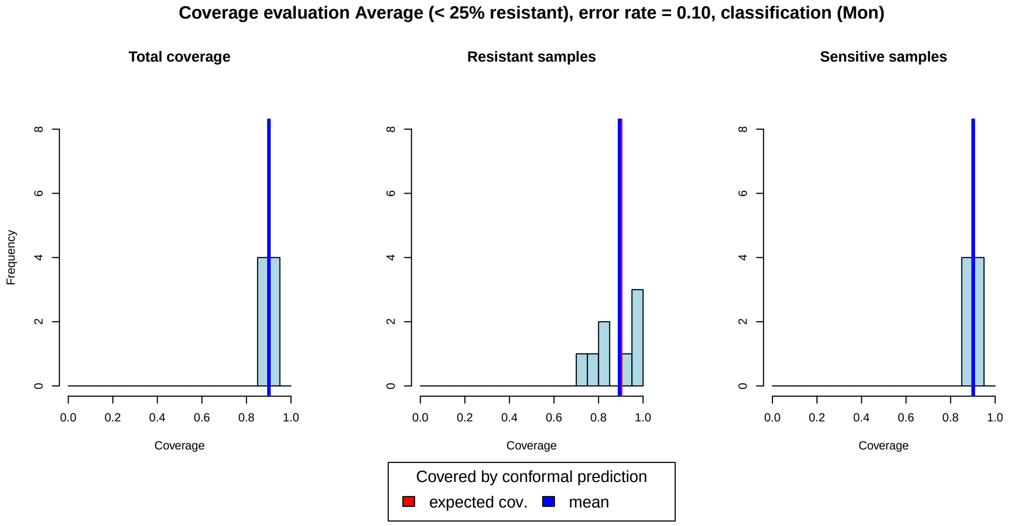

(b) Mondrian score

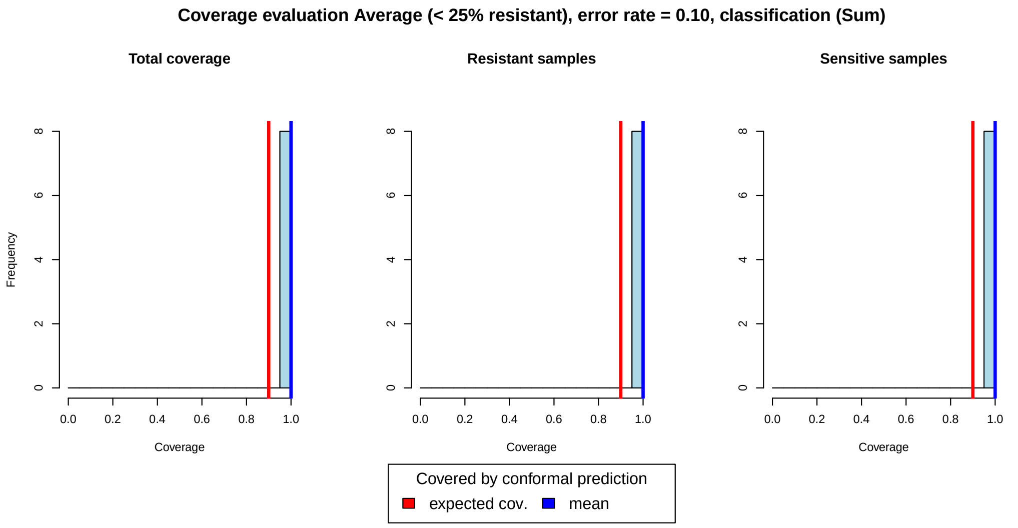

(c) Summation score

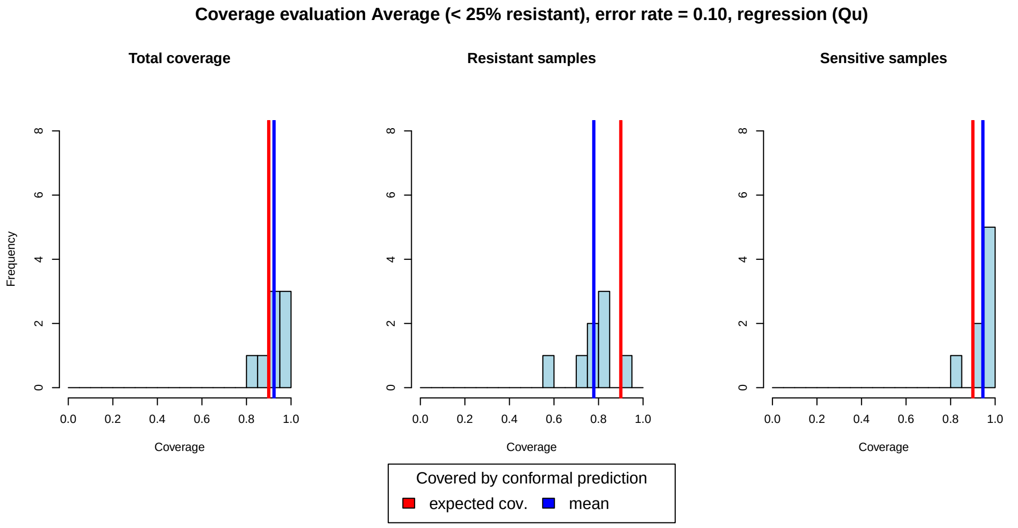

(d) Quantile score

Figure 14: **Coverage evaluation for CP models of eight drugs from the GDSC1 database trained using CMax viability values and a two-class prioritization setting with underrepresentation of the resistant class.** This figure depicts histograms of the coverage property across CP models for eight drugs obtained from the GDSC1 database, for which the number of resistant cell lines was less than 25%. These drugs were used to conduct the drug prioritization analyses in Section 2.4 of the main manuscript. The left plot in each sub-figure depicts the total coverage, i.e. the fraction of cell lines from the test set of each drug, for which the true response was part of the predicted set / interval. The middle and right plots show the coverage for subsets of resistant and sensitive cell lines for each drug, respectively. The expected coverage of 0.9 for the employed error rate of $\alpha = 0.1$ is shown in red, the actual mean coverage over all investigated drugs is shown in blue. Sub-Figures (a), (b) and (c) depict histograms for the classification setting using the True-class (TC), Mondrian (Mon) and Summation (Sum) scoring functions, respectively. Sub-Figure (d) shows histograms for the regressions setting, where CP was performed using the Quantile (Qu) scoring function.

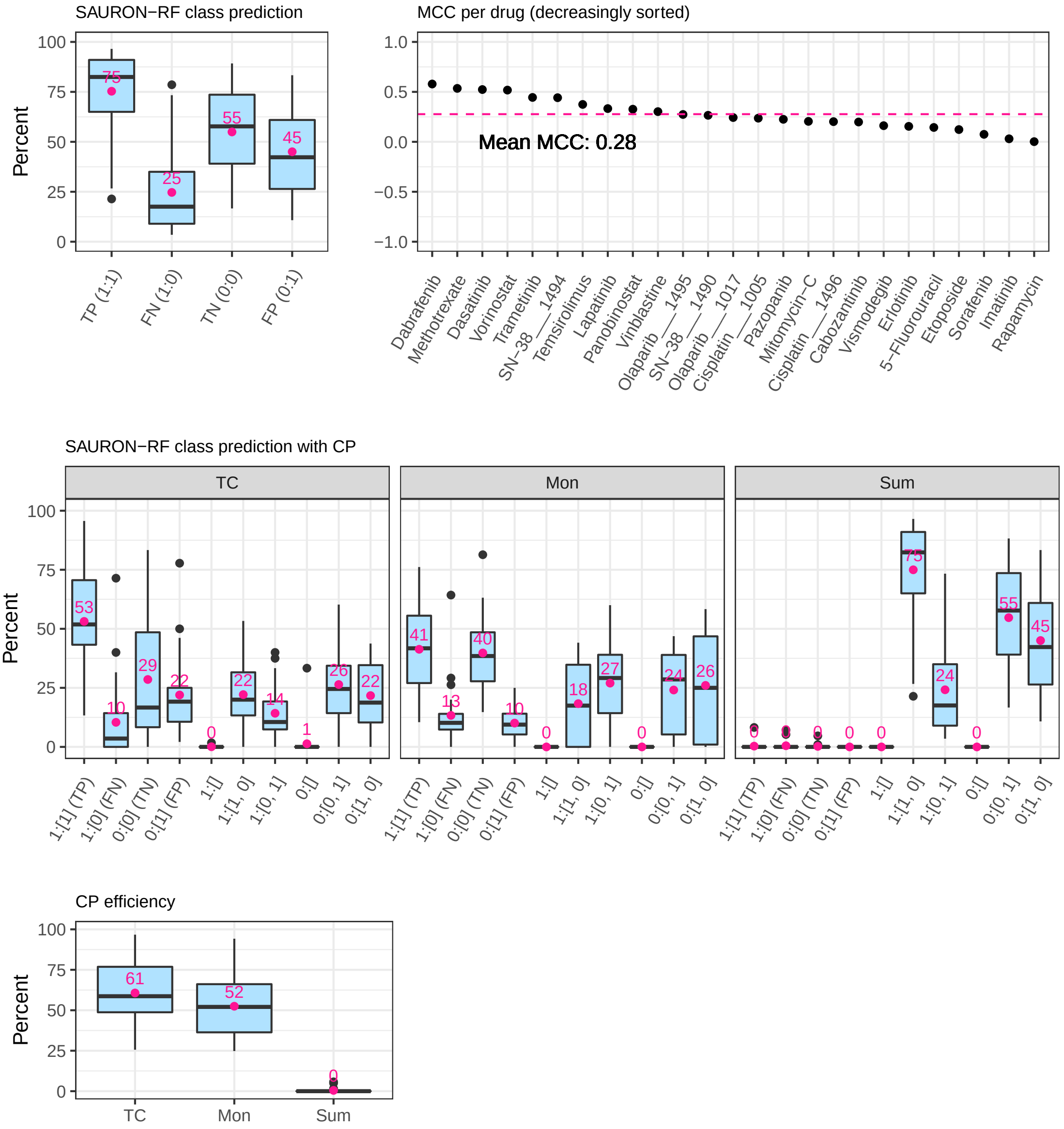

Figure 15: **Classification test set performance for 25 drugs from the GDSC1 database trained using CMax viability values and a two-class prioritization setting.** The upper row of this figure depicts the classification performance of SAURON-RF across for all GDSC1 drugs, which were used to conduct the drug prioritization analyses in Section 2.4 of the main manuscript. The middle row shows the effects of CP on the performance in terms of true positive/negative predictions. In the lower row, the CP efficiency is presented.

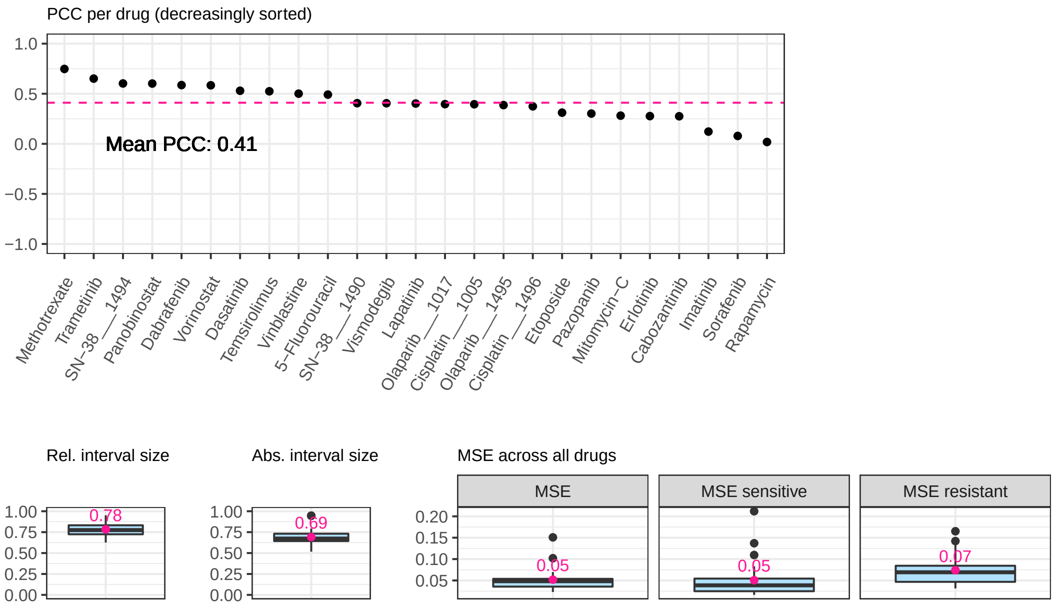

Figure 16: **Regression test set performance for 25 drugs from the GDSC1 database trained using CMax viability values and a two-class prioritization setting.** The upper row of this figure depicts the Pearson correlation coefficient between the actual continuous response values and the predicted continuous response values for all GDSC1 drugs, which were used to conduct the drug prioritization analyses in Section 2.4 of the main manuscript. The lower row shows the mean-squared error (MSE) and the interval width of the CP Quantile regression score relative to the spanned training ranges of the drugs.

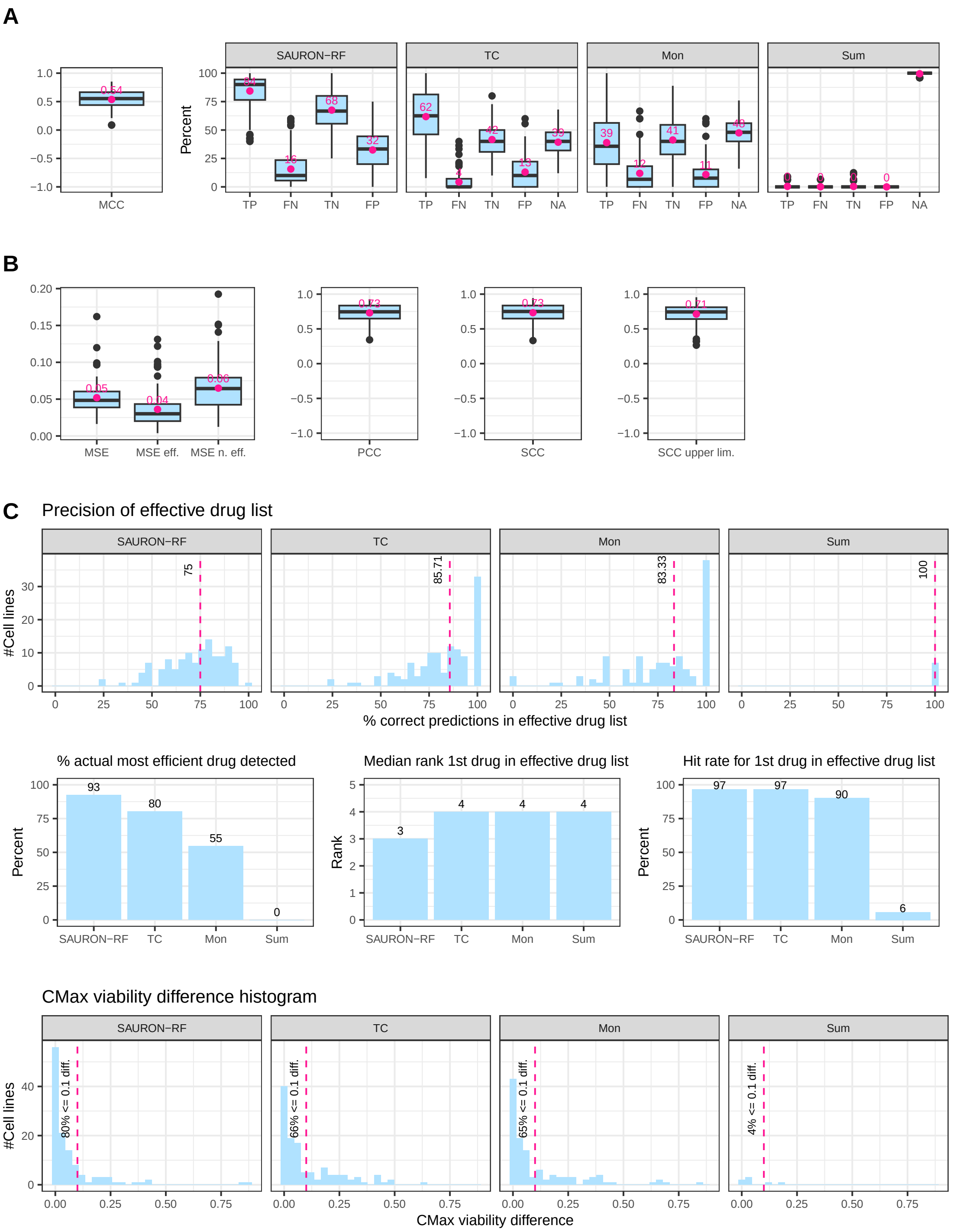

Figure 17: **Prioritization results across all test cell lines of GDSC1.** In A, we show the classification performance of SAURON-RF with and without our CP pipeline. B depicts the regression performance in terms of MSE, PCC and SCC. Here, the MSE is given for the effective drugs, the ineffective drugs, and all drugs. We provide the SCC for the predicted values using SAURON-RF only (SCC) and the upper limit of the CP interval (SCC upper lim.). In C, we plot various measures to evaluate our prioritized drug lists. The upper row of C depicts the precision of SAURON-RF without (SAURON-RF class + SAURON-RF continuous prediction) and with CP (TC + upper limit, Mon + upper limit, Sum + upper limit). In the middle row, we show the percentage of cell lines for which the most efficient drug was detected, the median rank of the first drug in our effective drug list and the percentage of cell lines for which this prediction was a TP. The CMax viability difference between our first drug and the actual first drug is depicted in the lower row.

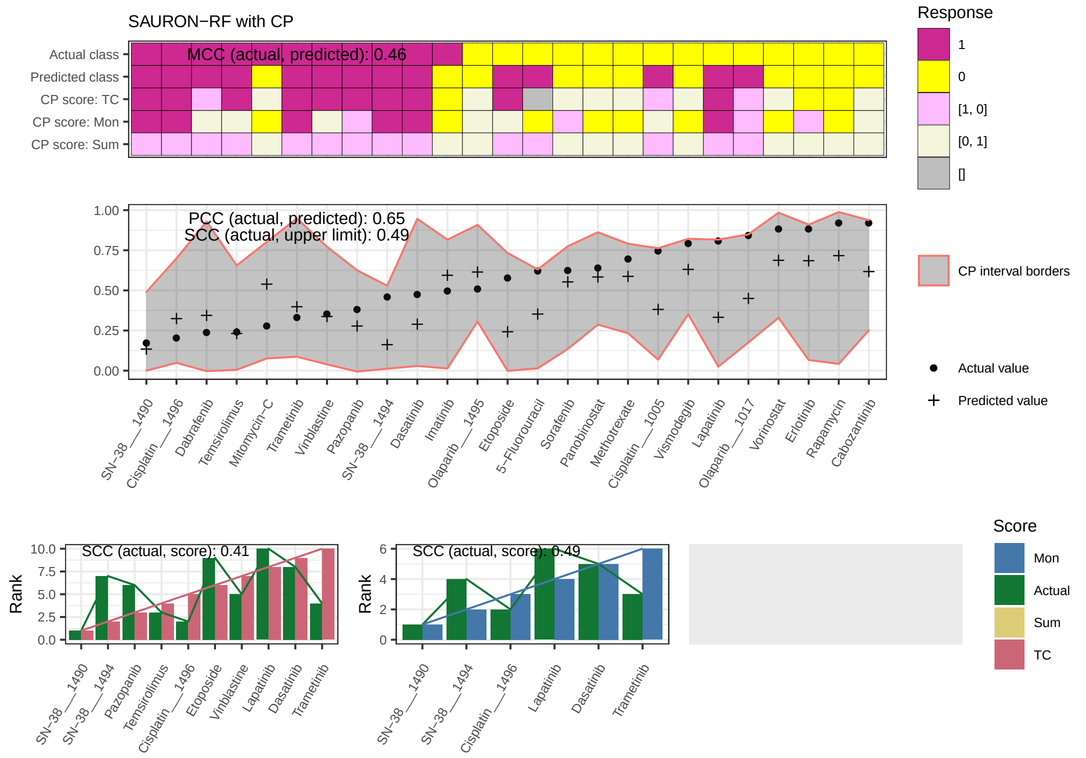

Figure 18: **Prioritization example GDSC1.**This figure exemplifies the performance of our prioritization pipeline (cf. Figure 2 in main manuscript) when applied to one particular cell line (COSMIC ID 753532) from the test set of the GDSC1 data set. The upper plot visualizes the classification performance with and without CP for all analyzed drugs. The middle plot depicts the regression result for all drugs, including the 90% CP interval, and the lower plot shows the resulting prioritized drug lists with the drugs ascendingly sorted by their upper CP limit prediction.

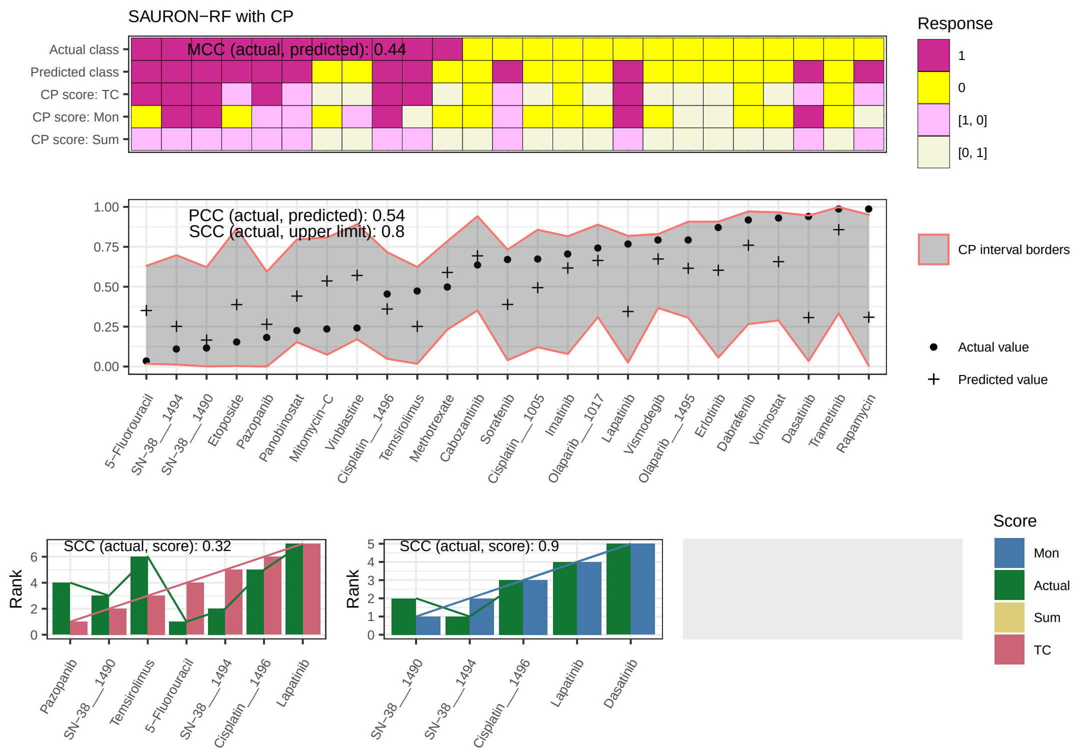

Figure 19: **Prioritization example GDSC1.**This figure exemplifies the performance of our prioritization pipeline (cf. Figure 2 in main manuscript) when applied to one particular cell line (COSMIC ID 905941) from the test set of the GDSC1 data set. The upper plot visualizes the classification performance with and without CP for all analyzed drugs. The middle plot depicts the regression result for all drugs, including the 90% CP interval, and the lower plot shows the resulting prioritized drug lists with the drugs ascendingly sorted by their upper CP limit prediction.

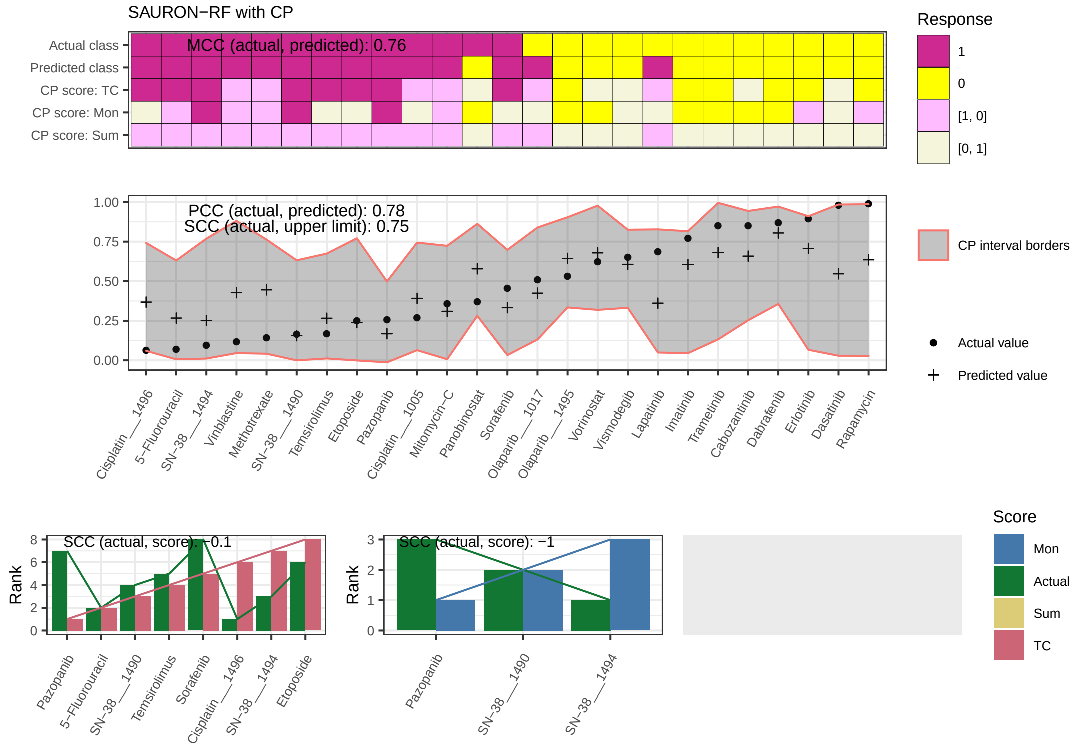

Figure 20: **Prioritization example GDSC1.**This figure exemplifies the performance of our prioritization pipeline (cf. Figure 2 in main manuscript) when applied to one particular cell line (COSMIC ID 905942) from the test set of the GDSC1 data set. The upper plot visualizes the classification performance with and without CP for all analyzed drugs. The middle plot depicts the regression result for all drugs, including the 90% CP interval, and the lower plot shows the resulting prioritized drug lists with the drugs ascendingly sorted by their upper CP limit prediction.

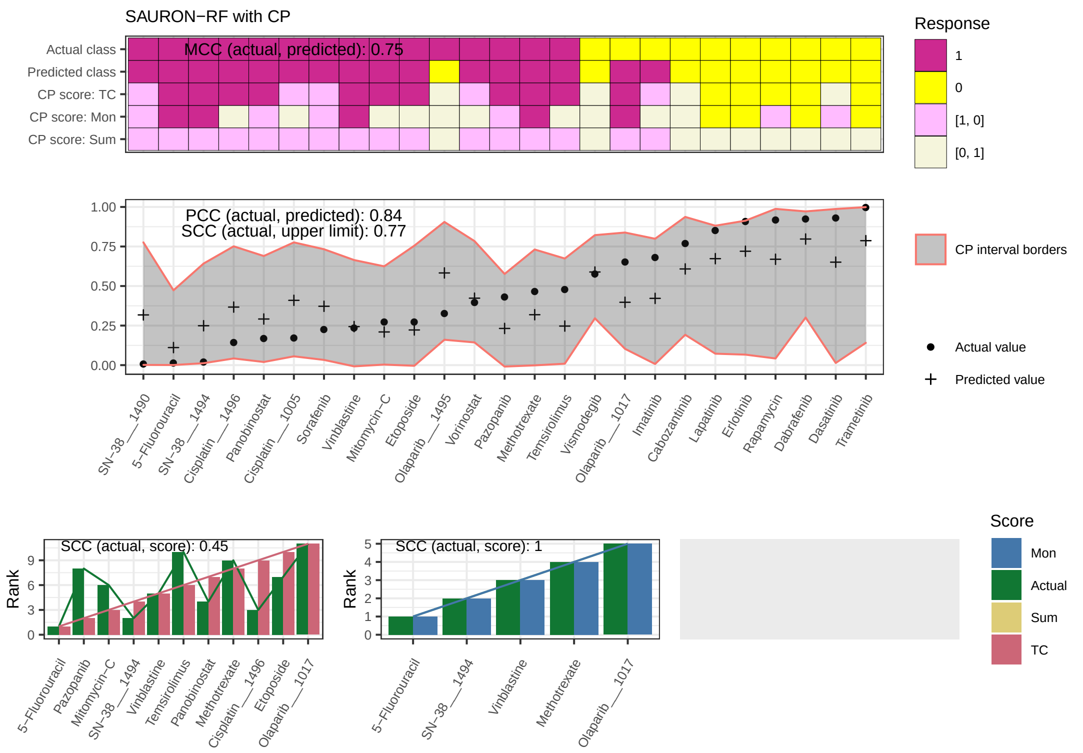

Figure 21: **Prioritization example GDSC1.**This figure exemplifies the performance of our prioritization pipeline (cf. Figure 2 in main manuscript) when applied to one particular cell line (COSMIC ID 906875) from the test set of the GDSC1 data set. The upper plot visualizes the classification performance with and without CP for all analyzed drugs. The middle plot depicts the regression result for all drugs, including the 90% CP interval, and the lower plot shows the resulting prioritized drug lists with the drugs ascendingly sorted by their upper CP limit prediction.

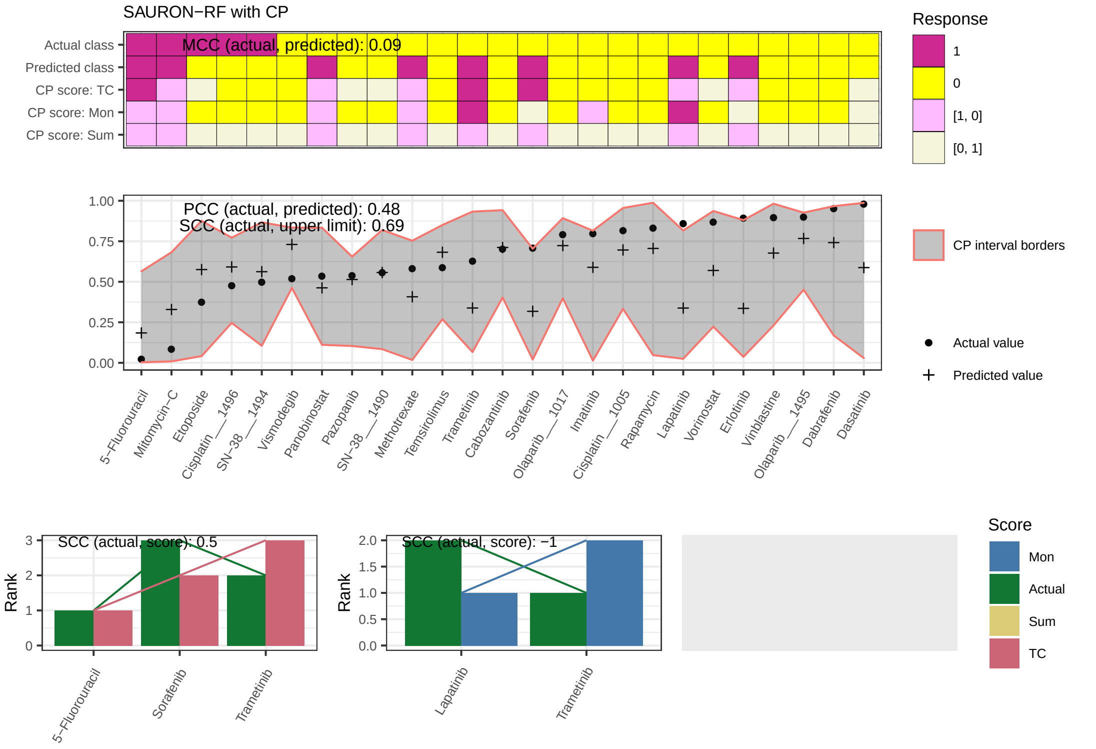

Figure 22: **Prioritization example GDSC1.**This figure exemplifies the performance of our prioritization pipeline (cf. Figure 2 in main manuscript) when applied to one particular cell line (COSMIC ID 917486) from the test set of the GDSC1 data set. The upper plot visualizes the classification performance with and without CP for all analyzed drugs. The middle plot depicts the regression result for all drugs, including the 90% CP interval, and the lower plot shows the resulting prioritized drug lists with the drugs ascendingly sorted by their upper CP limit prediction.
